# Phenotypic characterization of disease‐initiating stem cells in *JAK2*‐ or *CALR*‐mutated myeloproliferative neoplasms

**DOI:** 10.1002/ajh.26889

**Published:** 2023-03-09

**Authors:** Daniel Ivanov, Jelena D. Milosevic Feenstra, Irina Sadovnik, Harald Herrmann, Barbara Peter, Michael Willmann, Georg Greiner, Katharina Slavnitsch, Emir Hadzijusufovic, Thomas Rülicke, Maik Dahlhoff, Gregor Hoermann, Sigrid Machherndl‐Spandl, Gregor Eisenwort, Michael Fillitz, Thamer Sliwa, Maria‐Theresa Krauth, Peter Bettelheim, Wolfgang R. Sperr, Elisabeth Koller, Michael Pfeilstöcker, Heinz Gisslinger, Felix Keil, Robert Kralovics, Peter Valent

**Affiliations:** ^1^ Department of Internal Medicine I, Division of Hematology and Hemostaseology Medical University of Vienna Vienna Austria; ^2^ Ludwig Boltzmann Institute for Hematology and Oncology Medical University of Vienna Vienna Austria; ^3^ Department of Radiation Oncology Medical University of Vienna Vienna Austria; ^4^ Department for Companion Animals, Clinical Unit for Internal Medicine University of Veterinary Medicine Vienna Vienna Austria; ^5^ Department of Laboratory Medicine Medical University of Vienna Vienna Austria; ^6^ Ihr Labor, Medical Diagnostic Laboratories Vienna Austria; ^7^ Institute of in vivo and in vitro Models University of Veterinary Medicine Vienna Vienna Austria; ^8^ Department of Biomedical Sciences University of Veterinary Medicine Vienna Vienna Austria; ^9^ MLL Munich Leukemia Laboratory Munich Germany; ^10^ Hospital Ordensklinikum Elisabethinen Linz Linz Austria; ^11^ Johannes Kepler University, Medical Faculty Linz Austria; ^12^ Third Medical Department for Hematology and Oncology Hanusch Hospital Vienna Vienna Austria

## Abstract

Myeloproliferative neoplasms (MPN) are characterized by uncontrolled expansion of myeloid cells, disease‐related mutations in certain driver‐genes including *JAK2*, *CALR*, and *MPL*, and a substantial risk to progress to secondary acute myeloid leukemia (sAML). Although behaving as stem cell neoplasms, little is known about disease‐initiating stem cells in MPN. We established the phenotype of putative CD34^+^/CD38^−^ stem cells and CD34^+^/CD38^+^ progenitor cells in MPN. A total of 111 patients with MPN suffering from polycythemia vera, essential thrombocythemia, or primary myelofibrosis (PMF) were examined. In almost all patients tested, CD34^+^/CD38^−^ stem cells expressed CD33, CD44, CD47, CD52, CD97, CD99, CD105, CD117, CD123, CD133, CD184, CD243, and CD274 (PD‐L1). In patients with PMF, MPN stem cells often expressed CD25 and sometimes also CD26 in an aberrant manner. MPN stem cells did not exhibit substantial amounts of CD90, CD273 (PD‐L2), CD279 (PD‐1), CD366 (TIM‐3), CD371 (CLL‐1), or IL‐1RAP. The phenotype of CD34^+^/CD38^−^ stem cells did not change profoundly during progression to sAML. The disease‐initiating capacity of putative MPN stem cells was confirmed in NSGS mice. Whereas CD34^+^/CD38^−^ MPN cells engrafted in NSGS mice, no substantial engraftment was produced by CD34^+^/CD38^+^ or CD34^−^ cells. The JAK2‐targeting drug fedratinib and the BRD4 degrader dBET6 induced apoptosis and suppressed proliferation in MPN stem cells. Together, MPN stem cells display a unique phenotype, including cytokine receptors, immune checkpoint molecules, and other clinically relevant target antigens. Phenotypic characterization of neoplastic stem cells in MPN and sAML should facilitate their enrichment and the development of stem cell‐eradicating (curative) therapies.

## 1 INTRODUCTION

Myeloproliferative neoplasms (MPN) are a group of stem cell‐derived malignancies characterized by uncontrolled expansion of myeloid cells, elevated blood counts, and a considerable risk to progress to secondary acute myeloid leukemia (sAML).^1^, ^2^, ^3^, ^4^, ^5^, ^6^, ^7^, ^8^ In most patients, a mutation in one of the critical MPN‐related driver genes (*JAK2*/*CALR*/*MPL*) is detected.^2^, ^3^, ^4^, ^5^, ^6^ The classification of the World Health Organization (WHO) recognizes three classical MPN: polycythemia vera (PV), essential thrombocythemia (ET), and primary myelofibrosis (PMF).^9^, ^10^ Each of these MPN variants exhibits unique clinical, morphological, and molecular features.^1^, ^2^, ^3^, ^4^, ^5^, ^6^, ^7^, ^8^, ^9^, ^10^ However, the three MPN also share clinical, molecular, and pathological characteristics, and in several patients, transition from one type into another type of MPN is observed.^4^, ^5^, ^6^, ^7^, ^8^, ^9^, ^10^ MPN‐related morbidity and mortality are emerging challenges in industrialized countries.^5^, ^7^, ^11^, ^12^, ^13^, ^14^ In fact, novel improved diagnostics and better treatment together with an enhanced life expectancy have resulted in an increasing prevalence of MPN.^5^, ^12^, ^13^, ^14^, ^15^ In general, therapeutic options are limited in MPN. For patients with advanced MPN or sAML, the only curative approach is allogeneic hematopoietic stem cell transplantation (HSCT).^16^, ^17^, ^18^ However, this therapy can only be offered to a smaller group of relatively young and fit patients. In all other cases, disease management is based on symptom control and the use of growth‐inhibitory (conventional or targeted) agents, including interferon‐alpha, anagrelide, hydroxyurea, and JAK2‐targeting drugs.^1^, ^5^, ^13^, ^14^, ^15^, ^18^ Unfortunately, these drugs have little if any curative potential, and in many cases, resistance develops during therapy. Therefore, several attempts have been made to detect novel targets of therapy and to develop new therapeutic strategies in MPN. One approach is to employ immunological targets expressed on the surface of MPN cells, including MPN‐initiating cells.^19^, ^20^, ^21^

The concept of neoplastic stem cells (NSC) has been developed with the aim to explain cellular hierarchies and related clonal architectures in various cancer types and to improve treatment strategies by eliminating disease‐initiating and ‐propagating cells.^22^, ^23^, ^24^, ^25^, ^26^, ^27^, ^28^, ^29^, ^30^ In fact, any form of therapy is only 'curative' when eliminating most or all NSC in a given patient. The disease‐propagating capability of NSC in hematopoietic neoplasms is usually demonstrated in highly immuno‐deficient mice, such as non‐obese diabetic severe combined immunodeficiency (NOD/SCID) mice lacking an IL‐2 receptor‐gamma chain, also known as NSG.

In most forms of AML, NSG mouse‐engrafting leukemic stem cells (LSC) reside in a CD34^+^ compartment of the malignant clone.^22^, ^23^, ^24^, ^30^ Depending on the AML variant and mouse strain employed, AML LSC reside in a CD34^+^/CD38^−^ and often also in a CD34^+^/CD38^+^ fraction.^31^ In the blast phase of chronic myeloid leukemia (CML), CD34^+^ LSC also exhibit CD38,^32^ while in chronic phase CML, LSC are primarily detectable in CD34^+^/CD38^−^ subpopulations.^25^, ^26^, ^33^, ^34^ Normal hematopoietic stem cells (HSC) also reside in a CD34^+^/CD38^−^ fraction of bone marrow (BM) cells.^22^, ^23^ In contrast to normal HSC, CML LSC exhibit CD25, CD26, and IL‐1RAP in an aberrant manner.^33^, ^34^, ^35^ Cell surface antigens frequently expressed at high levels on CD34^+^/CD38^−^ AML LSC include CD25, CD47, CD96, and CD371 (CLL‐1).^36^, ^37^, ^38^, ^39^, ^40^

So far, little is known about the phenotype and functional properties of disease‐initiating NSC in patients with MPN. A number of previous and more recent data suggest that immature myeloid cells in MPN display CD34.^41^, ^42^, ^43^, ^44^, ^45^, ^46^, ^47^ However, only a few attempts have been made to confirm functional stemness of these cells in a xenotransplantation model. A related problem is that it is notoriously difficult to engraft MPN cells in NSG mice. Recent data suggest that MPN cells may engraft better when these mice exhibit one or more human hematopoietic growth factors.^48^ Other studies have reported that immature CD34^+^ MPN cells display disease‐related mutations in *JAK2* or *CALR*, thereby confirming that these cells belong to the malignant clone.^44^ We have recently shown that putative CD34^+^/CD38^−^ MPN stem cells express pSTAT5.^46^ However, little is known about phenotypes and target expression profiles of NSC in ET, PV, and PMF.

The aims of the current study were to establish the phenotype and target expression profile of MPN‐propagating NSC and to compare NSC phenotypes in patients with untransformed MPN with target expression profiles of LSC in patients with sAML following MPN. In our xenotransplantation experiments, we used NSGS (NSG‐SGM3) mice expressing human interleukin‐3 (IL‐3), granulocyte‐macrophage colony‐stimulating factor (GM‐CSF), and stem cell factor (SCF).

## 2 PATIENTS AND METHODS

### 2.1 Reagents and antibodies

Reagents used in this study are described in the supplement. Fluorochrome‐conjugated monoclonal antibodies (mAb) applied to detect NSC and/or molecular targets in these cells are listed in Table S1.

### 2.2 Patients

A total of 111 patients with MPN (62 males, 49 females) and 12 with sAML following MPN (5 males, 7 females) were examined. MPN patients were classified according to WHO criteria.^9^, ^10^ The patients' characteristics are shown in Table S2. All patients gave written informed consent before BM or peripheral blood (PB) samples were obtained. The study was approved by the ethics committee of the Medical University of Vienna. Control BM cells were purchased from Lonza (Basel, Switzerland).

### 2.3 Culture of neoplastic cells and in vitro experiments

In drug incubation experiments primary BM or PB mononuclear cells (MNC), the *JAK2*V617F+ cell lines HEL and SET‐2, and the AML cell line UT‐7 were used. UT‐7 cells expressing various *CALR* mutants were established by CRISPR/Cas9 technology (clones del58/WT, del61/WT, and del61/del25) as reported.^49^ To test drug effects, cells were incubated in control medium or increasing concentrations of ruxolitinib, fedratinib, pelabresib, JQ1, dBET6, avapritinib, midostaurin, gemtuzumab ozogamicin (GO), or alemtuzumab at 37°C. After 48 h, proliferation was measured by determining ^3^H‐thymidine incorporation. Drug effects on cell viability or surface marker expression were measured by flow cytometry after 24 h. The cytotoxic effect of alemtuzumab was evaluated after 1 h of incubation in 30% complement‐containing human serum. In a separate set of experiments, MPN cells were incubated with interferon‐gamma (IFN‐G, 200 U/mL) and tumor necrosis factor‐alpha (TNF‐A, 200 ng/mL), alone or in combination, at 37°C for 24 h in the absence or presence of JAK2‐ or BRD4‐targeting drugs before expression of PD‐L1 was analyzed by flow cytometry. A detailed description is provided in the supplement.

### 2.4 Molecular studies

In all patients, primary MPN cells were routinely examined for the presence of *JAK2*, *CALR*, and *MPL* mutations as described in the supplement. The *JAK2*V617F allele burden was quantified in MPN cells and mouse BM cells by qPCR using Ipsogen's JAK2 MutantQuant (Qiagen, Valencia, CA, USA).

### 2.5 Flow cytometry and cell sorting

Phenotyping of CD34^+^/CD38^−^ stem cells and CD34^+^/CD38^+^ progenitor cells in BM and PB samples was performed by multicolor flow cytometry using combinations of fluorochrome‐conjugated mAb (Table S1) essentially as described.^34^, ^35^, ^46^, ^50^ The gating strategy employed to identify NSC (MPN) or LSC (sAML) is shown in Figure S1. Antibody‐staining results were controlled by isotype‐matched control mAb and were expressed as staining index (SI) defined as ratio of the median fluorescence intensities (MFI) obtained with CD‐specific mAb and isotype‐matched control mAb (MFI mAb divided by MFI control mAb) as reported.^34^, ^35^, ^46^, ^50^ Cell sorting was performed on a FACSAria Fusion sorter (BD Biosciences, San José, CA, USA) to purify subsets of MNC (CD34^+^, CD34^−^, CD38^+^, CD38^−^) or subsets of CD34^+^ cells (CD38^+^ versus CD38^−^ subsets) essentially as described.^34^, ^50^ Magnetic activated cell sorting was performed (prior to flow cytometry sorting) to deplete CD3^+^ T cells in MNC samples. Technical details are described in the supplement. The gating strategy employed to detect engraftment of human cells in the BM of NSGS mice is shown in Figure S2.

### 2.6 Xenotransplantation experiments

Primary MNC were T cell‐depleted and/or sorted to purify subfractions of MPN cells or sAML cells. MNC or purified cell fractions were injected into the tail vein of adult NSGS mice. In a separate set of experiments, MPN MNC were pre‐incubated with fedratinib (3 or 10 μM), GO (10 μg/mL), or fedratinib and GO, at 37°C for 1 h and then washed before injected into NSGS mice. After injection, mice were inspected daily and sacrificed as soon as they developed disease‐related symptoms or after a maximum observation time of 30 weeks. Details are described in the supplement. Animal studies were approved by the Ethics and Animal Welfare Committee of the University of Veterinary Medicine, Vienna in accordance with the University's guidelines for Good Scientific Practice and authorized by the Austrian Federal Ministry of Education, Science and Research (BMWFW‐68.205/0113‐WF/V/3b/2016, BMBWF‐68.205/0221‐V/3b/2019, and BMBWF‐2021‐0.236.115) in accordance with current legislation.

### 2.7 Statistical analysis

Statistical analyses applied in this study are described in the supplement. Differences were considered significant when *p* < .05.

## 3 RESULTS

### 3.1 NSGS‐engrafting MPN NSC reside in a CD34 ^+^/CD38 ^−^ cell fraction

**FIGURE 1:**
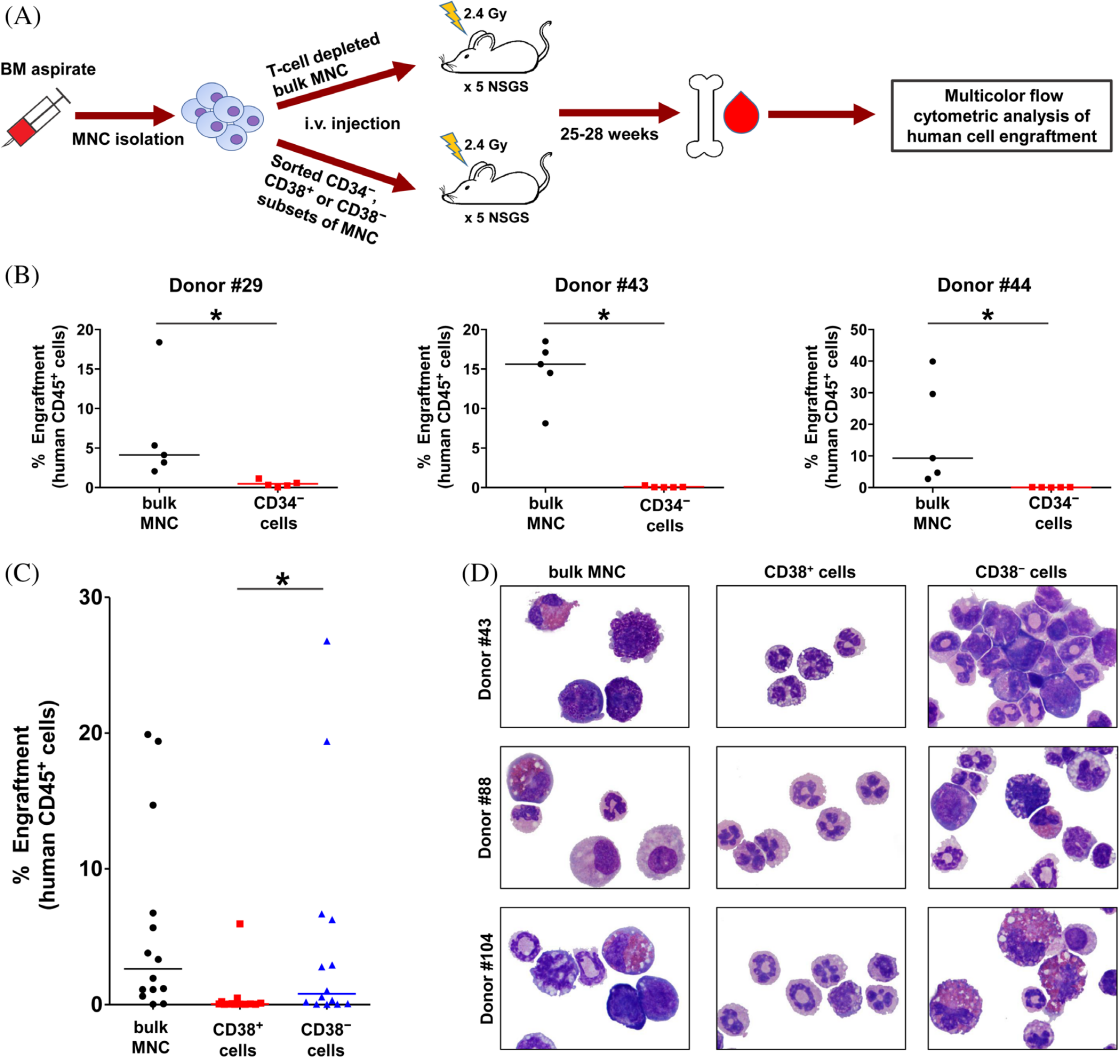
NSGS‐engrafting NSC preferentially reside in a CD34^+^/CD38^−^ MPN cell fraction. (A) Experimental design: BM derived MNC were T cell‐depleted and then sorted to obtain CD34^−^ cells (stem/progenitor cell‐depleted), CD38^+^ cells (progenitor enriched), and CD38^−^ cells (stem cell‐containing and progenitor‐depleted). Sorted cells were injected i.v. into sublethally irradiated NSGS mice. After 25–28 weeks, mice were sacrificed and engraftment of human cells in the BM of NSGS mice was evaluated by multicolor flow cytometry (Figure S2). (B) T cell‐depleted bulk MNC or purified CD34^−^ cells obtained from patients with ET (#29) or PV (#43 and #44) were i.v. injected into NSGS mice. After 28 weeks, mice were sacrificed and engraftment of human CD45^+^ cells (expressed as percentage of all BM cells) was evaluated by multicolor flow cytometry. Each dot represents the engraftment level in an individual mouse. The horizontal lines show the median of the percentage of engrafted human CD45^+^ cells. (C) Engraftment of human CD45^+^ cells from 3 MPN donors (#43, #88, and #104) in the BM of NSGS mice. T cell‐depleted bulk MNC or purified CD38^+^ and CD38^−^ cells were i.v. injected into irradiated NSGS mice. After 25 weeks, mice were sacrificed and engraftment of human CD45^+^ cells (expressed as percentage of all BM cells) was evaluated by multicolor flow cytometry. Each dot represents the engraftment level in an individual mouse. The horizontal lines show the median of the percentage of engrafted human CD45^+^ cells. Statistical significance of differences in engraftment rates in various cohorts of NSGS mice was calculated using Mann–Whitney *U*‐test (*p < .05). (D) Cytospin preparations of BM cells obtained from the injected mice were stained with Wright‐Giemsa and then examined under a light microscope (magnification, ×100). The figure shows engrafted human MPN cells (left and right columns) or lack of engraftment of human cells (only small murine neutrophilic granulocytes, middle column). Patient numbers (#) refer to Table S2. BM, bone marrow; ET, essential thrombocythemia; Gy, gray; i.v., intravenous; MNC, mononuclear cells; MPN, myeloproliferative neoplasm; NSC, neoplastic stem cells; PV, polycythemia vera. [Color figure can be viewed at wileyonlinelibrary.com]

To determine engraftment capabilities of MPN cells, different fractions of purified MPN cells were injected into NSGS mice. First, we applied bulk MNC and stem/progenitor cell‐depleted CD34^−^ MNC. After 28 weeks, engraftment of human CD45^+^ cells in the BM of NSGS mice was found in 15/15 mice injected with bulk MNC and in 0/15 mice injected with MNC depleted of CD34^+^ cells (Figure 1A,B; Table S3). In limiting dilution experiments, the calculated frequency of NSGS‐engrafting MPN NSC within the CD34^+^ fractions ranged between 0.3% and 1.3% (Figure S3; Table S4). We also compared engraftment of CD38^+^ and CD38^−^ subsets of MPN cells in 3 patients. Whereas the CD38^−^ cells produced engraftment in 10/14 mice, CD38^+^ cells engrafted in only 3/13 mice (Figure 1C; Table S3). Engrafted cells were found to harbor *JAK2*V617F (0.2%–97%) and consisted of basophils, eosinophils, mast cells, monocytes/macrophages, neutrophils, and blast cells (Figure 1D). Next, we applied sorted CD34^+^/CD38^−^ and CD34^+^/CD38^+^ MPN cells. In these experiments, only the CD34^+^/CD38^−^ cells produced engraftment (Figure S4A). By contrast, in sAML, both the CD34^+^/CD38^−^ and the CD34^+^/CD38^+^ cells engrafted in NSGS mice (Figure S4B,C).

### 3.2 Expression of cytokine‐ and chemokine receptors on MPN NSC

**FIGURE 2:**
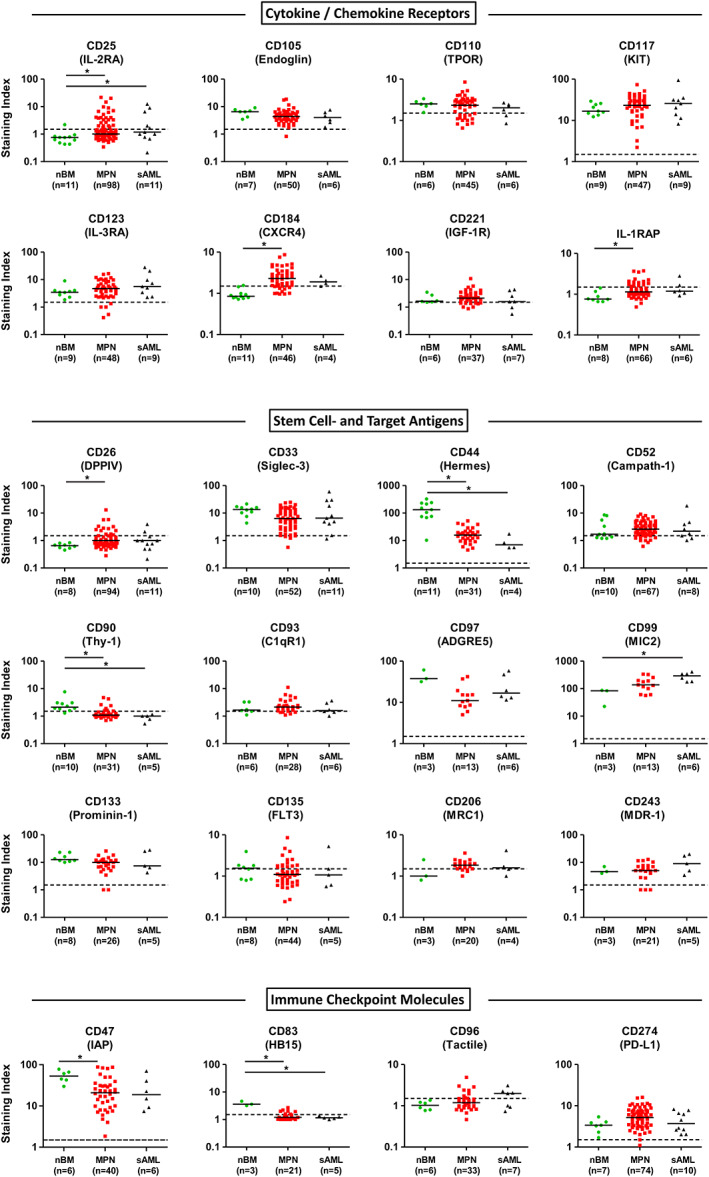
Expression of cell surface markers and targets on MPN NSC and sAML LSC. Samples from patients with MPN, sAML or healthy control samples were stained with fluorochrome‐labeled antibodies and stem cells were identified as CD34^+^/CD38^−^ cells (Figure S1). Expression of markers and targets on nBM HSC (green dots), MPN NSC (red squares) and post‐MPN sAML LSC (black triangles) was analyzed by multicolor flow cytometry. Results are expressed as staining index (median fluorescence intensity of the indicated marker divided by the median fluorescence intensity of the isotype control). Each symbol represents a single donor. Horizontal lines show the median expression level in each cohort. Dotted horizontal lines represent the cut‐off values for negativity (staining index <1.5). Significance levels of differences in expression of markers and targets on CD34^+^/CD38^−^ stem cells between healthy controls, MPN patients and post‐MPN sAML patients were analyzed by Kruskal–Wallis test followed by Dunn's multiple comparisons post hoc test (*p < .05). C1qR1, complement C1q receptor; CD, cluster of differentiation; CXCR4, chemokine C‐X‐C motif receptor 4; DPPIV, dipeptidyl peptidase IV; FLT3, FMS‐like tyrosine kinase 3; HSC, hematopoietic stem cells; IAP, integrin associated protein; IGF‐1R, insulin‐like growth factor 1 receptor; IL‐1RAP, interleukin‐1 receptor accessory protein; IL‐3RA, interleukin‐3 receptor alpha chain; LSC, leukemic stem cells; MDR‐1, multidrug resistance protein 1; MPN, myeloproliferative neoplasm; MRC1, mannose receptor C‐type 1; nBM, normal bone marrow; NSC, neoplastic stem cells; sAML, post‐MPN secondary acute myeloid leukemia; TPOR, thrombopoietin receptor. [Color figure can be viewed at wileyonlinelibrary.com]

We next established the phenotype of NSC in PV, ET, and myelofibrosis (MF), including PMF and post‐ET/PV MF. Independent of the type of MPN or the driver gene (*JAK2*V617F or *CALR*), CD34^+^/CD38^−^ NSC displayed the TGFB‐R‐related antigen endoglin (CD105), the SCF receptor KIT (CD117), and the IL‐3 receptor alpha chain (CD123) (Figure 2; Table S5). In about half of the patients tested, MPN NSC expressed higher levels of CD117 compared to normal HSC (Figure 2). Furthermore, CXCR4 (CD184) was expressed at higher levels on MPN NSC compared to normal HSC (Figure 2). In most patients with PV or ET, NSC did not express CD25 (IL‐2RA). By contrast, CD25 was expressed on NSC in about half of the patients with MF (Figure 2; Table S5). CD25 expression levels were slightly higher in patients with post‐ET/post‐PV MF compared to PMF patients (Table S6). In a subset of patients, NSC expressed the thrombopoietin receptor CD110, insulin‐like growth factor‐1 receptor (CD221) and/or IL‐1RAP (Figure 2). Except for a slightly elevated median CD25 level on NSC in MPN patients exhibiting *CALR* mutations and a slightly higher median KIT level on MPN NSC in *JAK2*V617F+ patients, no difference in the NSC phenotype was found when comparing *CALR*‐mutated MPN with *JAK2*V617F+ MPN (Figure S5). MPN NSC failed to express granulocyte colony‐stimulating factor receptor CD114, macrophage colony‐stimulating factor receptor CD115, the GM‐CSF/IL‐3/IL‐5 receptor beta chain CD131, nerve growth factor receptor CD271, vascular endothelial growth factor receptor CD309, MET, and oncostatin‐M receptor (Figure S6). MPN NSC also failed to express the erythropoietin receptor (EPOR) in both, *JAK2*V617F+ MPN and *CALR*‐mutated MPN. Compared to MPN NSC, the CD34^+^/CD38^+^ MPN progenitor cells displayed a similar profile of cytokine receptors (Figure S7; Table S7). In a subset of patients, MPN stem cells displayed higher levels of CD25 and CD221 compared to MPN progenitor cells (Figure S7). By contrast, the NSC in some patients displayed lower levels of IL‐1RAP compared to MPN progenitor cells (Figure S7).

### 3.3 Expression of stem cell antigens and molecular targets on MPN NSC

In all types of MPN, CD34^+^/CD38^−^ NSC expressed CD33, CD44, CD97, CD99, CD133, and the multidrug resistance antigen CD243 (Figure 2; Table S5). We observed a slightly elevated level of CD133 on NSC in MPN patients exhibiting *JAK2*V617F compared to MPN patients exhibiting *CALR* mutations (Figure S5). Unexpectedly, we found a negative correlation between the *JAK2*V617F allele burden and expression of CD97 and CD243 on NSC in our MPN patients (Figure S8). Another interesting finding was that in a subset of patients with MF, NSC displayed CD26 (Figure 2). In none of these patients, a *BCR::ABL1*+ subclone could be detected. MPN NSC expressed lower levels of CD44 and CD97 compared to HSC. In most MPN patients, we detected low levels of Campath‐1 (CD52). Regardless of the type of MPN, NSC did not exhibit substantial amounts of the stem cell antigens CD90, CD93, CD135, and CD206 (Figure 2; Table S5). CD34^+^/CD38^+^ MPN progenitors expressed a similar phenotype compared to NSC (Figure S7; Table S7). In most patients, these cells expressed higher levels of CD33 compared to NSC (Figure S7). In a subset of patients, MPN progenitors displayed lower levels of CD26, CD52, CD90, and CD133 compared to NSC (Figure S7).

### 3.4 Expression of immune checkpoint antigens on MPN NSC

In all MPN donors, NSC displayed the “don't eat me” receptor CD47 and the immune checkpoint antigen PD‐L1 (CD274) (Figure 2). In most patients, MPN NSC expressed higher levels of CD274 compared to HSC or LSC of sAML patients. In a few patients, MPN NSC expressed low amounts of CD83 and/or CD96. Other immune checkpoint antigens, including CD28, CD80, CD86, CD273, CD279, CD366 (TIM‐3), or CD371 (CLL‐1) were not detected on MPN NSC (Figure S6; Table S5). MPN progenitor cells and NSC displayed a comparable profile of checkpoint antigens (Figure S7; Table S7). In most patients, these cells expressed higher levels of CD371 (CLL‐1) compared to NSC (Figure S7). The phenotype of the *JAK2*V617F+ cell lines HEL and SET‐2 as well as UT‐7 cells (WT for *CALR* and *CALR*‐mutated) is shown in Table S8. These cells also expressed several targets and immune checkpoint antigens, including PD‐L1 (CD274).

### 3.5 The NSC phenotype does not change substantially during progression to sAML

In patients with sAML (post‐MPN), the putative LSC (CD34^+^/CD38^−^) expressed an almost identical phenotype compared to MPN NSC. In fact, LSC expressed CD33, CD44, CD47, CD52, CD97, CD99, CD105, CD117, CD123, CD133, CD184, CD243, and CD274 (Figure 2; Table S5). LSC expressed slightly higher levels of CD99 compared to MPN NSC and normal HSC. In a subset of patients, LSC displayed CD25, CD26, CD93, CD96, CD110, CD206, CD221, CD371, and IL‐1RAP. sAML LSC did not express CD28, CD80, CD83, CD86, CD90, CD114, CD115, CD131, CD271, CD273, CD279, CD309, CD366, or EPOR (Table S5). The cell surface antigen profile of CD34^+^/CD38^+^ sAML stem/progenitor cells did not differ substantially from the surface phenotype of CD34^+^/CD38^−^ sAML LSC (Figure S7; Table S7).

### 3.6 Effects of cytokines and targeted drugs on expression of PD‐L1 on MPN cells

**FIGURE 3:**
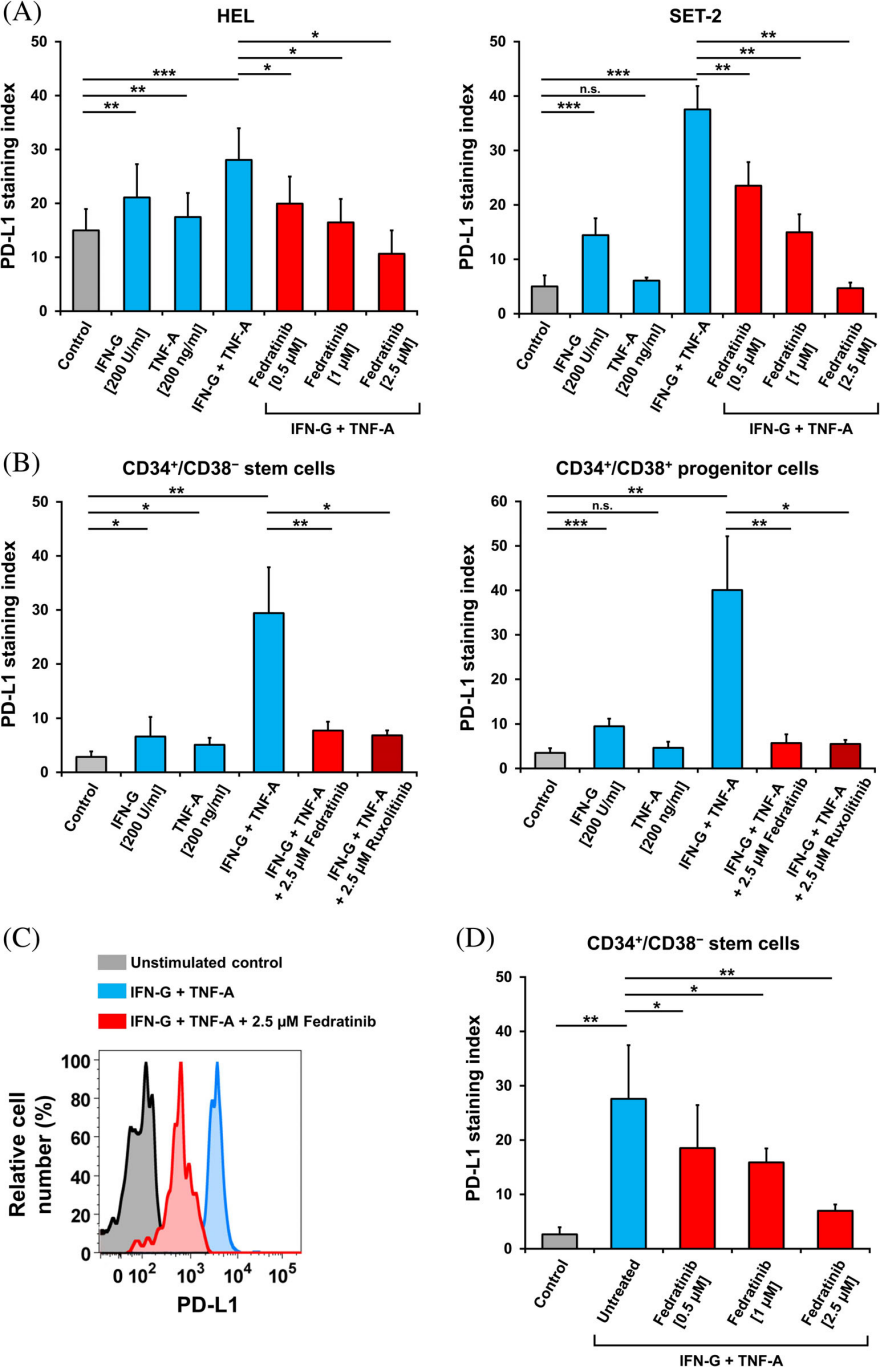
JAK2 inhibitors downregulate cytokine‐induced PD‐L1 expression on MPN cells. (A) *JAK2*V617F+ cell lines HEL and SET‐2 were incubated in control medium or medium containing either IFN‐G (200 U/mL), TNF‐A (200 ng/mL) or combination of both cytokines with or without fedratinib (0.5–2.5 μM) at 37°C for 24 h. Expression of PD‐L1 was determined by flow cytometry and presented as staining index (median fluorescence intensity of PD‐L1 divided by the median fluorescence intensity of the isotype control). Results represent the mean ± SD from at least three independent experiments. (B) Primary MNC from *JAK2*V617F+ MPN patients were incubated in control medium or medium containing either IFN‐G (200 U/mL), TNF‐A (200 ng/mL) or combination of both cytokines at 37°C and treated with fedratinib or ruxolitinib for 24 h. Expression of PD‐L1 levels on MPN CD34^+^/CD38^−^ stem cells and CD34^+^/CD38^+^ progenitor cells was evaluated by flow cytometry and shown as staining index. Results represent the mean ± SD from five *JAK2*V617F+ MPN patients. (C) Representative histograms, showing the cytokine‐induced expression of PD‐L1 on MPN CD34^+^/CD38^−^ stem cells (blue histogram) and the followed downregulation (red histogram) of PD‐L1 expression upon incubation with fedratinib. (D) Primary MNC from *JAK2*V617F+ MPN patients were incubated in control medium or medium containing combination of IFN‐G (200 U/mL) and TNF‐A (200 ng/mL) at 37°C and treated with fedratinib (0.5–2.5 μM) for 24 h. Expression of PD‐L1 levels on MPN CD34^+^/CD38^−^ stem cells was evaluated by flow cytometry and shown as staining index. Results represent the mean ± SD from three *JAK2*V617F+ MPN patients. Significance levels of differences in expression of PD‐L1 between the different conditions were analyzed by Student's *t*‐test (*p < .05; **p < .01; ***p < .001). IFN‐G, interferon‐gamma; MNC, mononuclear cells; MPN, myeloproliferative neoplasm; n.s., not significant; PD‐L1, programmed cell death ligand‐1; TNF‐A, tumor necrosis factor‐alpha. [Color figure can be viewed at wileyonlinelibrary.com]

We next asked whether cytokines modulate expression of immune checkpoint antigens on MPN cells. To address this question, MNC and MPN‐related cell lines were exposed to IFN‐G (200 U/mL), TNF‐A (200 ng/mL), or a combination of both cytokines. We found that both IFN‐G and TNF‐A promote the expression of PD‐L1 on MPN cells, with IFN‐G exerting more potent effects on PD‐L1 expression (Figure 3). A combination of both cytokines was found to strongly augment expression of PD‐L1 on these cells (Figure 3). The other checkpoint molecules tested (CD28, CD47, CD80, CD83, CD86, CD96, CD273, CD279, CD366, and CD371) did not change in expression after incubation with IFN‐G and/or TNF‐A (not shown). Next, we examined the effects of various drugs and signal‐transduction inhibitors on cytokine‐induced expression of PD‐L1. In these experiments, we found that the JAK2 inhibitors fedratinib (Figures 3 and S9) and ruxolitinib (Figures 3 and S10) suppressed IFN‐G‐ and TNF‐A‐induced upregulation of PD‐L1 in both *JAK2*V617F‐ and *CALR*‐mutated MPN cells. The BRD4‐targeting drugs JQ1 (Figure S11) and dBET6 (Figure S12) were also able to counteract the cytokine‐induced PD‐L1 expression in MPN cell lines and MPN NSC.

### 3.7 Effects of targeted drugs on expression of cytokine receptors and other target antigens on MPN NSC

**FIGURE 4:**
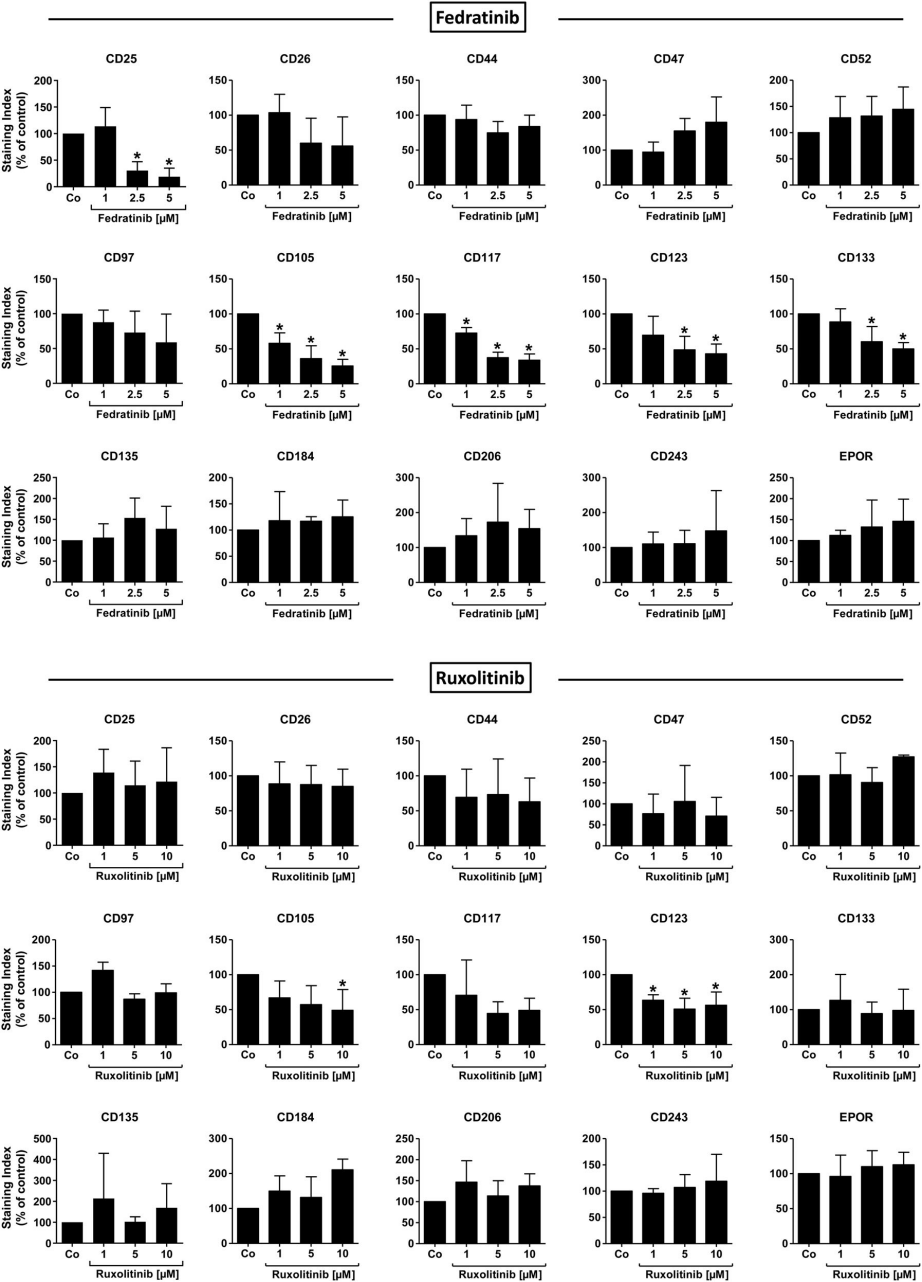
Effects of JAK2 inhibitors on expression of target antigens in MPN NSC. Primary MNC from *JAK2*V617F+ MPN patients were incubated in control medium (Co) or medium containing fedratinib (1–5 μM) or ruxolitinib (1–10 μM) at 37°C for 24 h. Expression of selected cell surface antigens (up‐ or down‐regulated on MPN stem cells) on CD34^+^/CD38^−^ stem cells were evaluated by multicolor flow cytometry and shown as staining index (% of control). Results represent the mean ± SD from up to six *JAK2*V617F+ MPN patients. Significance levels of differences in expression of surface antigens between the different conditions were analyzed by one‐way analysis of variance (ANOVA) with Dunnett's post hoc test (*p < .05 compared to control). EPOR, erythropoietin receptor; MNC, mononuclear cells; MPN, myeloproliferative neoplasm; NSC, neoplastic stem cells.

To evaluate whether surface antigens that were up‐ or down‐regulated on MPN NSC (compared to HSC) and/or correlated to a degree with the *JAK2*V617F allele burden are expressed on MPN cells in a *JAK2*‐dependent manner, MPN MNC were incubated in various concentrations of fedratinib (1–5 μM) or ruxolitinib (1–10 μM) for 24 h. Both JAK2 inhibitors suppressed the expression of CD105, CD117 and CD123 on MPN NSC in a dose‐dependent manner (Figure 4). By contrast, only fedratinib, but not ruxolitinib, was found to block expression of CD25 and CD133 on MPN NSC (Figure 4). Interestingly, fedratinib was also found to downregulate expression of CD105, CD117, CD123, and CD133 on normal HSC (not shown). The JAK2 inhibitors did not substantially up‐ or down‐regulate the expression of other target antigens, such as CD26, CD44, CD47, CD52, CD97, CD135, CD184, CD206, CD243, or EPOR on MPN NSC (Figure 4).

### 3.8 Effects of targeted drugs on growth and survival of MPN cells

To determine the effect of JAK2 inhibitors on proliferation and survival of MPN cells we incubated primary MPN MNC and MPN cell lines with ruxolitinib and fedratinib. Both JAK2 inhibitors suppressed the proliferation of MPN cells (Figure S13). Fedratinib also inhibited survival of primary CD34^+^/CD38^−^ MPN stem cells and MPN cell lines, whereas ruxolitinib did not induce substantial apoptosis in these cells (Figure S14). The KIT‐targeting drugs avapritinib and midostaurin were also tested. Both agents were found to inhibit proliferation in MPN cells (Figure S15). In addition, both drugs induced apoptosis in MPN cell lines, but did not induced apoptosis in primary MPN NSC (Figure S16). Among the BRD4‐targeting drugs tested, dBET6 showed superior effect over JQ1 and pelabresib at inhibiting proliferation of MPN‐related cell lines and of MPN NSC (Figure S17). Similarly, dBET6 was found to exert most potent apoptosis‐inducing effects in MPN cell lines and in primary CD34^+^/CD38^−^ MPN stem cells (Figure S18). Next, the effect of the CD33‐directed drug GO and CD52‐directed drug alemtuzumab were tested. In primary MPN MNC, GO suppressed proliferation in a dose‐dependent manner (Figure S19A). Moreover, GO was found to induce apoptosis in primary MPN NSC in our in vitro experiments, whereas GO failed to induce apoptosis in CD34^+^/CD38^+^ MPN progenitors (Figure S19B). Next, we tested the stem cell‐targeting effects of GO in our xenotransplantation model. When MPN MNC were incubated with GO 1 h prior to injecting into NSGS mice, the engraftment potential of NSC in these MNC was completely blocked as no engraftment of human CD45^+^ cells was detected in the BM of these mice (Figure S19C). Fedratinib was also tested in these experiments, but was unable to affect engraftment of MPN NSC, despite being effective in inducing in vitro apoptosis in these cells. Interestingly, despite expression of CD52, alemtuzumab was unable to induce cell lysis or apoptosis in vitro in MPN cells (not shown). An overview of the in vitro growth‐inhibitory effects of drugs on MPN cells is shown in Table S9 and a summary of apoptosis‐inducing drug effects in Table S10.

## 4 DISCUSSION

Although MPN are considered to be stem cell‐derived neoplasms, little is known about phenotypic and functional properties of MPN NSC.^46^, ^51^, ^52^ In this study, we show that MPN‐initiating NSC reside in a CD34^+^/CD38^−^ fraction of neoplastic cells and that these MPN NSC exhibit several cytokine receptors and various clinically relevant target antigens and checkpoint molecules, including CD33 and PD‐L1. The phenotypic characterization of MPN NSC should facilitate their enrichment, their molecular characterization, and the development of NSC‐eradicating, curative treatment concepts in MPN.

A number of different mouse models have been examined for their ability to serve as a tool for studying engraftment of MPN NSC.^41^, ^42^, ^43^, ^47^, ^48^, ^53^ In earlier studies, NSG mice were employed, but in these mice, only little if any engraftment was found. More recently, newborn MISTRG or NSG mice were found to provide engraftment in some MPN patients.^45^, ^47^, ^48^ However, in these models, the xenograft technique is difficult to standardize. NSGS mice have been used successfully to engraft stem cells in chronic myelomonocytic leukemia (CMML) with reproducible engraftment results.^54^, ^55^, ^56^ Since CMML may sometimes present as a myeloproliferative disorder (proliferative type of CMML), we employed NSGS mice in our studies. We found that these mice provide suitable engraftment results for MPN NSC although engraftment levels varied among donors, ranging between 0.1% and 40%.

To define the phenotype of MPN NSC, we examined the engraftment potential of purified (sorted) subfractions. First, we depleted the CD34^+^ cell fraction in MPN samples and found that these stem cell‐depleted fractions are unable to engraft NSGS mice whereas the bulk MNC (control cells) engrafted with clonal MPN cells. Next, we confirmed that the purified, sorted, CD34^+^ MPN cells can engraft in NSGS mice and determined their frequency in cell dilution experiments. The calculated frequency of MPN NSC among all injected CD34^+^ cells was found to be 0.3%–1.3%. Compared to other myeloid neoplasms examined by us and by others, this frequency of MPN NSC is rather high. For example, the frequency of NSGS‐engrafting LSC among CD34^+^ CMML cells ranged between 0.009% and 0.04%.^56^ One explanation for the relatively high percentage numbers of NSC in MPN could be that several different classes and subsets of stem cells develop in patients with MPN. Alternatively, NSGS mice are more permissive to MPN NSC than to CMML LSC or that MPN NSC are particularly responsive to the (human) myeloid growth factors expressed in these mice.^57^, ^58^ Finally, we were able to show that NSGS‐engrafting MPN cells preferentally reside in the CD34^+^/CD38^−^ subfraction of the clone, contrasting post‐MPN sAML, where both the CD34^+^/CD38^−^ and CD34^+^/CD38^+^ subfractions engrafted in NSGS mice.

In most experiments, the engrafted MPN cells were a mixture of myeloid cells, sometimes with an additional B cell compartment. In most cases, engrafting cells were basophils, eosinophils, mast cells, macrophages, neutrophils and blast cells. This multilineage pattern is best explained by the human cytokines expressed in NSGS mice. At the same time, transplanted NSC may have been recruited irreversibly into maturation pathways and thereby lost their stemness, which would explain why we did not see an engraftment in secondary recipient mice. We also confirmed the presence of *JAK2*V617F in engrafted MPN cells by qPCR. However, the variant allele frequency of *JAK2*V617F varied among donors and samples, and in several mice, only wild type *JAK2* could be detected. This may be explained by clonal selection of stem cell subsets expressing or lacking combinations of oncogenic networks and molecules, including *JAK2*V617F, which may often induce differentiation rather than proliferation.

So far, little is known about the phenotype of NSC in patients with MPN.^51^, ^52^ In the present study, we found that CD34^+^/CD38^−^ MPN NSC display numerous 'therapeutic' targets, including CD33, CD44, CD47, CD97, CD99, CD117, CD123, CD133, CD184, and CD274 (PD‐L1) independent of the type of MPN or molecular driver lesion. Some of these antigens, such as CD99, CD117, CD123, CD184, and CD274 were found to be expressed at higher levels on MPN NSC compared to HSC in most donors, confirming the neoplastic nature of cells. With regard to CD274 (PD‐L1) these data confirm our previous observations.^52^ Whether this excess in target expression would provide a therapeutic window remains unknown.

Some of these markers were variably expressed on NSC among patients depending on the MPN variant. For example, CD25 was detected on NSC in a majority of patients with MF, whereas in ET or PV, NSC usually lack CD25. In a few patients with MF, NSC also expressed CD26. Since CD26 is a specific marker for CML LSC and MPN patients may harbor small *BCR::ABL1*+ subclones, we examined neoplastic cells for expression of *BCR::ABL1*. However, *BCR::ABL1* was not detected in clonal cells in these cases. So far, the mechanisms underlying expression of CD26 in MPN NSC remain unknown.

With regard to CD25, our data suggest that the JAK2‐STAT5 pathway may be involved. In particular, fedratinib, a strong inhibitor of JAK2, suppressed CD25 expression in MPN cells. We also asked whether *JAK2*V617F and thus the JAK2‐STAT5 pathway also promotes the expression of other cell surface antigens in MPN NSC. In these experiments, we found that fedratinib downregulates expression of several surface antigens, including CD105, CD117, CD123, and CD133, and less effectively CD26 and CD97 in MPN NSC, suggesting that the JAK2‐STAT5 pathway is involved in expression of these antigens on MPN stem cells. Interestingly, ruxolitinib did not show comparable effects on expression of these antigens on MPN NSC, which is most probably due to the much weaker effects of this drug on *JAK2* and *JAK2*V617F compared to fedratinib. Another interesting observation was that although fedratinib induced downregulation of several surface antigens on MPN NSC, expression of these antigens on NSC did not correlate with the *JAK2*V617F allele burden. This observation may have several explanations. One would be that MPN NSC not only display *JAK2*V617F but also other (additional) molecular lesions (mutations) that promote or suppress expression of such surface molecules. Second, the *JAK2*V617F burden was determined in unfractionated leukocytes but not in CD34^+^/CD38^─^ stem cells.

Resistance of NSC is often associated with expression of certain checkpoint molecules, such as CD47, PD‐L1, or TIM‐3. In most hematopoietic malignancies, neoplastic cells express PD‐L1 after exposure to certain cytokines, like IFN‐G.^52^, ^59^, ^60^ We found that MPN NSC express PD‐L1 and CD47 in a constitutive manner. In the current study, we also show that IFN‐G upregulates PD‐L1 expression on MPN NSC and MPN‐related cell lines. In addition, we were able to show that TNF‐A augments IFN‐G‐induced upregulation of PD‐L1 on MPN cells, including NSC. In fact, maximum upregulation of PD‐L1 on NSC is only achieved by a cytokine storm when both cytokines, IFN‐G and TNF‐A, are present. Finally, our data show that the cytokine‐induced upregulation of PD‐L1 on MPN cells can be disrupted by addition of JAK2‐targeting agents and BET/BRD4‐targeting drugs. In fact, ruxolitinib and fedratinib were found to completely suppress cytokine‐induced upregulation of PD‐L1 in MPN NSC even when both, IFN‐G and TNF‐A, were applied. Similar effects, albeit less potent, were seen with the BRD4 degrader dBET6. These observations suggest that cytokine‐induced expression of PD‐L1 on MPN NSC depends on signaling pathway(s) involving JAK2 and BET‐related effector molecules like MYC, which confirms recently published results.^52^, ^59^, ^61^ Since PD‐L1 expression is considered to mediate stem cell resistance, knowledge about strategies to block PD‐L1 expression in MPN NSC may provide a suitable basis for the design of improved NSC‐targeting treatment strategies. We also asked whether the phenotype of MPN NSC changes during progression to sAML. Indeed, pro‐oncogenic alterations in the NSC compartment may play a role in transformation to sAML.^62^ However, in our study, most markers exhibited by MPN NSC were also found on sAML LSC, with comparable expression levels.

Because of resistance of MPN NSC, new drug therapies are currently being developed, including novel targeted drugs and immunotherapies. In the current study, we were able to show that MPN NSC express several clinically relevant surface targets, including CD33, CD44, CD47, CD52, CD117, CD123, CD184, and CD274. Since normal HSC also display these antigens, long‐term immunotherapy may still be too toxic because of HSC exhaustion. On the other hand, antibody‐based therapies, like treatment with GO may be a less toxic and thus a feasible approach. In order to test the hypothesis that CD33 may be a clinically relevant stem cell target, we exposed MPN NSC to GO. In these experiments, we found that GO inhibits proliferation and induces apoptosis in MPN NSC. Moreover, we were able to show that pre‐incubation of MPN NSC with GO completely disrupts their ability to engraft NSGS mice. We can of course not exclude that GO also inhibits engraftment of normal HSC in these experiments. An interesting observation was that the effects of GO in primary MPN cells appeared to be specific for NSC whereas no apoptosis‐inducing effect of this drug on MPN progenitor cells were found. Another interesting observation was that fedratinib, although effective in vitro in inducing apoptosis in MPN NSC, was not able to suppress the engraftment potential of MPN NSC in our NSGS mice. This unexpected observation is best explained by the fact that fedratinib is primarily acting on proliferating cells and is washed away after short term incubation whereas GO, once bound to NSC cannot be washed away and will enter NSC to induce cell death even after cells were injected into NSGS mice. It is also worth noting that the CD52‐targeting antibody alemtuzumab did not induce cell death in MPN cells in our experiments which may be due to the lower expression of CD52 on these cells. Indeed, expression levels of CD52 on LSC may correlate with responsiveness to alemtuzumab.^63^

Together, our data show that MPN‐initiating NSC can engraft NSGS mice and reside in a CD34^+^/CD38^−^ cell fraction. We also show that MPN NSC display a number of surface antigens, including cytokine receptors and therapeutic targets. The phenotypic characterization of MPN NSC should facilitate their enrichment and examination, and should support the development of NSC‐eradicating curative treatment concepts.

## AUTHOR CONTRIBUTIONS

Daniel Ivanov and Peter Valent designed the study, analyzed the data, and wrote the manuscript. Daniel Ivanov, Jelena D. Milosevic Feenstra, Irina Sadovnik, Harald Herrmann, Barbara Peter, and Gregor Eisenwort performed key laboratory experiments. Emir Hadzijusufovic, Sigrid Machherndl‐Spandl, Michael Fillitz, Thamer Sliwa, Maria‐Theresa Krauth, Peter Bettelheim, Wolfgang R. Sperr, Elisabeth Koller, Michael Pfeilstöcker, Heinz Gisslinger, and Felix Keil provided patients' samples and clinical information. Georg Greiner and Gregor Hoermann performed and analyzed molecular studies. Michael Willmann, Katharina Slavnitsch, Thomas Rülicke, and Maik Dahlhoff performed animal experiments. Robert Kralovics provided a vital cell line model and contributed to the study design. All authors wrote parts of the manuscript, corrected the draft version of the manuscript, and approved the final version of the document.

## CONFLICT OF INTEREST STATEMENT

P.V. received research grants from Celgene/BMS and AOP Orphan, and received consultancy honoraria from Novartis, Celgene/BMS, Pfizer, Incyte, and AOP Orphan. S.M.S. received travel support from Kite/Gilead and honoraria from Novartis, Jazz Pharmaceutics, Amgen, Celgene/BMS, Gilead, Servier, and AbbVie. M.F. received honoraria from Novartis, Amgen, Takeda, Celgene/BMS, Sanofi‐Aventis, Diagnosia, Sobi, MedAHEAD. T.S. received honoraria from Novartis, AbbVie, and Celgene/BMS. M.K. received honoraria from Amgen, Celgene/BMS, Janssen, Takeda, Pfizer, Sanofi, GSK, AbbVie, Novartis, and AOP Orphan. W.R.S. received honoraria from Novartis, Pfizer, AbbVie, Daiichi Sankyo, Stem line, Thermo Fisher, Deciphera, Celgene/BMS, and Jazz. E.K. received consultancy honoraria from AbbVie, Astellas, Jazz, Servier, and Celgene/BMS. M.P. received honoraria from Celgene/BMS, AbbVie, Jazz, Novartis, Sobi, and Takeda. The other authors declare no conflict of interest.

## INFORMED CONSENT

Informed consent was obtained in each case before cells were collected.

## Supporting information


**Data S1.** Supporting Information

## Data Availability

The data used and/or analyzed during the current study are available from the corresponding author on reasonable request.
