# Supplementary material for: Phenotypic characterization of disease‐initiating stem cells in JAK2‐ or CALR‐mutated myeloproliferative neoplasms
Source: Am J Hematol. 2023 Mar 9;98(5):770–83. doi: 10.1002/ajh.26889 (PMC10952374; doi:10.1002/ajh.26889)
Supplement: Supplementary file 1 — Data S1. Supporting Information [file AJH-98-770-s001.docx]

Supplementary Material to Ivanov et al.

**Phenotypic Characterization of Disease-Initiating Stem Cells in *JAK2*- or *CALR*-mutated Myeloproliferative Neoplasms**

Daniel Ivanov^1,2^, Jelena D. Milosevic Feenstra^2^, Irina Sadovnik^1,2^, Harald Herrmann^2,3^, Barbara Peter^1,2^, Michael Willmann^2,4^, Georg Greiner^2,5,6^, Katharina Slavnitsch^2,7^, Emir Hadzijusufovic^1,2,4^, Thomas Rülicke^2,8^, Maik Dahlhoff^2,7^, Gregor Hoermann^2,9^, Sigrid Machherndl-Spandl^10,11^, Gregor Eisenwort^1,2,12^, Michael Fillitz^12^, Thamer Sliwa^12^, Maria-Theresa Krauth^1,2^, Peter Bettelheim^10^, Wolfgang R. Sperr^1,2^, Elisabeth Koller^12^, Michael Pfeilstöcker^2,12^, Heinz Gisslinger^1^, Felix Keil^2,12^,

Robert Kralovics^5^, and Peter Valent^1,2^

^1^Department of Internal Medicine I, Division of Hematology and Hemostaseology, Medical University of Vienna, Austria; ^2^Ludwig Boltzmann Institute for Hematology and Oncology, Medical University of Vienna, Austria; ^3^Department of Radiation Oncology, Medical University of Vienna, Austria; ^4^Department for Companion Animals, Clinical Unit for Internal Medicine, University of Veterinary Medicine Vienna, Austria; ^5^Department of Laboratory Medicine, Medical University of Vienna, Austria; ^6^Ihr Labor, Medical Diagnostic Laboratories, Vienna, Austria; ^7^Institute of in vivo and in vitro Models, University of Veterinary Medicine Vienna, Austria; ^8^Department of Biomedical Sciences, University of Veterinary Medicine Vienna, Austria; ^9^MLL Munich Leukemia Laboratory, Munich, Germany; ^10^Hospital Ordensklinikum Elisabethinen Linz, Austria; ^11^Johannes Kepler University, Medical Faculty, Linz, Austria; ^12^Third Medical Department for Hematology

and Oncology, Hanusch Hospital, Vienna, Austria

**I. Supplementary Text**

## Reagents

RPMI 1640 medium, Iscove´s Modified Dulbecco´s Medium (IMDM), and penicillin/streptomycin were purchased from Lonza (Basel, Switzerland), amphotericin B from PAN-Biotech (Aidenbach, Germany), fetal calf serum (FCS) and phosphate-buffered saline (PBS) from Gibco (Gaithersburg, MD, USA), ruxolitinib, fedratinib, pelabresib, JQ1 and avapritinib from Selleckchem (Houston, TX, USA), gemtuzumab-ozogamicin (GO) from Pfizer (New York, NY, USA), midostaurin from LC Laboratories (Woburn, MA, USA), alemtuzumab from Genzyme (Cambridge, MA, USA), and dBET6 from Aobious (Gloucester, MA, USA). Stock solutions of drugs were prepared by dissolving in dimethyl sulfoxide (DMSO, Sigma Aldrich, St. Louis, MO, USA). ^3^H-thymidine was purchased from Perkin Elmer (Boston, MA, USA), 4´,6-diamidino-2-phenylindole (DAPI) from Sigma-Aldrich (St. Louis, MO, USA), Annexin-V (eBioscience, San Diego, CA, USA), trypan blue and recombinant human interferon-gamma (IFN-G) from Roche (Basel, Switzerland), and tumor necrosis factor alpha (TNF-A), and thrombopoietin (TPO) from PeproTech (Cranbury, NJ, USA). A specification of monoclonal antibodies (mAb) used in this study is provided in Table S1.

**Patients**

Patients were diagnosed at the Medical University of Vienna (Austria), the Elisabethinen Hospital Linz (Austria), or the Hanusch Hospital, Vienna (Austria). Diagnoses were established according to published criteria proposed in the classification of the World Health Organization (WHO).^1,2^ A total of 111 patients with myeloproliferative neoplasms (MPN) (62 males and 49 females) and 12 with secondary acute myeloid leukemia (sAML) following MPN (5 males and 7 females) were examined. The median age at diagnosis was 61 years (range: 20-96 years) in our patients with MPN, and 72 years (range: 55-88 years) in our patients with sAML. Patients with MPN suffered from essential thrombocythemia (ET, n=38), polycythemia vera (PV, n=24) and myelofibrosis (MF, n=49). Molecular studies were performed in all patients and revealed the presence of the *JAK2*V617F mutation in 80 patients with MPN, and of a *CALR* mutation in 28 patients with MPN.

The patients’ characteristics are shown in Table S2. Bone marrow (BM) or peripheral blood (PB) samples were collected during routine investigations at diagnosis and/or during follow up. BM was re-examined during follow up in the case of suspected progression. BM and PB samples were subjected to flow cytometry analysis and/or isolation of mononuclear cells (MNC) by density gradient centrifugation using Ficoll (Biochrom, Berlin, Germany). Isolated MNC were washed in PBS and either used immediately or were frozen in FCS containing 10% DMSO using a Mr. FrostyTM freezing-system (Thermo Fisher Scientific, Waltham, MA) and stored in a local biobank in liquid nitrogen until used. Frozen BM and PB cells were thawed in RPMI 1640 medium containing 10% FCS and antibiotics, followed by 30 minutes of incubation in RMPI 1640 medium with 10% FCS, antibiotics and 100 U/ml DNase type I (Sigma Aldrich, St. Louis, MO) to prevent cell clumping. After freeze-thawing, cell viability was examined by trypan blue exclusion and flow cytometry. Control BM MNC (normal BM, n=12 donors) were purchased from Lonza (Basel, Switzerland). All studies were approved by the ethics committees of the Medical University of Vienna (Austria), the Elisabethinen Hospital Linz (Austria), and the Hanusch Hospital Vienna (Austria) and conducted in accordance with the declaration of Helsinki. Informed consent was obtained in each case before cells were collected.

## Cell lines

The *JAK2*V617F+ cell lines HEL and SET-2 and the AML cell line UT-7 were purchased from the Leibniz-Institute DSMZ (Braunschweig, Germany). UT-7 cells engineered to express various *CALR* mutants by CRISPR/Cas9 technology (clones B5, C4 and E2) were established and maintained as reported.^3^ HEL and SET-2 cells were cultured in RPMI 1640 medium with 10% heat-inactivated FCS (HEL) or 20% heat-inactivated FCS (SET-2) and antibiotics at 37°C. UT-7 cells were maintained in IMDM with 10% heat-inactivated FCS and antibiotics at 37°C. UT-7 cells expressing wild type (WT) *CALR* were grown in human TPO (10 ng/ml), whereas the UT-7 subclones B5, C4 and E2 were TPO-independent and thus kept without TPO. All cells were periodically tested for expression of molecular markers, and for mycoplasma contamination by conventional PCR using the Venor GeM Classic Mycoplasma Detection Kit (Minerva Biolabs, Berlin, Germany).

**Culture of neoplastic cells and *in vitro* drug incubation experiments**

Patient-derived MNC and MPN-related cell lines were incubated in control medium or increasing concentrations of ruxolitinib (0.05-25 µM), fedratinib (0.05-25 µM), pelabresib (0.05-10 µM), JQ1 (0.05-10 µM), dBET6 (0.05-10 µM), avapritinib (0.05-25 µM), midostaurin (0.05-10 µM), alemtuzumab (10-500 µg/ml), or GO (0.01-25 µg/ml) at 37°C. After 48 hours, ^3^H-thymidine (0.5 µCi) was added and kept for another 16 hours at 37°C. Thereafter, cells were harvested on filter membranes and the bound radioactivity was measured in a beta-counter (Perkin Elmer, Boston, MA, USA). Viability and apoptosis of drug-exposed cells were determined by staining with Annexin-V and DAPI by multicolor flow cytometry as reported.^4–6^ Primary MNC were additionally stained with mAb against CD45, CD34 and CD38 to quantify apoptosis in putative neoplastic stem cells (NSC = CD45^dim^/CD34^+^/CD38^−^ cells) and progenitor cells (CD45^dim^/CD34^+^/CD38^+^ cells). In experiments employing alemtuzumab, cells were incubated in RPMI 1640 medium with 30% complement-containing human serum with or without alemtuzumab (10-500 µg/ml) at 37°C for 1 hour. Absolute numbers of viable cells were examined using CountBright beads (Invitrogen, Carlsbad, CA, USA) and DAPI-staining by flow cytometry. In a separate set of experiments, primary MNC from *JAK2*V617F+ MPN patients and MPN-related cell lines were incubated in control medium or medium containing either IFN-G (200 U/ml), TNF-A (200 ng/ml) or a combination of both cytokines, with or without various concentrations of JAK2 inhibitors (ruxolitinib or fedratinib) and BRD4-targeting drugs (JQ1 or dBET6) at 37°C. After 24 hours, expression of PD-L1 on cell lines or primary patient-derived cells (NSC and progenitors) was analyzed by multicolor flow cytometry on a FACSCanto II (BD Biosciences) using FlowJo software (TreeStar, Ashland, OR, USA). PD-L1 levels were expressed as staining index (SI) defined as ratio of the median fluorescence intensities (MFI) obtained with PD-L1-specific mAb and isotype-matched control mAb (MFI mAb : MFI control mAb) as reported.^4–7^

**Flow cytometry and cell sorting**

Phenotyping of CD45^dim^/CD34^+^/CD38^−^ stem cells and CD45^dim^/CD34^+^/CD38^+^ progenitor cells (in whole BM or PB) was performed by multicolor flow cytometry essentially as described^4–7^ using combinations of fluorochrome-conjugated mAb shown in Table S1. Heparinized BM or PB samples (25-100 µL per tube) were incubated in various combinations of mAb at room temperature (RT) for 15 minutes. Then, erythrocytes were lysed in FACS-Lysing Solution (BD Biosciences, San José, CA, USA) and expression of surface antigens on stem- and progenitor cells was analyzed by flow cytometry on a FACSCanto II (BD Biosciences) using FlowJo software (TreeStar, Ashland, OR, USA) as reported.^4–7^ The gating strategy employed to identify NSC (MPN) or LSC (sAML) is shown in Figure S1. Antibody-staining results were controlled by isotype-matched control mAb and were expressed as SI values which were graded using the following score: −, SI < 1.5; +/−, SI = 1.5-3; +, SI = 3.1-10; ++, SI = 10.1-100; +++, SI > 100. Cell sorting was performed on a FACSAria Fusion sorter (BD Biosciences) to purify subsets of MNC (CD34^+^, CD34^−^, CD38^+^, or CD38^−^ cells) or subsets of CD34^+^ cells (CD38^+^ versus CD38^−^ cells). Before FACS-sorting, magnetic activated cell sorting (MACS, Miltenyi Biotech, Bergisch Gladbach, Germany) was performed to deplete CD3^+^ T cells from MNC samples. After MACS and FACS-sorting, the purity of the sorted cell fractions was >95% in each case, and cell viability amounted to >80%.

## Xenotransplantation experiments

NOD.Cg-*Prkdc^scid^ Il2rg^tm1Wjl^* Tg(CMV-IL3,CSF2,KITLG)1Eav/MloySzJ mice expressing human IL-3, GM-CSF and SCF on a NSG background (NSGS mice) were purchased from Jackson Laboratory (Bar Harbor, ME, USA). NSGS mice were kept in specific opportunistic pathogen free quality (SOPF) under stringent controlled conditions. Twenty-four hours prior to injection, mice were sublethally irradiated (2.4 Gy). In typical experiments, sorted CD34^+^ MPN cells (or sub-fractions) were re-suspended in RPMI 1640 medium with antibiotics plus 10% FCS and injected into the lateral tail vein of adult (8-12 weeks old) NSGS mice of both sexes (0.2-0.6 x 10^6^ MPN cells/mouse; 5 NSGS mice per group). CD3-depleted MNC, prepared by MACS, served as control. In dilution experiments, increasing cell numbers of CD34^+^ MNC (50, 500, and 5,000 cells/mouse) were injected into NSGS mice. After injection, mice were inspected daily and sacrificed as soon as they developed disease-related symptoms or after a maximum observation time of 30 weeks. Then, long bones (femora, tibiae, humeri) were flushed to recover BM cells for flow cytometry and mRNA analyses. Multicolor flow cytometry was performed using mAb against CD19, CD33 and CD45 (Figure S2). Engraftment was defined as a population of at least 0.1% human CD45^+^ cells in flushed mouse BM samples. Engraftment was classified as myeloid (CD33^+^/CD19^−^ cells), lymphatic (CD33^−^/CD19^+^ cells) or mixed engraftment. In most experiments, a predominant myeloid engraftment (CD33^+^ cells) was found. In a separate set of experiments, MPN MNC were pre-incubated with fedratinib (3 or 10 µM) and/or GO (10 µg/ml) at 37°C for 1 hour prior to injection. The cells were washed with PBS before injection into NSGS mice. Engrafted MPN cells were also examined on cytospin slides after Giemsa staining to study morphologic features of these cells, including the maturation stage and cell lineage. Animal studies have been approved by the Ethics and Animal Welfare Committee of the University of Veterinary Medicine, Vienna in accordance with the University's guidelines for Good Scientific Practice and authorized by the Austrian Federal Ministry of Education, Science and Research (BMWFW-68.205/0113-WF/V/3b/2016, BMBWF-68.205/0221-V/3b/2019 and BMBWF-2021-0.236.115) in accordance with current legislation.

**Molecular studies**

Molecular studies were performed on BM and/or PB cells during routine investigations in all patients with MPN or post-MPN sAML at diagnosis and during follow up. Isolated cells were examined for expression of *JAK2*V617F and other disease-related mutations within *MPL* exon 10 or *CALR* mutations in exon 9. Genomic DNA was extracted using QIAamp DNA mini kit (Qiagen, Valencia, CA). DNA concentrations as well as the ratio of absorbance at 260 and 280 nm (A260/280) for assessment of DNA purity were measured in a NanoDrop 2000c spectrophotometer (Thermo Fisher Scientific, Waltham, MA). For quantification of *JAK2*V617F mutant alleles and *JAK2* wild type alleles, Ipsogen’s JAK2 MutantQuant Kit (Qiagen, Valencia, CA) was used and real-time-PCR was performed on Light Cycler 480II (Roche Applied Sciences, Indianapolis, IN). The *JAK2*V617F allele burden was calculated as follows: *JAK2*V617F in % = *JAK2*V617F copy numbers: (*JAK2*V617F copy numbers + *JAK2* wild type copy numbers) x 100. For detection of *MPL*W515K and *MPL*W515L mutations, Ipsogen’s MPL W515L/K Mutascreen Kit (Qiagen, Valencia, CA) was used and real-time-PCR was performed on Light Cycler 480II (Roche Applied Sciences, Indianapolis, IN). *CALR* mutations in MPN patients were detected by fluorescence-based detection of PCR products separated by capillary electrophoresis using ABI genetic analyzer 3500xl (Thermo Fisher Scientific, Waltham, MA). Analysis of the outcome was performed by GeneMapper software (Thermo Fisher Scientific, Waltham, MA). To determine the type of *CALR* mutations, PCR fragments were Sanger-sequenced using the BigDye Terminator version 3.1 cycle-sequencing kit (Life Technologies) and analyzed on ABI genetic analyzer 3500xl (Thermo Fisher Scientific, Waltham, MA).

**Statistical analysis**

Statistical calculations and analyses were performed using GraphPad Prism 9 software (San Diego, California, USA). Statistical significance of differences in engraftment rates in various cohorts of NSGS mice was calculated using Mann-Whitney U-test. The frequency of LSC among the CD34^+^ cells was calculated in xenotransplantation assays using extreme limiting dilution analysis.^8^ Significance levels of differences in expression of markers and targets on stem- and progenitor cells between healthy controls, MPN patients and post-MPN sAML patients were analyzed by Kruskal–Wallis test followed by Dunn’s multiple comparisons post hoc test. Significance levels of differences in expression of markers and targets between stem- and progenitor cells in various groups of patients were analyzed by Mann-Whitney U-test. Spearman's rank correlation coefficients were calculated to determine correlations between expression levels of cell surface markers on NSC and *JAK2*V617F allele burden in patients with MPN. To determine the significance of differences in expression of markers seen between drug-exposed and untreated MPN NSC, the one-way analysis of variance (ANOVA) with Dunnett’s post hoc test was applied. To determine the significance in differences seen between cytokine stimulated or drug exposed and unstimulated/untreated cells, Student’s t test was applied. Differences were considered significant when p<0.05.

**II. Supplementary Figures**

**Figure S1**


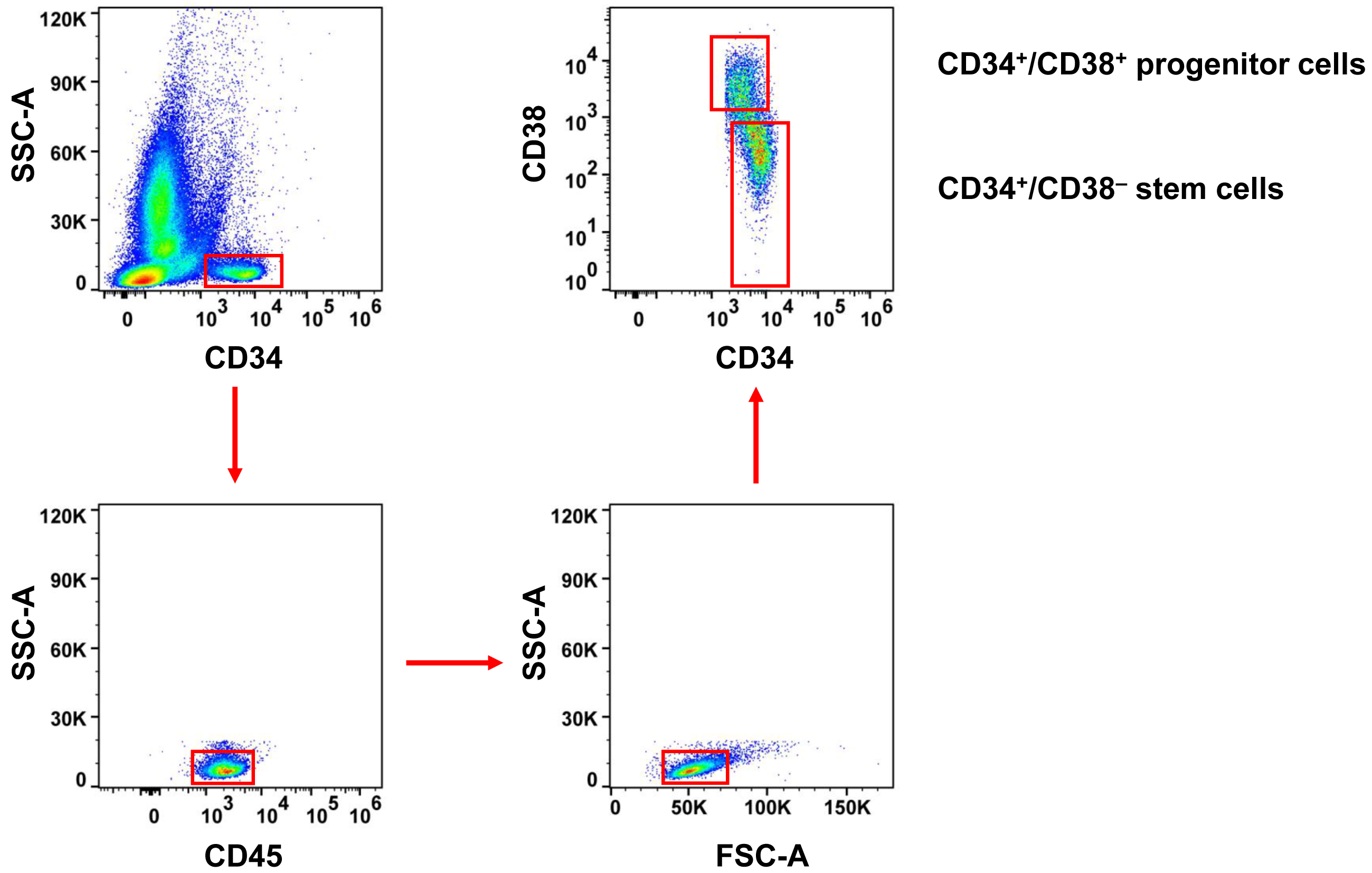


**Gating strategy for the identification of stem- and progenitor cells in primary MPN samples**

Primary bone marrow (BM) cells were obtained from a patient with post-MPN sAML (#115, Table S2). In this patient, the percentage of BM blasts was 7% and the percentage of blasts in the peripheral blood amounted to 25%. The sequential gating strategy is indicated by red arrows. After identifying stem- and progenitor cells by expression of CD34 (upper left panel), these cells were gated as CD45 dim-positive cells (lower left panel). Thereafter, viable cells were selected by their SSC-A and FSC-A properties (lower right panel) and were then gated according to their expression or lack of CD38 (upper right panel). Progenitors were identified as CD34^+^/CD38^+^ cells and stem cells as CD34^+^/CD38^−^ cells. Abbreviations: FSC-A, forward scatter area; MPN, myeloproliferative neoplasm; sAML, secondary acute myeloid leukemia following MPN; SSC-A, side scatter area.

**Figure S2**


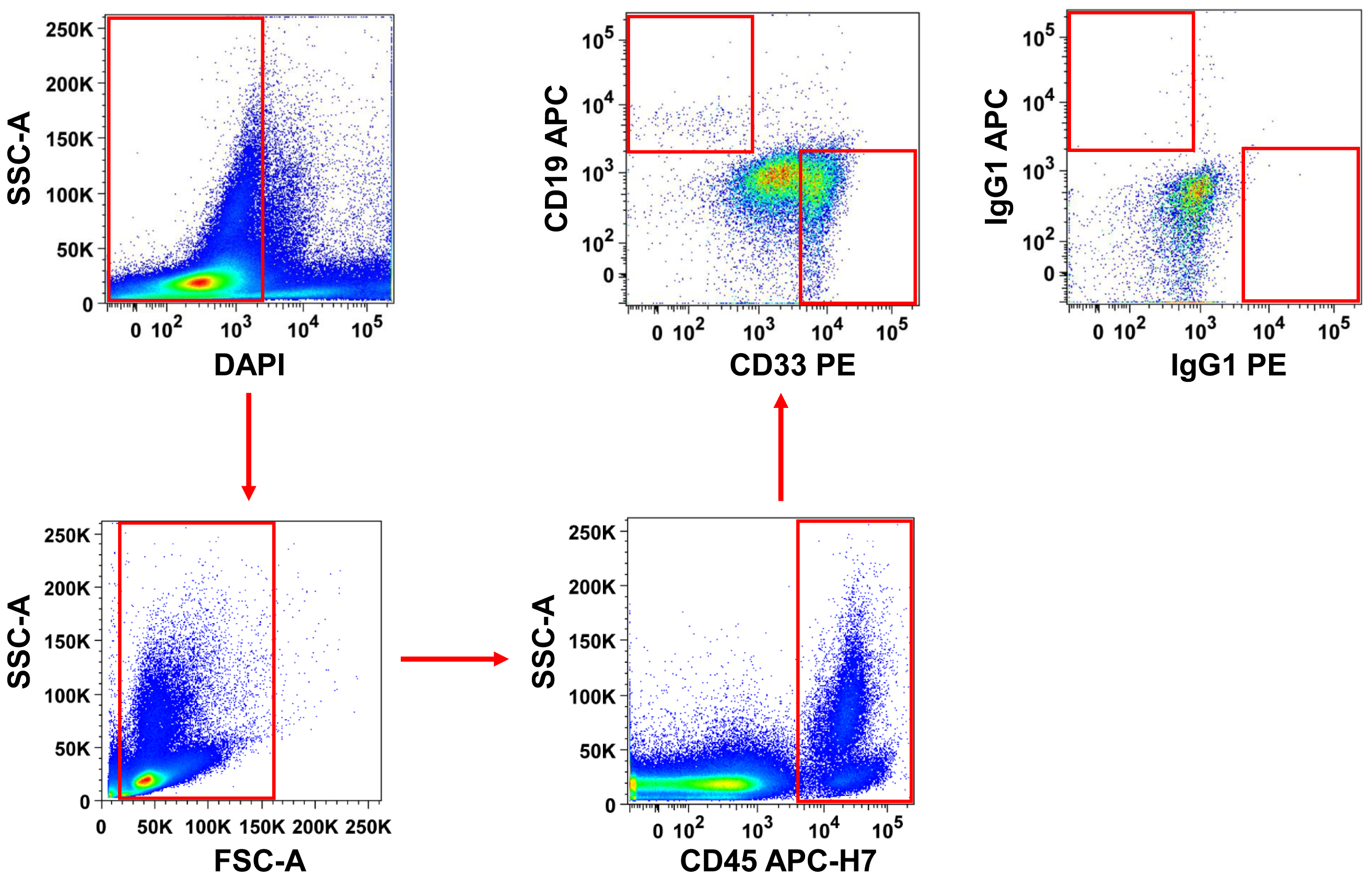


**Gating strategy for the identification of engrafted human cells in NSGS mouse bone marrow samples**

Purified CD34^+^ BM cells obtained from a patient with primary myelofibrosis (#88, Table S2) were intravenously injected into sublethally irradiated NSGS mice. After 28 weeks, mice were sacrificed and engraftment of human cells in the BM was evaluated by multicolor flow cytometry. The sequential gating strategy is indicated by red arrows. After excluding non-viable cells (upper and lower left panel), engrafted human CD45^+^ cells (lower right panel) were further evaluated for the type of engraftment (myeloid, lymphoid or mixed) based on expression of CD33 (myeloid engraftment) and/or CD19 (lymphoid engraftment) on engrafted human (neoplastic) cells. Staining reactivity produced by an isotype control antibody (IgG1) is also shown (upper right panel). Abbreviations: APC, allophycocyanin; BM, bone marrow; DAPI, 4′,6-diamidino-2-phenylindole; FSC-A, forward scatter area; PE, phycoerythrin; SSC-A, side scatter area.

**Figure S3**


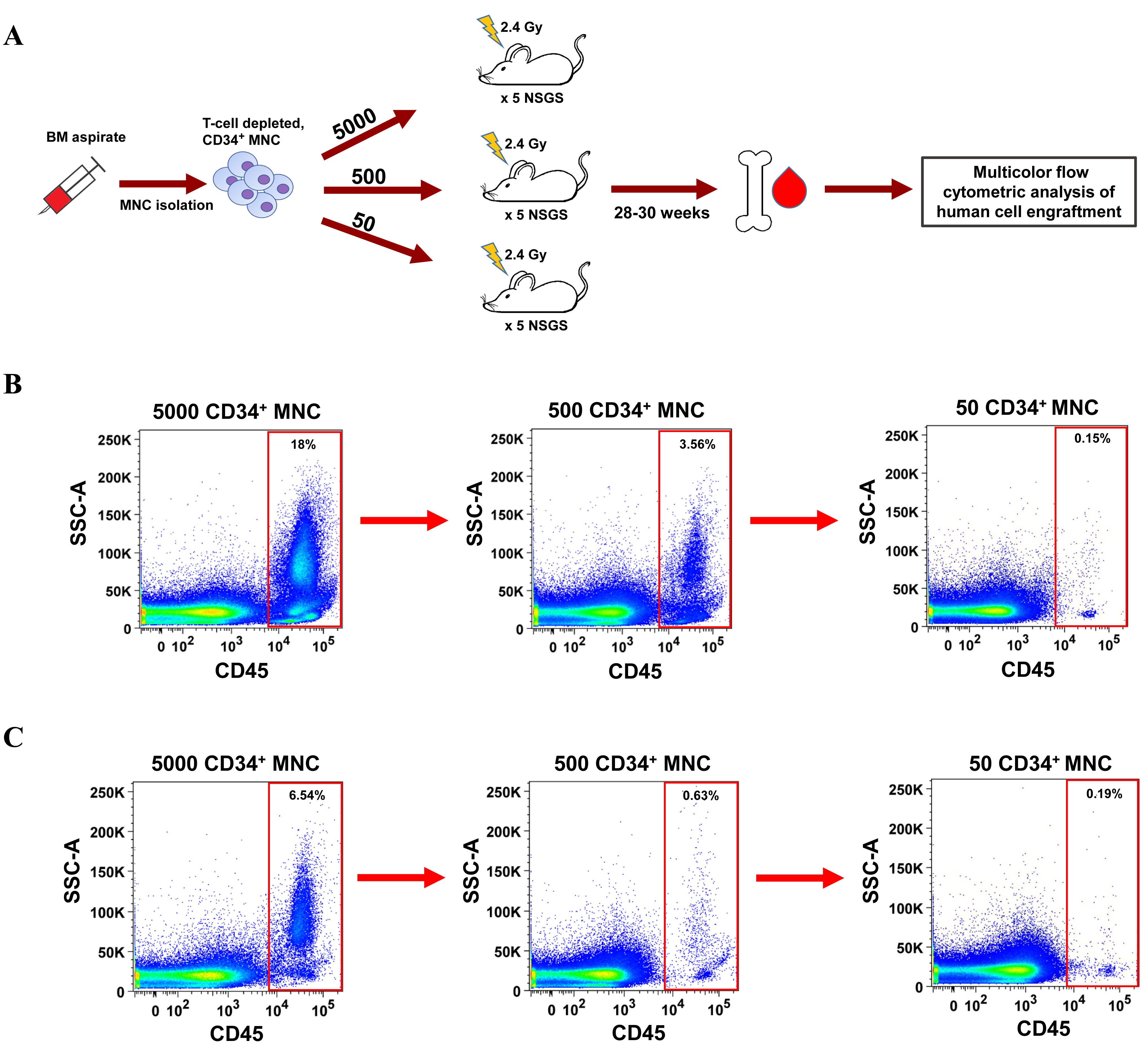


**Cell dilution experiments to determine the frequency of NSGS-engrafting NSC**

(A) Schematic illustration of the cell dilution experiments to define the numbers (frequency) of engrafting NSC. A total number of 50, 500 or 5000 purified CD34^+^ BM cells obtained from patients with MPN were intravenously injected into sublethally irradiated NSGS mice. After 28-30 weeks, mice were sacrificed and engraftment of the BM cells was evaluated by multicolor flow cytometry. (B) and (C) Representative examples of human CD45^+^ cell engraftment (MNC from patient #43 and #88 respectively) in the BM of NSGS mice, as determined by multicolor flow cytometry. Arrows indicate the gradually decreasing degree of engraftment, when smaller numbers of CD34^+^ cells were injected. Patient numbers (#) refer to Table S2. Abbreviations: BM, bone marrow; Gy, gray; MNC, mononuclear cells; MPN, myeloproliferative neoplasm; NSC, neoplastic stem cells; SSC-A, side scatter area.

**Figure S4**


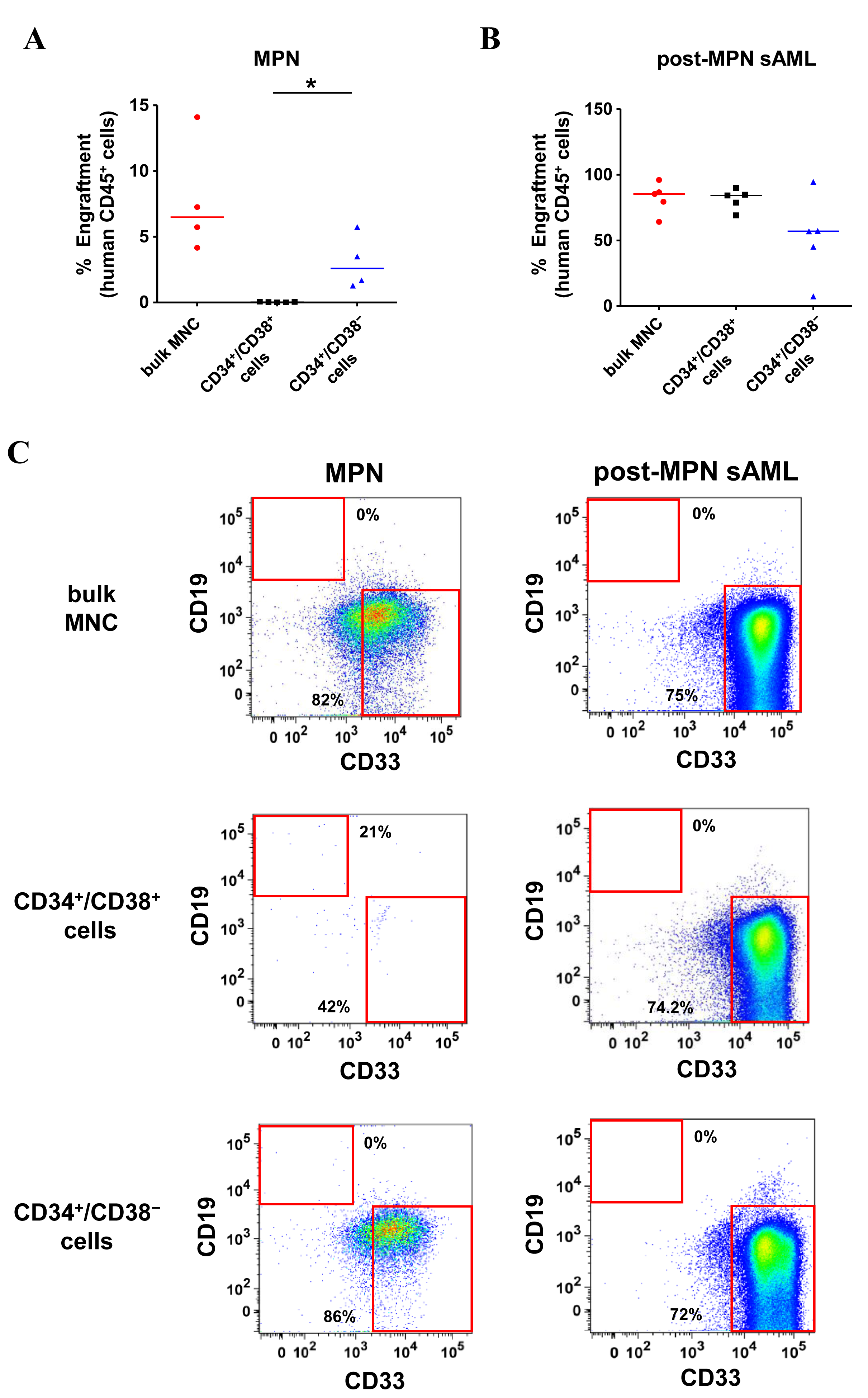


**CD34^+^/CD38^−^ MPN MNC engraft in NSGS mice**

MNC were obtained from a patient with PMF (A) (#104) and a patient with post-PV sAML (B) (#114). After T cell depletion, the following cell fractions were injected intravenously into sublethally irradiated NSGS mice: bulk MNC, sorted CD34^+^/CD38^+^ MNC (progenitors), and sorted CD34^+^/CD38^−^ MNC (stem cells). After 28 weeks (A) or 8 weeks (B), mice were sacrificed and the engraftment of human CD45^+^ cells (expressed as percentage of all flushed BM cells) was analyzed by multicolor flow cytometry (each symbol represents engraftment in an individual mouse). The horizontal lines show the median percentage levels of engrafted human CD45^+^ cells. Statistical significance of differences in engraftment rates in various cohorts of NSGS mice was calculated using Mann-Whitney U-test (*, p<0.05). (C) Representative dot plots (examples) showing the levels of engraftment with human CD45^+^/CD33^+^ myeloid cells and CD45^+^/CD19^+^ B-cells in the BM of NSGS mice from each cohort presented in A or B, including MPN cell fractions (#104, left panels) and sAML cell fractions (#114, right panels). Patient numbers (#) refer to Table S2. Abbreviations: MNC, mononuclear cells; MPN, myeloproliferative neoplasm; PMF, primary myelofibrosis; PV, polycythemia vera; sAML, secondary acute myeloid leukemia following MPN.

**
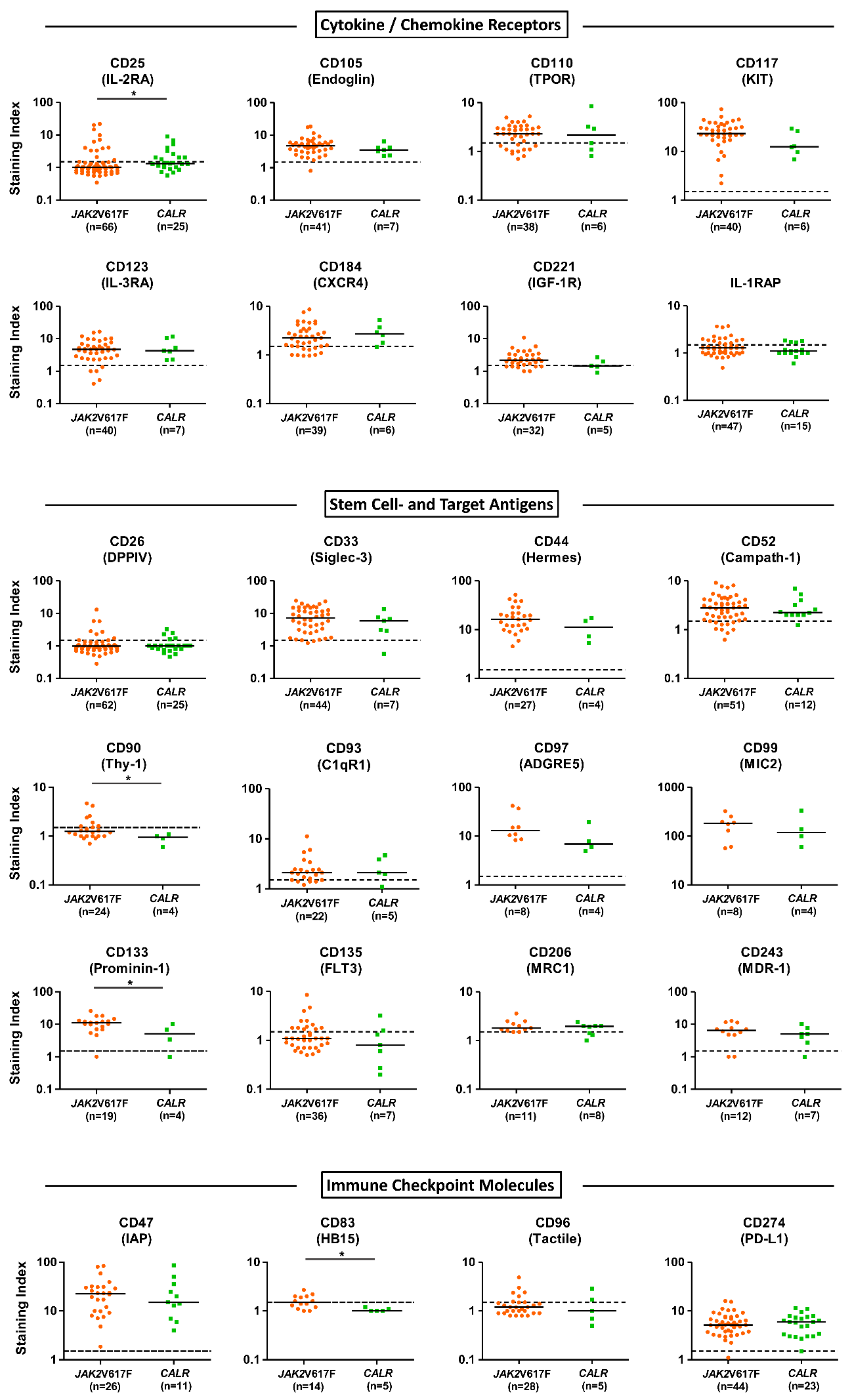
Figure S5**

**Expression of cell surface markers and targets on NSC in patients with MPN exhibiting either *JAK2*V617F or *CALR* mutation**

Samples from patients with MPN were stained with fluorochrome-labeled antibodies and stem cells were identified as CD34^+^/CD38^−^ cells (Figure S1). Expression of markers on MPN NSC harboring *JAK2*V617F mutation (orange dots) or a *CALR* mutation (green squares) was analyzed by multicolor flow cytometry. Results are expressed as staining index (median fluorescence intensity of the indicated marker divided by the median fluorescence intensity of the isotype control). Each symbol represents a single donor. Horizontal lines show the median expression level in each cohort. Dotted horizontal lines represent the cut-off values for negativity (staining index <1.5). Significance levels of differences in expression of markers and targets on NSC between the two groups of MPN patients were analyzed by Mann-Whitney U-test (*, p<0.05). Abbreviations: C1qR1, complement C1q receptor; CD, cluster of differentiation; CXCR4, chemokine C-X-C motif receptor 4; DPPIV, dipeptidyl peptidase IV; FLT3, FMS-like tyrosine kinase 3; IAP, integrin associated protein; IGF-1R, insulin-like growth factor 1 receptor; IL-1RAP, interleukin-1 receptor accessory protein; IL-2RA, interleukin-2 receptor alpha chain; IL-3RA, interleukin-3 receptor alpha chain; MDR-1, multidrug resistance protein 1; MPN, myeloproliferative neoplasm; MRC1, mannose receptor C-type 1; NSC, neoplastic stem cells; PD-L1, programmed death ligand 1; TPOR, thrombopoietin receptor.

**
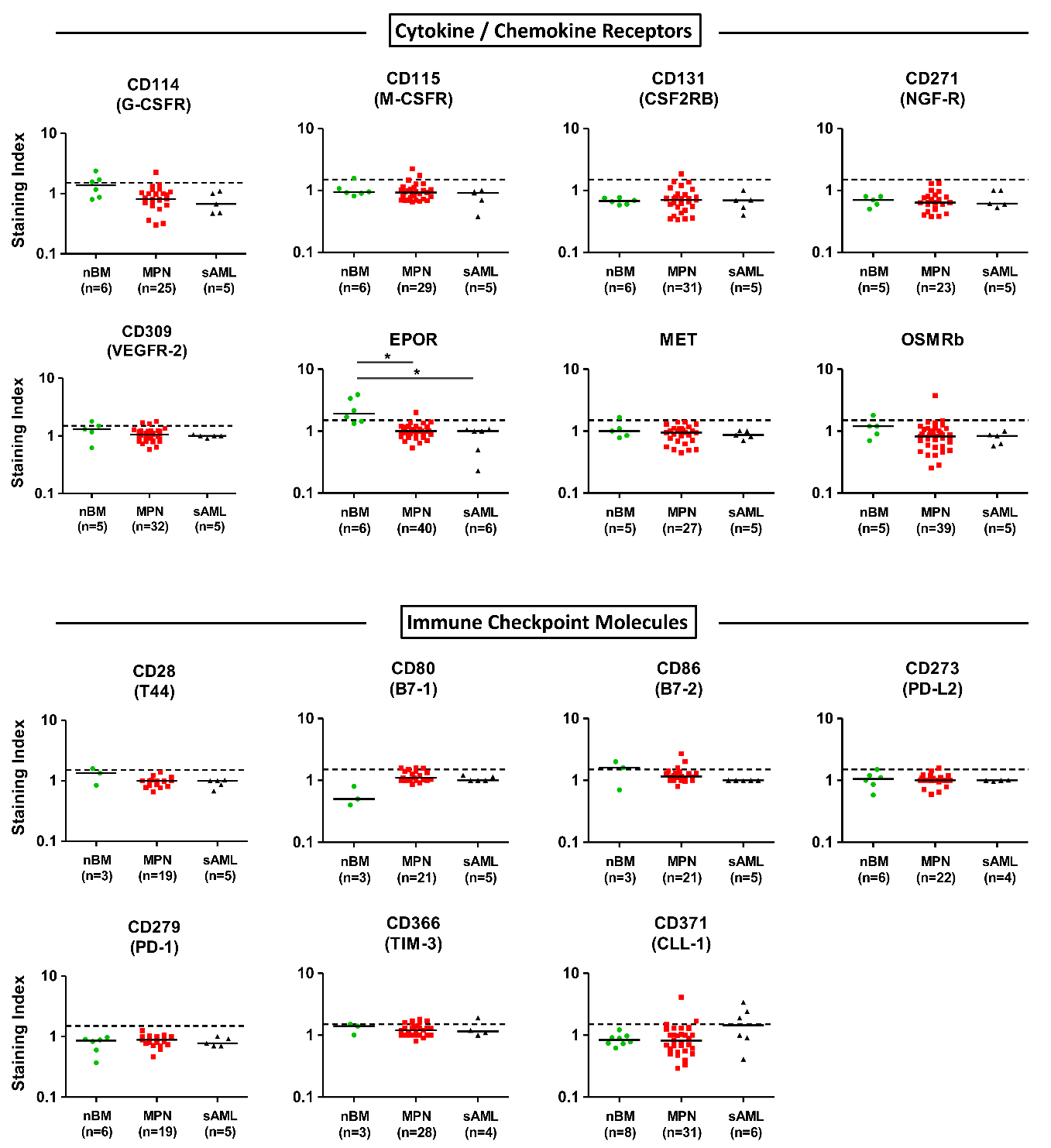
** **Figure S6**

**Evaluation of expression of cytokine receptors and checkpoint antigens on MPN NSC and sAML LSC**

Samples from patients with MPN, sAML or healthy control samples were stained with fluorochrome-labeled antibodies and stem cells were identified as CD34^+^/CD38^−^ cells (Figure S1). Expression of markers and targets on nBM HSC (green dots), MPN NSC (red squares) and post-MPN sAML LSC (black triangles) was analyzed by multicolor flow cytometry. Results are expressed as staining index (median fluorescence intensity of the indicated marker divided by the median fluorescence intensity of the isotype control). Each symbol represents a single donor. Horizontal lines show the median expression level in each cohort. Dotted horizontal lines represent the cut-off values for negativity (staining index <1.5). Significance levels of differences in expression of markers and targets on CD34^+^/CD38^−^ cells between healthy controls, MPN patients and post-MPN sAML patients were analyzed by Kruskal–Wallis test followed by Dunn’s multiple comparisons post hoc test (*, p<0.05). Abbreviations: CD, cluster of differentiation; CLL-1, C-type lectin-like molecule-1; CSF2RB, colony-stimulating factor 2 receptor-beta; EPOR, erythropoietin receptor; G-CSFR, granulocyte colony-stimulating factor receptor; HSC, hematopoietic stem cells; LSC, leukemic stem cells; M-CSFR, macrophage colony-stimulating factor receptor; MPN, myeloproliferative neoplasm; nBM, normal bone marrow; NGF-R, nerve growth factor receptor; NSC, neoplastic stem cells; OSMRb, oncostatin M receptor beta; sAML, secondary acute myeloid leukemia following MPN; PD-1, programmed death protein 1; PD-L2, programmed death ligand 2; TIM-3, T cell immunoglobulin and mucin domain-containing protein 3; VEGFR-2, vascular endothelial growth factor receptor 2.

**
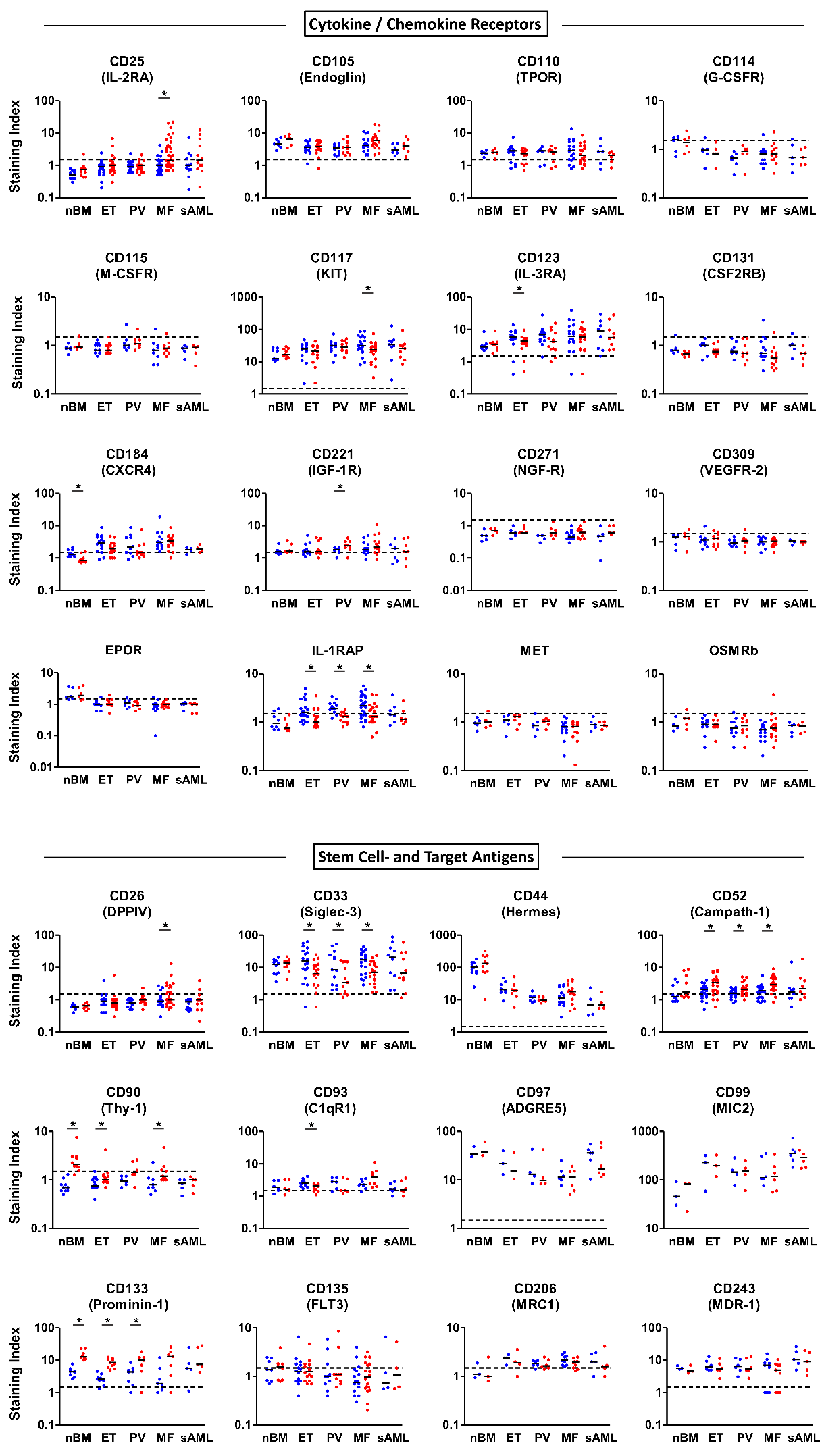
Figure S7**

**
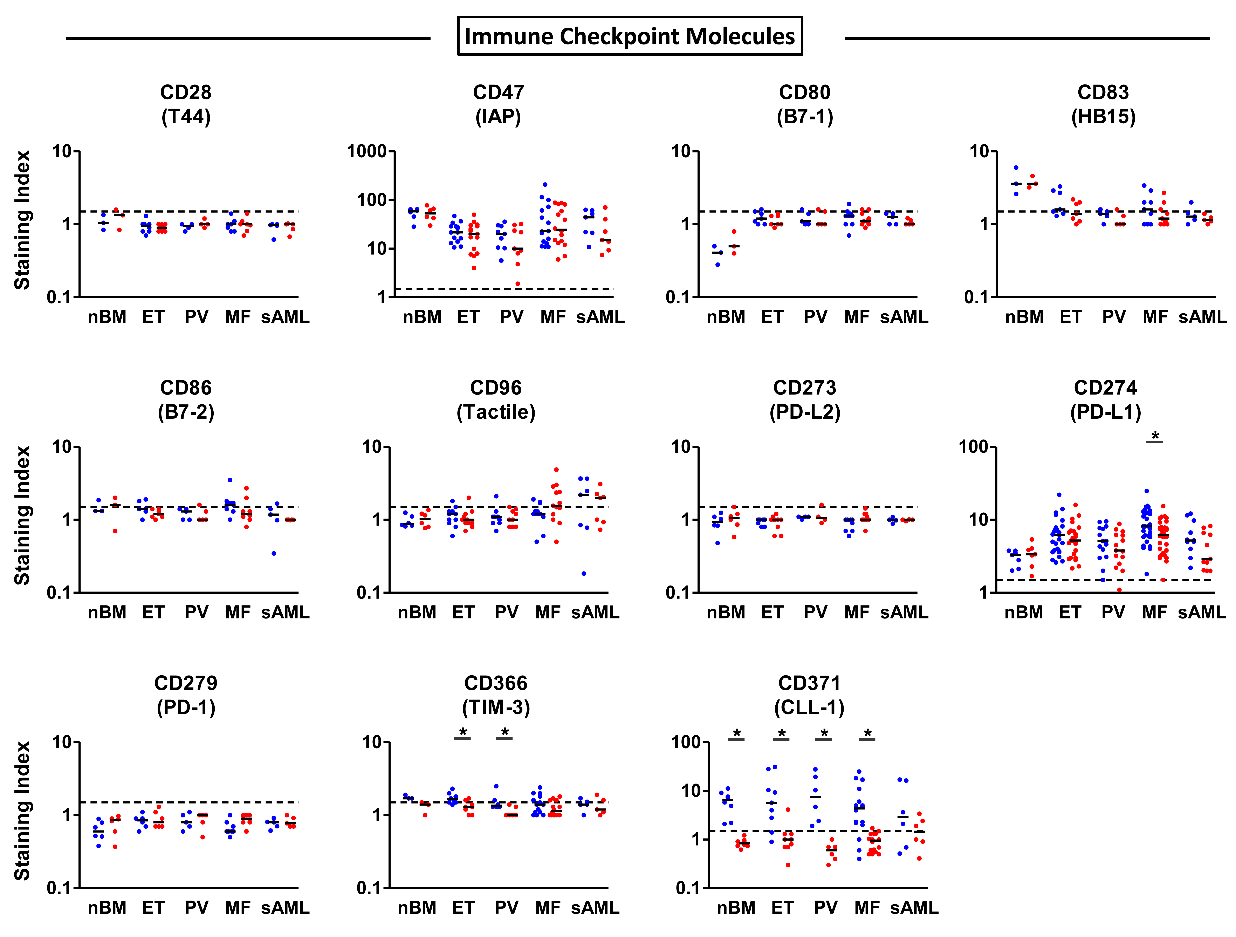
Figure S7 continued**

**Evaluation of surface marker- and target expression profiles in MPN stem- and progenitor cells**

Expression of cell surface antigens on CD34^+^/CD38^+^ progenitor cells (blue dots) and CD34^+^/CD38^−^ stem cells (red dots) obtained from patients with ET, PV, MF and sAML was determined by multicolor flow cytometry (Figure S1). CD34^+^/CD38^+^ cells and CD34^+^/CD38^−^ cells obtained from healthy donors (nBM) served as controls. Results are expressed as staining index (median fluorescence intensity of the indicated marker divided by the median fluorescence intensity of the isotype control). Each symbol represents a single donor. Horizontal lines show the median expression level in each cohort. Dotted horizontal lines represent the cut-off values for negativity (staining index <1.5). Significance levels of differences in expression of markers and targets between stem and progenitor cells were analyzed by Mann-Whitney U-test (*, p<0.05). Abbreviations: C1qR1, complement C1q receptor; CD, cluster of differentiation; CLL-1, C-type lectin-like molecule-1; CSF2RB, colony-stimulating factor 2 receptor-beta; CXCR4, chemokine C-X-C motif receptor 4; DPPIV, dipeptidyl peptidase IV; EPOR, erythropoietin receptor; ET, essential thrombocythemia; FLT3, FMS-like tyrosine kinase 3; G-CSFR, granulocyte colony-stimulating factor receptor; IAP, integrin associated protein; IGF-1R, insulin-like growth factor 1 receptor; IL-1RAP, interleukin-1 receptor accessory protein; IL-2RA, interleukin-2 receptor alpha chain; IL-3RA, interleukin-3 receptor alpha chain; M-CSFR, macrophage colony-stimulating factor receptor; MDR-1, multidrug resistance protein 1; MF, myelofibrosis; MPN, myeloproliferative neoplasm; MRC1, mannose receptor C-type 1; nBM, normal bone marrow; NGF-R, nerve growth factor receptor; OSMRb, oncostatin M receptor beta; PD-1, programmed death protein 1; PD-L1, programmed death ligand 1; PD-L2, programmed death ligand 2; PV, polycythemia vera; sAML, secondary acute myeloid leukemia following MPN; TIM-3, T cell immunoglobulin and mucin domain-containing protein 3; TPOR, thrombopoietin receptor; VEGFR-2, vascular endothelial growth factor receptor 2.

**Figure S8**


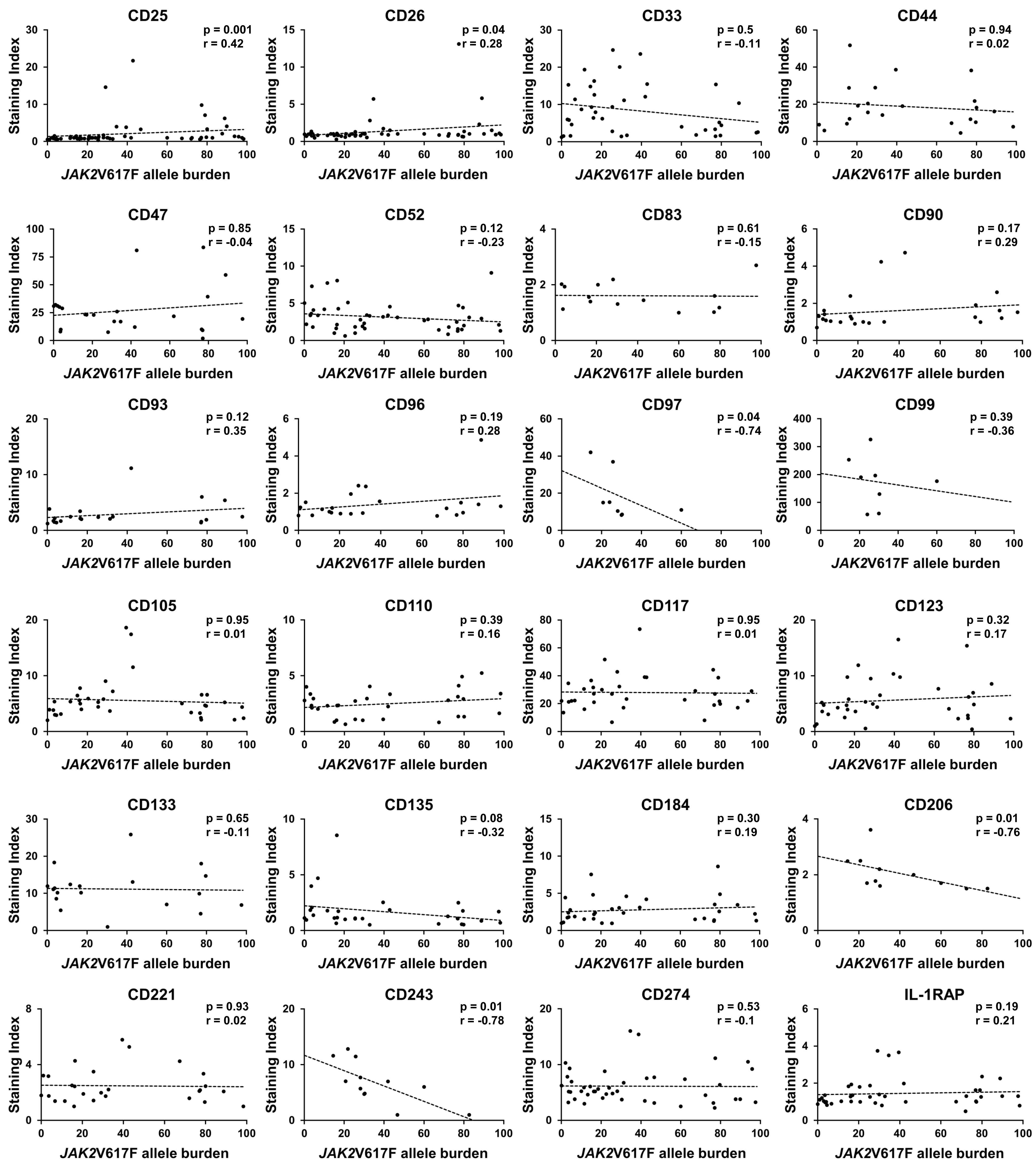


**Correlation between expression of cell surface markers on NSC and *JAK2*V617F allele burden in patients with MPN**

Samples from patients with MPN were stained with fluorochrome-labeled monoclonal antibodies and stem cells were identified as CD34^+^/CD38^−^ cells (Figure S1). Expression of markers and targets on MPN NSC was analyzed by multicolor flow cytometry. Results are expressed as staining index (median fluorescence intensity of the indicated marker divided by the median fluorescence intensity of the isotype control). Each symbol represents a single donor. *JAK2*V617F allele burden was measured by real-time PCR using genomic DNA as part of the routine diagnostic workup for patients with MPN. Dotted lines represent the calculated linear regression curves. Abbreviations: IL-1RAP, interleukin-1 receptor accessory protein; MPN, myeloproliferative neoplasm; NSC, neoplastic stem cells; p, p-value; r, Spearman's rank correlation coefficient.

**Figure S9**


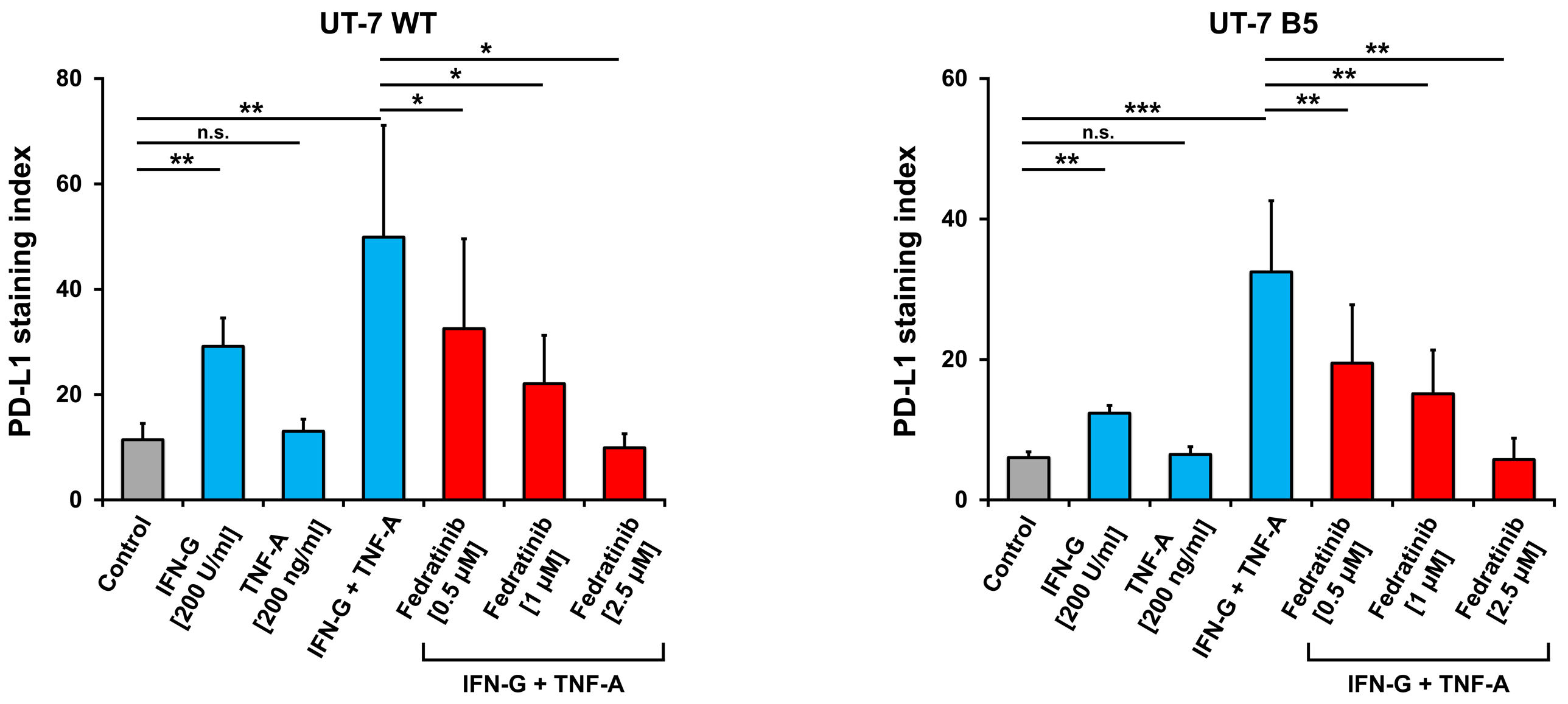


**Fedratinib downregulates cytokine-induced PD-L1 expression on MPN cells**

UT-7 cells engineered to display wild type *CALR* (UT-7 WT) or the *CALR* del61/del25 mutant (UT-7 B5) were incubated in control medium or medium containing either IFN-G (200 U/ml), TNF-A (200 ng/ml) or combination of both cytokines with or without fedratinib (0.5–2.5 µM) at 37°C for 24 hours. Expression of PD-L1 was determined by multicolor flow cytometry and presented as staining index (median fluorescence intensity of PD-L1 divided by the median fluorescence intensity of the isotype control). Results represent the mean±SD from at least three independent experiments. Significance levels of differences in expression of PD-L1 between the different conditions were analyzed by Student's t-test (*, p<0.05; **, p<0.01; ***, p<0.001). Abbreviations: IFN-G, interferon-gamma; MPN, myeloproliferative neoplasm; n.s., not significant; PD-L1, programmed death ligand 1; SD, standard deviation; TNF-A, tumor necrosis factor-alpha.

**Figure S10**





**Ruxolitinib downregulates cytokine-induced PD-L1 expression on MPN cells**

(A) *JAK2*V617F+ cell lines HEL and SET-2 or (B) UT-7 cells engineered to display wild type *CALR* (UT-7 WT) or the *CALR* del61/del25 mutant (UT-7 B5) were incubated in control medium or medium containing either IFN-G (200 U/ml), TNF-A (200 ng/ml) or combination of both cytokines with or without ruxolitinib (0.5–2.5 µM) at 37°C for 24 hours. Expression of PD-L1 was determined by multicolor flow cytometry and presented as staining index (median fluorescence intensity of PD-L1 divided by the median fluorescence intensity of the isotype control). Results represent the mean±SD from at least three independent experiments. Significance levels of differences in expression of PD-L1 between the different conditions were analyzed by Student's t-test (*, p<0.05; **, p<0.01; ***, p<0.001). Abbreviations: IFN-G, interferon-gamma; MPN, myeloproliferative neoplasm; n.s., not significant; PD-L1, programmed death ligand 1; SD, standard deviation; TNF-A, tumor necrosis factor-alpha.

**Figure S11**





**JQ1 downregulates cytokine-induced PD-L1 expression on MPN cells**

(A) *JAK2*V617F+ cell lines HEL and SET-2, (B) UT-7 cells engineered to display wild type *CALR* (UT-7 WT) or the *CALR* del61/del25 mutant (UT-7 B5) or (C) primary MNC from *JAK2*V617F+ MPN patients, were incubated in control medium or medium containing either IFN-G (200 U/ml), TNF-A (200 ng/ml) or combination of both cytokines with or without JQ1 (0.25–2.5 µM) at 37°C for 24 hours. Expression of PD-L1 was determined by multicolor flow cytometry and presented as staining index (median fluorescence intensity of PD-L1 divided by the median fluorescence intensity of the isotype control). Results represent the mean±SD from at least three independent experiments. Significance levels of differences in expression of PD-L1 between the different conditions were analyzed by Student's t-test (*, p<0.05; **, p<0.01; ***, p<0.001). Abbreviations: IFN-G, interferon-gamma; MNC, mononuclear cells; MPN, myeloproliferative neoplasm; n.s., not significant; PD-L1, programmed death ligand 1; SD, standard deviation; TNF-A, tumor necrosis factor-alpha.

**Figure S12**





**dBET6 downregulates cytokine-induced PD-L1 expression on MPN cells**

(A) *JAK2*V617F+ cell lines HEL and SET-2, (B) UT-7 cells engineered to display wild type *CALR* (UT-7 WT) or the *CALR* del61/del25 mutant (UT-7 B5) or (C) primary MNC from *JAK2*V617F+ MPN patients, were incubated in control medium or medium containing either IFN-G (200 U/ml), TNF-A (200 ng/ml) or combination of both cytokines with or without dBET6 (0.01-1 µM) at 37°C for 24 hours. Expression of PD-L1 was determined by multicolor flow cytometry and presented as staining index (median fluorescence intensity of PD-L1 divided by the median fluorescence intensity of the isotype control). Results represent the mean±SD from at least three independent experiments. Significance levels of differences in expression of PD-L1 between the different conditions were analyzed by Student's t-test (*, p<0.05; **, p<0.01; ***, p<0.001). Abbreviations: IFN-G, interferon-gamma; MNC, mononuclear cells; MPN, myeloproliferative neoplasm; n.s., not significant; PD-L1, programmed death ligand 1; SD, standard deviation; TNF-A, tumor necrosis factor-alpha.

**Figure S13**


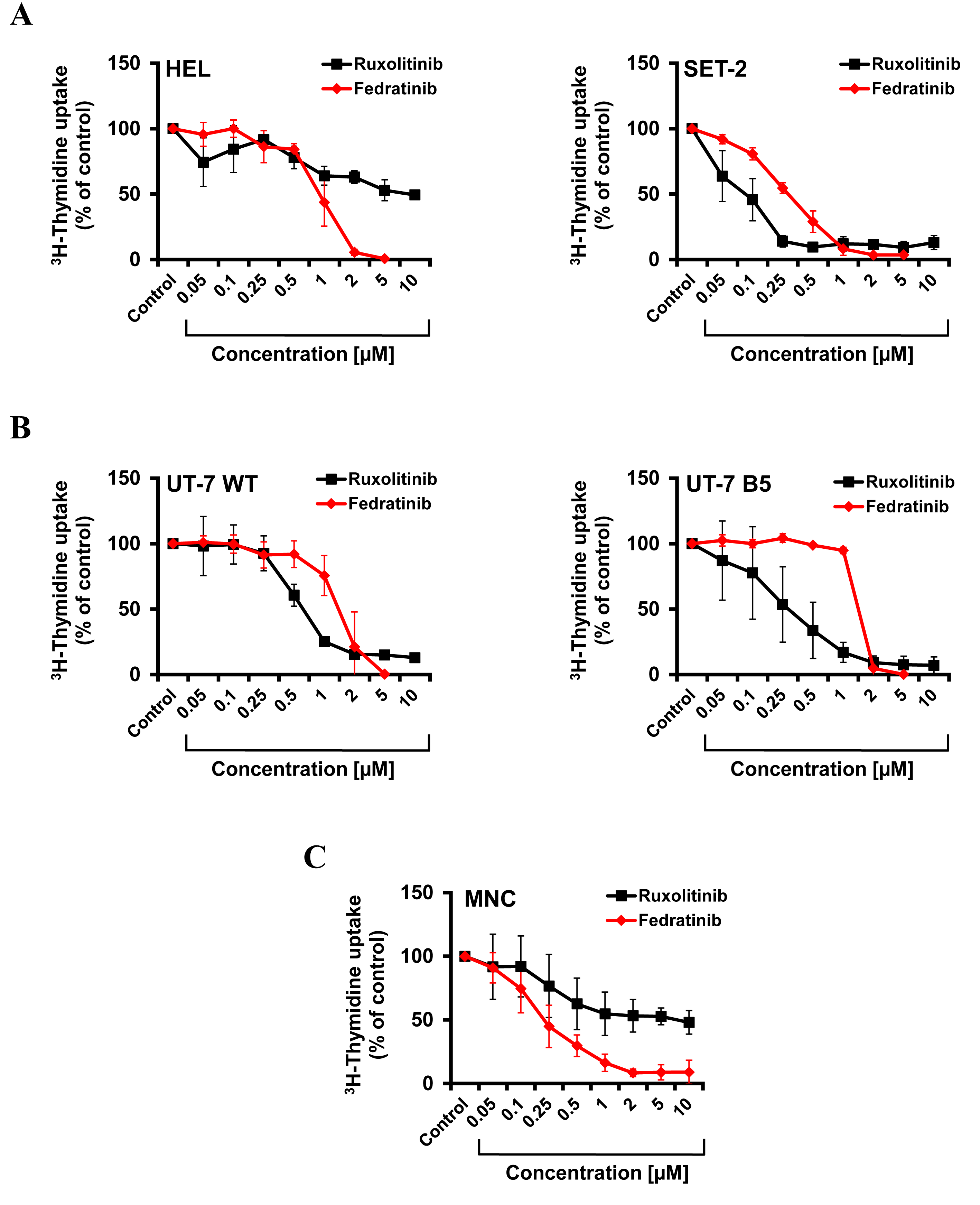


**Effects of JAK2 inhibitors on proliferation of MPN cells**

(A) The *JAK2*V617F+ MPN cell lines HEL and SET-2, (B) UT-7 cells engineered to display wild type *CALR* (UT-7 WT) or the *CALR* del61/del25 mutant (UT-7 B5), and (C) primary MPN MNC obtained from patients with chronic phase MPN were incubated in control medium containing DMSO or various concentrations of ruxolitinib (0.05-10 µM) or fedratinib (0.05-10 µM) at 37ºC for 48 hours. Then, ^3^H-thymidine was added and 16 hours later, bound radioactivity was measured in a β-counter. Results are expressed as percent of control and represent the mean±SD from at least three independent experiments. Abbreviations: DMSO, dimethyl sulfoxide; MNC, mononuclear cells; MPN, myeloproliferative neoplasm.

**Figure S14**


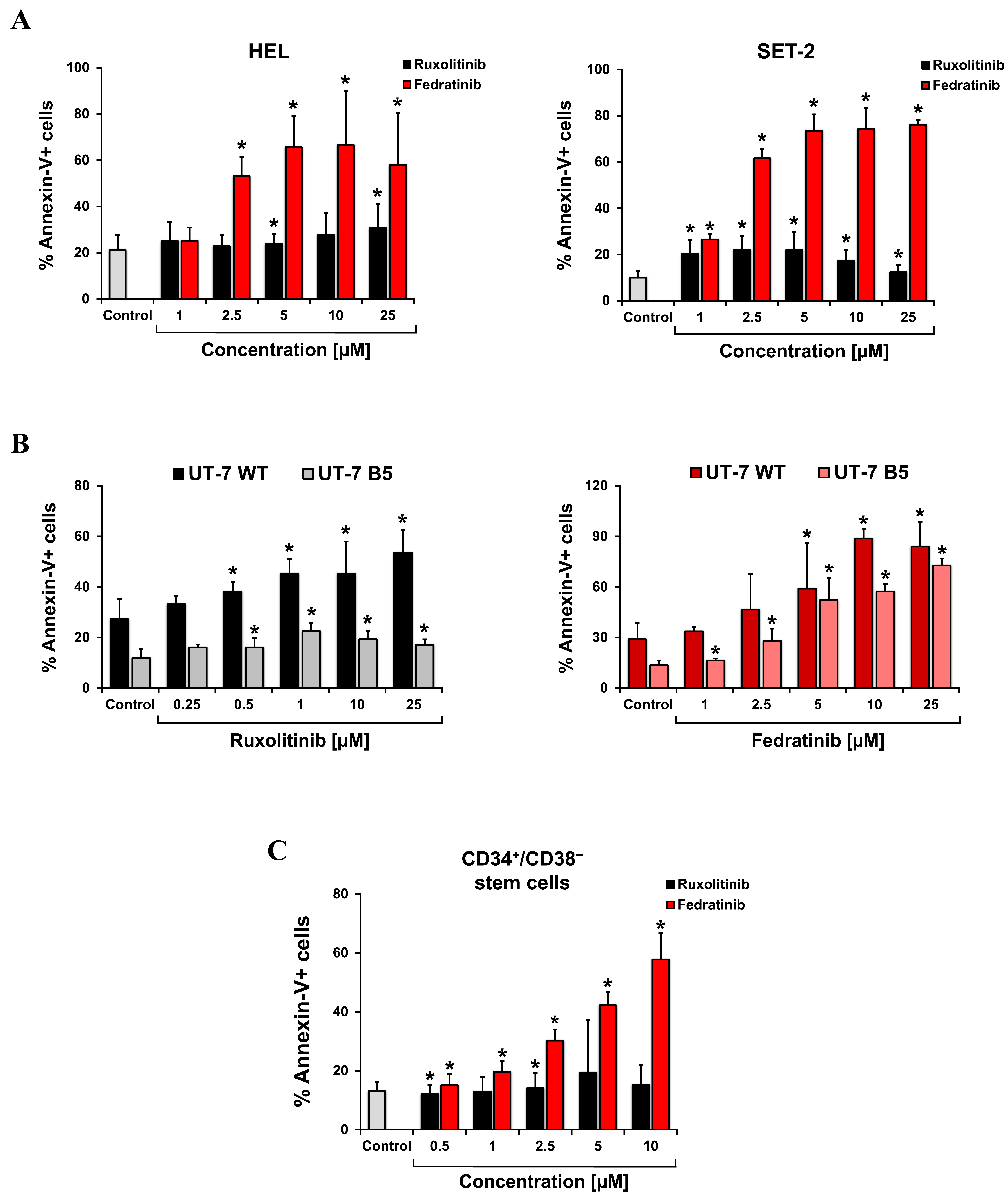


**Effects of JAK2 inhibitors on survival of MPN-related cell lines and MPN NSC**

(A) The *JAK2*V617F+ MPN cell lines HEL and SET-2, (B) UT-7 cells engineered to express wild type *CALR* (UT-7 WT) or the *CALR* del61/del25 mutant (UT-7 B5), and (C) primary MPN MNC obtained from patients with chronic phase MPN were incubated in control medium containing DMSO or various concentrations of ruxolitinib (0.25-25 µM) or fedratinib (0.5-25 µM) at 37ºC for 24 hours. Then, the percentage of apoptotic cells was measured by multicolor flow cytometry staining for Annexin V and DAPI. Bars show the mean±SD of Annexin-V+ cells from at least three independent experiments. Significance levels of differences in percent of apoptotic cells between the various conditions were analyzed by Student’s t test (*, p<0.05 compared to control). Abbreviations: DAPI, 4′,6-diamidino-2-phenylindole; DMSO, dimethyl sulfoxide; MNC, mononuclear cells; MPN, myeloproliferative neoplasm; SD, standard deviation.

**Figure S15**


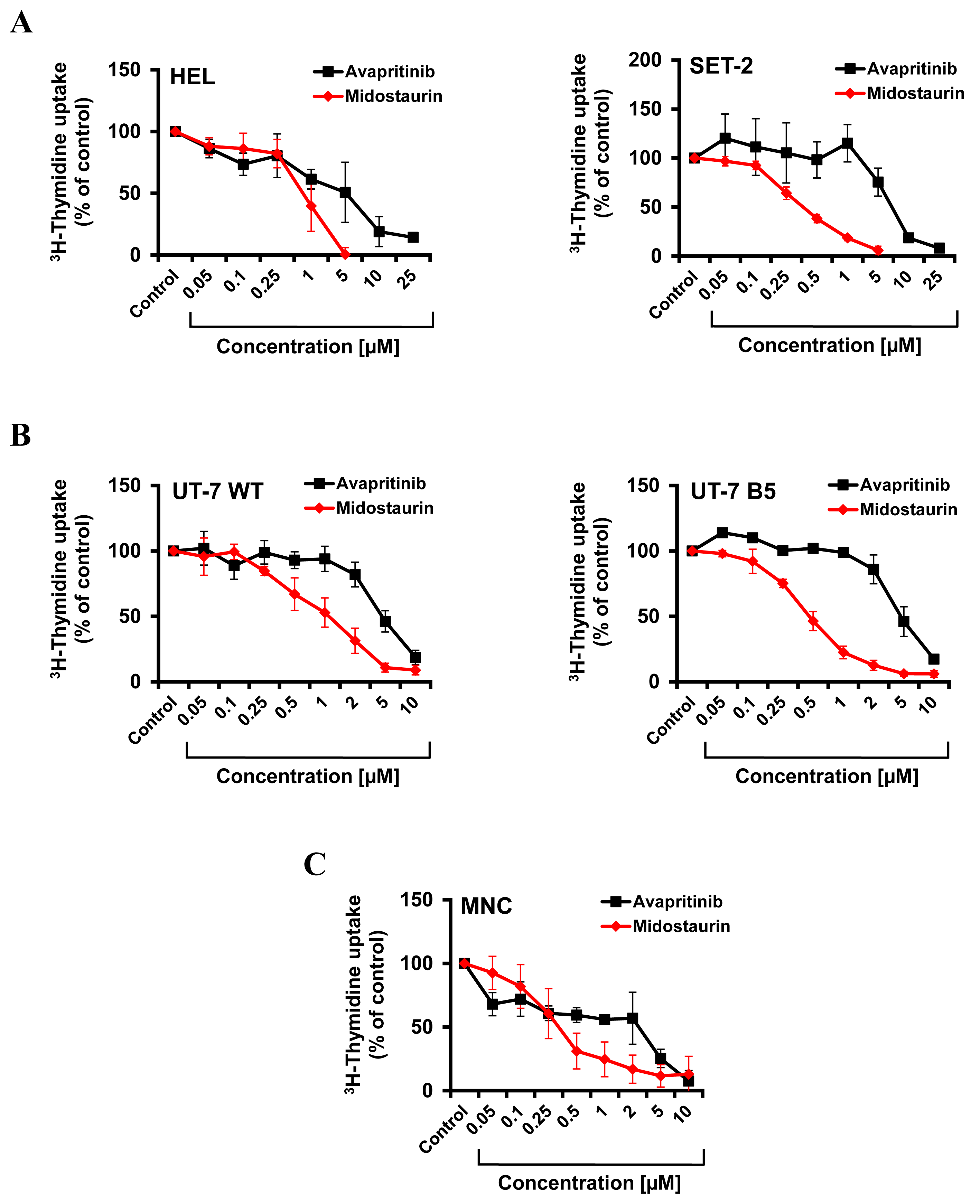


**Effects of KIT inhibitors on proliferation of MPN cells**

(A) The *JAK2*V617F+ MPN cell lines HEL and SET-2, (B) UT-7 cells engineered to display wild type *CALR* (UT-7 WT) or the *CALR* del61/del25 mutant (UT-7 B5), and (C) primary MPN MNC obtained from patients with chronic phase MPN were incubated in control medium containing DMSO or various concentrations of avapritinib (0.05-25 µM) or midostaurin (0.05-10 µM) at 37ºC for 48 hours. Then, ^3^H-thymidine was added, and 16 hours later, bound radioactivity was measured in a β-counter. Results are expressed as percent of control and represent the mean±SD from at least three independent experiments. Abbreviations: DMSO, dimethyl sulfoxide; MNC, mononuclear cells; MPN, myeloproliferative neoplasm; SD, standard deviation.

**Figure S16**





**Effects of KIT inhibitors on survival of MPN cells**

(A) The *JAK2*V617F+ MPN cell lines HEL and SET-2, (B) UT-7 cells engineered to express wild type *CALR* (UT-7 WT) or the *CALR* del61/del25 mutant (UT-7 B5), and (C) primary MPN MNC obtained from patients with chronic phase MPN were incubated in control medium containing DMSO or various concentrations of avapritinib (0.5-25 µM) or midostaurin (0.5-25µM) at 37ºC for 24 hours. Thereafter, the percentage of apoptotic cells was analyzed by multicolor flow cytometry staining for Annexin V and DAPI. Bars show the mean±SD of Annexin-V+ cells from at least three independent experiments. Significance levels of differences in percent of apoptotic cells between the various conditions were analyzed by Student’s t test (*, p<0.05 compared to control). Abbreviations: DMSO, dimethyl sulfoxide; MNC, mononuclear cells; MPN, myeloproliferative neoplasm; SD, standard deviation.

**Figure S17**


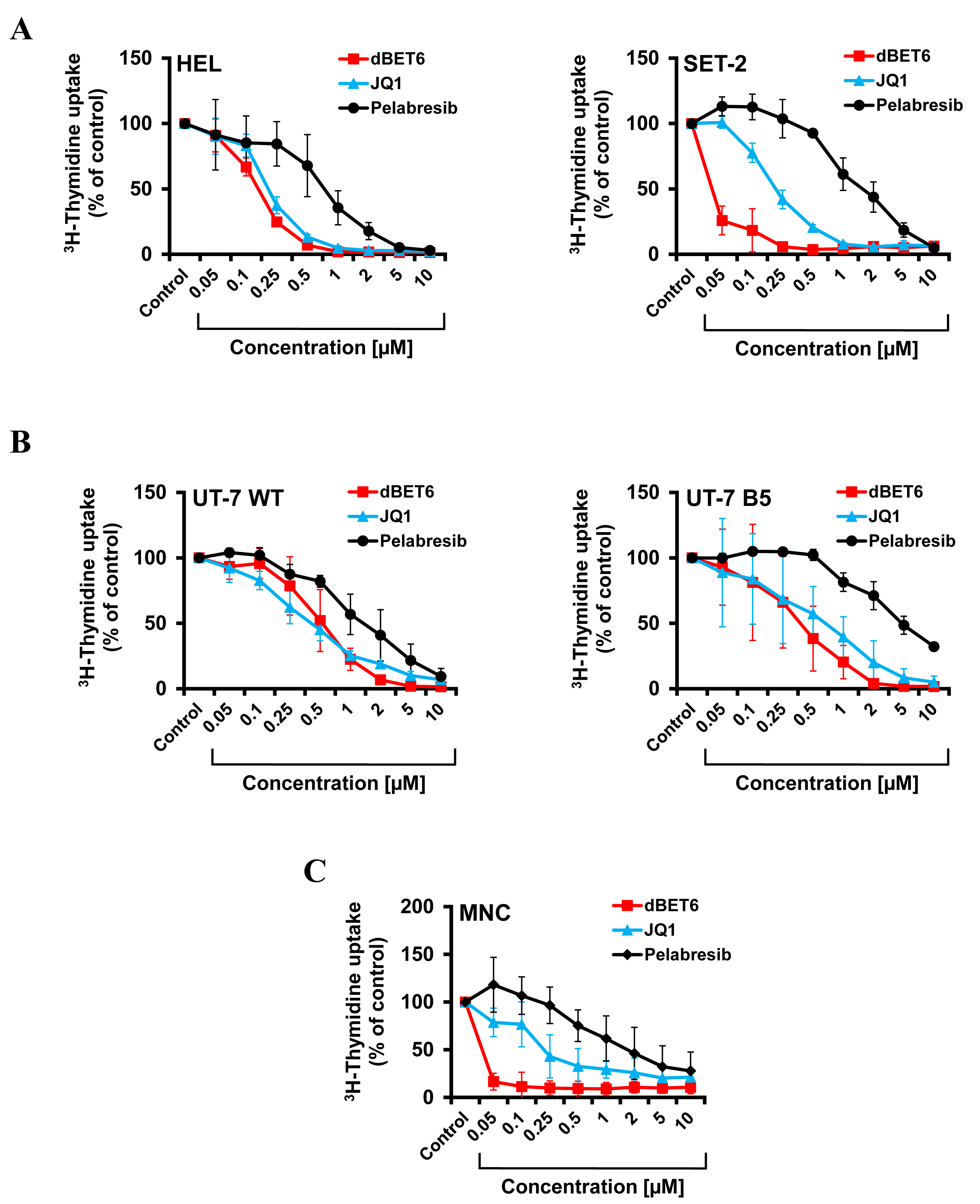


**Effects of BRD4-targeting drugs on proliferation of MPN cells**

(A) The *JAK2*V617F+ MPN cell lines HEL and SET-2, (B) UT-7 cells engineered to display wild type *CALR* (UT-7 WT) or the *CALR* del61/del25 mutant (UT-7 B5), and (C) primary MPN MNC obtained from patients with chronic phase MPN were incubated in control medium containing DMSO or various concentrations of pelabresib (0.05-10 µM), JQ1 (0.05-10 µM) or dBET6 (0.05-10 µM) at 37ºC for 48 hours. ^3^H-thymidine was added and 16 hours later, bound radioactivity was measured in a β-counter. Results are expressed as percent of control and represent the mean±SD from at least three independent experiments. Abbreviations: DMSO, dimethyl sulfoxide; MNC, mononuclear cells; MPN, myeloproliferative neoplasm; SD, standard deviation.

**Figure S18**





**Effects of BRD4-targeting drugs on survival of MPN cells**

(A) The *JAK2*V617F+ MPN cell lines HEL and SET-2, (B) UT-7 cells engineered to express wild type *CALR* (UT-7 WT) or the *CALR* del61/del25 mutant (UT-7 B5), and (C) primary MPN MNC obtained from patients with chronic phase MPN were incubated in control medium containing DMSO or various concentrations of pelabresib (0.5-10 µM), JQ1 (0.5-10 µM) and dBET6 (0.5-10 µM) at 37ºC for 24 hours. Thereafter, apoptosis induction was measured by multicolor flow cytometry staining for Annexin V and DAPI. Bars show the mean±SD of Annexin-V+ cells from at least three independent experiments. Significance levels of differences in percent of apoptotic cells between the various conditions were analyzed by Student’s t test (*, p<0.05 compared to control). Abbreviations: DMSO, dimethyl sulfoxide; MNC, mononuclear cells; MPN, myeloproliferative neoplasm; SD, standard deviation.

**Figure S19**


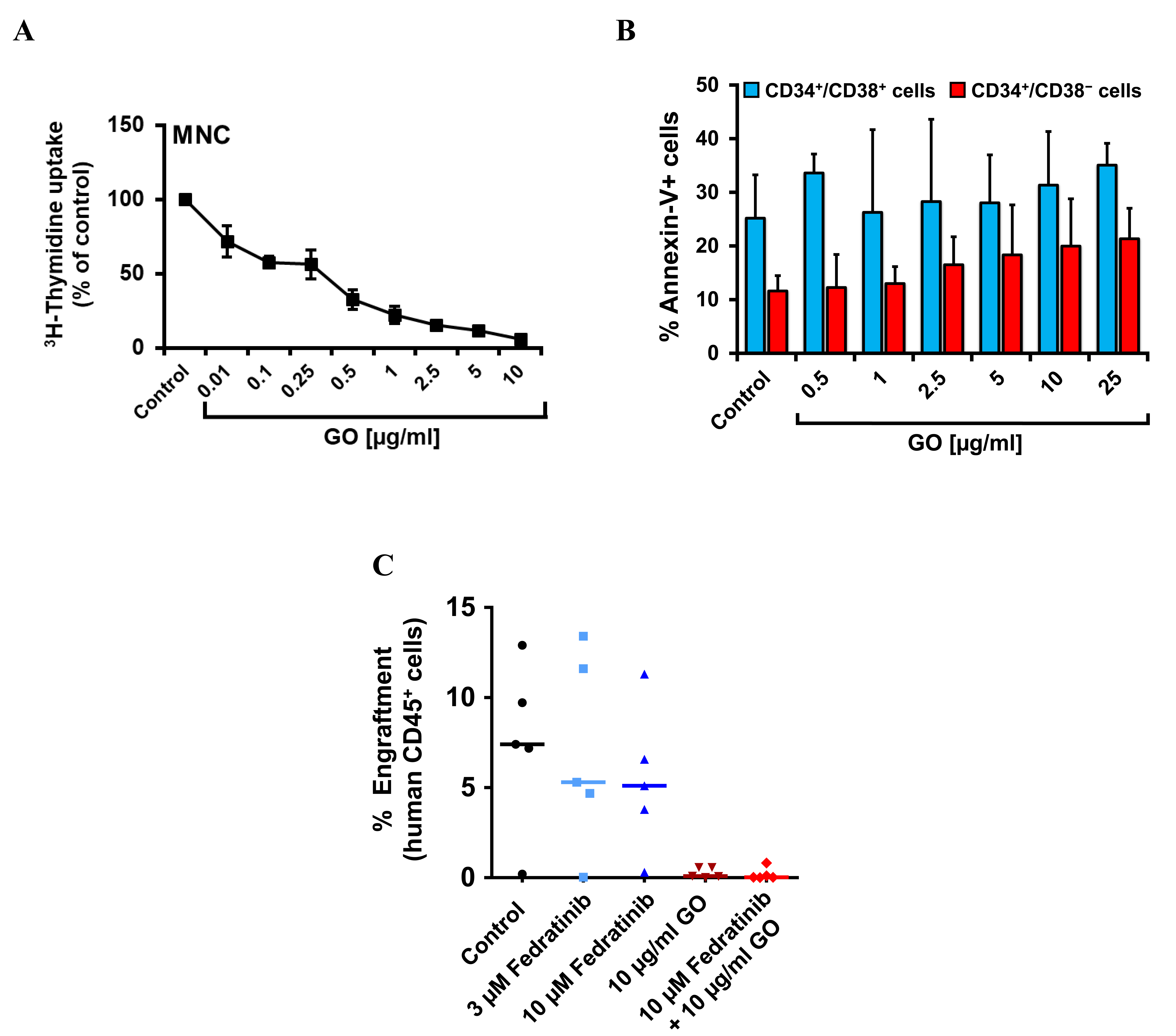


**Effects of the CD33-targeted drug gemtuzumab ozogamicin (GO) on growth, survival, and engraftment of human MPN cells**

(A) Primary MPN MNC obtained from patients with chronic phase MPN were incubated in control medium containing DMSO or various concentrations of GO at 37ºC for 48 hours. ^3^H-thymidine was added and 16 hours later, bound radioactivity was measured in a β-counter. Results are expressed as percent of control and represent the mean±SD from at least three independent experiments. (B) Primary MPN MNC obtained from patients with chronic phase MPN were incubated in control medium containing DMSO or various concentrations of GO at 37ºC for 24 hours. Thereafter, apoptosis induction was measured by multicolor flow cytometry staining for Annexin V and DAPI. Bars show the mean±SD of Annexin-V+ cells from at least three independent experiments. (C) MNC were obtained from the peripheral blood of a patient with primary myelofibrosis (#104). After T cell depletion, the cells we incubated with either fedratinib (3 µM and 10 µM), GO (10 µg/ml) or combination of both (10 µM fedratinib and 10 µg/ml GO) for 1 hour at 37˚C. Afterwards, the cells were washed and injected intravenously into sublethally irradiated NSGS mice. After 30 weeks the mice were sacrificed and the engraftment of human CD45^+^ cells (expressed as percentage of all flushed bone marrow cells) was analyzed by multicolor flow cytometry (each symbol represents engraftment in an individual mouse). The horizontal lines show the median percentage levels of engrafted human CD45^+^ cells. Abbreviations: DMSO, dimethyl sulfoxide; GO, gemtuzumab-ozogamicin; MNC, mononuclear cells; MPN, myeloproliferative neoplasm; SD, standard deviation.

III**. Supplementary Tables**

## Table S1

## Specification of monoclonal antibodies used in multicolor flow cytometry experiments

| Antigen | CD | Clone | Conjugate | Species, Isotype | Manufacturer |
| --- | --- | --- | --- | --- | --- |
|  |  |  |  |  |  |
| Isotype control | n.c. | MOPC-21 | PE | Mouse, IgG1 | BD Biosciences |
| Isotype control | n.c. | MOPC-21 | APC | Mouse, IgG1 | BD Biosciences |
| Isotype control | n.c. | 20102 | PE | Mouse, IgG2A | R&D Systems |
| Isotype control | n.c. | 133303 | PE | Mouse, IgG2B | R&D Systems |
| B4 | CD19 | SJ25C1 | APC | Mouse, IgG1 | BD Biosciences |
| IL-2RA | CD25 | 2A3 | PE | Mouse, IgG1 | BD Biosciences |
| DPPIV | CD26 | MA261 | PE | Mouse, IgG1 | BD Biosciences |
| T44 | CD28 | cd28.2 | PE | Mouse, IgG1 | BioLegend |
| Siglec-3 | CD33 | WM53 | PE | Mouse, IgG1 | BD Biosciences |
| HPCA-1 | CD34 | 581 | FITC | Mouse, IgG1 | BioLegend |
| T10 | CD38 | HIT2 | APC | Mouse, IgG1 | BD Biosciences |
| Hermes | CD44 | 515 | PE | Mouse, IgG1 | BD Biosciences |
| LCA | CD45 | HI30 | V500 | Mouse, IgG1 | BD Biosciences |
| LCA | CD45 | 2D1 | APC-H7 | Mouse, IgG1 | BD Biosciences |
| IAP | CD47 | B6H12 | PE | Mouse, IgG1 | BD Biosciences |
| Campath-1 | CD52 | HI186 | PE | Mouse, IgG2b | BioLegend |
| B7-1 | CD80 | 2D10 | PE | Mouse, IgG1 | BioLegend |
| HB15 | CD83 | HB15E | PE | Mouse, IgG1 | BioLegend |
| B7-2 | CD86 | IT2.2 | PE | Mouse, IgG2b | BioLegend |
| Thy-1 | CD90 | 5E10 | PE | Mouse, IgG1 | BD Biosciences |
| C1qR1 | CD93 | VIMD2 | PE | Mouse, IgG1 | BioLegend |
| Tactile | CD96 | NK92.39 | PE | Mouse, IgG1 | Affymetrix |
| ADGRE5 | CD97 | VIM3b | PE | Mouse, IgG1 | BioLegend |
| MIC2 | CD99 | 3B2/TA8 | PE | Mouse, IgG2a | BioLegend |
| Endoglin | CD105 | 166707 | PE | Mouse, IgG1 | R&D Systems |
| TPOR | CD110 | 167639 | PE | Mouse, IgG2a | R&D Systems |
| G-CSFR | CD114 | LMM741 | PE | Mouse, IgG1 | BD Biosciences |
| M-CSFR | CD115 | 61708 | PE | Mouse, IgG1 | R&D Systems |
| KIT | CD117 | 104D2 | PE | Mouse, IgG1 | BD Biosciences |
| IL-3RA | CD123 | 7G3 | PE | Mouse, IgG2a | BD Biosciences |
| CSF2RB | CD131 | 1C1 | PE | Mouse, IgG1 | Abcam |
| Prominin-1 | CD133 | AC133 | PE | Mouse, IgG1 | Miltenyi Biotec |
| FLT3 | CD135 | BV10A4H2 | PE | Mouse, IgG1 | BioLegend |
| CXCR4 | CD184 | 12G5 | PE | Mouse, IgG2a | BioLegend |
| MRC1 | CD206 | 15-2 | PE | Mouse, IgG1 | BioLegend |
| IGF-1R | CD221 | 33255 | PE | Mouse, IgG1 | R&D Systems |
| MDR-1 | CD243 | UIC2 | PE | Mouse, IgG2a | BioLegend |
| NGF-R | CD271 | C40-1457 | PE | Mouse, IgG1 | BD Biosciences |
| PD-L2 | CD273 | MIH18 | PE | Mouse, IgG1 | BioLegend |
| PD-L1 | CD274 | 29E.2A3 | PE | Mouse, IgG2b | BioLegend |
| PD-1 | CD279 | eh12.2h7 | PE | Mouse, IgG1 | BioLegend |
| VEGFR-2 | CD309 | 89106 | PE | Mouse, IgG1 | R&D Systems |
| TIM-3 | CD366 | F38-2E2 | PE | Mouse, IgG1 | BioLegend |
| CLL-1 | CD371 | 50C1 | PE | Mouse, IgG2a | BD Biosciences |
| IL-1RAP | n.c. | 89412 | PE | Mouse, IgG1 | R&D Systems |
| EPOR | n.c. | 38409 | PE | Mouse, IgG2b | R&D Systems |
| MET | n.c | 95106 | PE | Mouse, IgG1 | R&D Systems |
| OSMRb | n.c. | AN-U2 | PE | Mouse, IgG1 | Santa Cruz |

Abbreviations: APC, allophycocyanin; C1qR1, complement C1q receptor; CD, cluster of differentiation; CSF2RB, colony-stimulating factor 2 receptor-beta; CLL-1, C-type lectin-like molecule-1; CXCR4, chemokine C-X-C motif receptor 4; DPPIV, dipeptidyl peptidase IV; EPOR, erythropoietin receptor; FITC, fluorescein isothiocyanate; FLT3, FMS-like tyrosine kinase 3; G-CSFR, granulocyte colony-stimulating factor receptor; IAP, integrin associated protein; Ig, immunoglobulin; IGF-1R, insulin-like growth factor 1 receptor; IL-1RAP, interleukin-1 receptor accessory protein; IL-2RA, interleukin-2 receptor alpha chain; IL-3RA, interleukin-3 receptor alpha chain; LCA, leukocyte common antigen; M-CSFR, macrophage colony-stimulating factor receptor; MDR-1, multidrug resistance protein 1; MRC1, mannose receptor C-type 1; n.c., not (yet) clustered; NGF-R, nerve growth factor receptor; OSMRb, oncostatin M receptor beta; PE, phycoerythrin; PD-1, programmed death protein 1; PD-L1, programmed death ligand 1; PD-L2, programmed death ligand 2; TIM-3, T cell immunoglobulin and mucin domain-containing protein 3; TPOR, thrombopoietin receptor; VEGFR-2, vascular endothelial growth factor receptor 2.

Company Locations: BD Bioscience, San José, CA, USA; R&D Systems, Minneapolis, MN, USA; BioLegend, San Diego, CA, USA; Affymetrix, Santa Clara, California, USA; Abcam, Cambridge, UK; Miltenyi Biotec, Bergisch Gladbach, Germany; Santa Cruz Biotechnology, Dallas, TX, USA.

## Table S2

## Patients’ characteristics

| **#** | **Diagnosis** | **Age** | **Gender** | **Driver mutation**  **Mutated Gene** | **WBC (G/L)** | **Hb (g/dL)** | **PLT (G/L)** | **LDH (U/L)** | **PB blasts (%)** | **BM blasts (%)** | **Grade of fibrosis** | **Splenomegaly** |
| --- | --- | --- | --- | --- | --- | --- | --- | --- | --- | --- | --- | --- |
| 1 | ET | 31 | f | *JAK2*V617F | 8 | 14 | 542 | 172 | 0 | 2 | 0 | n.a. |
| 2 | ET | 38 | f | *JAK2*V617F | 8.79 | 14.7 | 545 | 185 | 0 | 1 | 0 | no |
| 3 | ET | 72 | f | *JAK2*V617F | 6.93 | 10.8 | 547 | 175 | 0 | 1 | 0 | n.a. |
| 4 | ET | 48 | m | *JAK2*V617F | 6.59 | 16.6 | 818 | 224 | 0 | 1 | 0 | no |
| 5 | ET | 49 | m | *JAK2*V617F | 6.84 | 13.2 | 545 | 172 | 0 | 2 | 0 | yes |
| 6 | ET | 68 | m | *JAK2*V617F | 5.51 | 14.8 | 358 | 193 | 0 | 1 | 0 | no |
| 7 | ET | 67 | f | *JAK2*V617F | 7.76 | 13.3 | 1016 | 153 | 0 | 3 | 0 | no |
| 8 | ET | 68 | m | *MPL* | 7.17 | 14.6 | 565 | 228 | 0 | 5 | 0 | no |
| 9 | ET | 32 | f | n.a. | 11.76 | 10.4 | 890 | 195 | 0 | 1 | 0 | no |
| 10 | ET | 61 | m | *JAK2*V617F | 7.8 | 14.9 | 930 | 160 | 0 | 1 | 0 | no |
| 11 | ET | 58 | f | *JAK2*V617F | 5.09 | 14.6 | 405 | 217 | 0 | 1 | 0 | no |
| 12 | ET | 33 | m | *CALR* (Type 1) | 10.4 | 13 | 377 | 210 | 2 | 2.5 | 1 | no |
| 13 | ET | 30 | m | *CALR* (Type 1) | 6.84 | 15.9 | 734 | 187 | 0 | 1 | 0 | no |
| 14 | ET | 32 | m | *JAK2*V617F | 9.2 | 16.9 | 769 | 263 | 0 | 0 | 1 | no |
| 15 | ET | 64 | f | *JAK2*V617F and *CALR* | 8.1 | 12.5 | 1817 | 185 | 0 | 1 | 0 | no |
| 16 | ET | 82 | m | *CALR* (Type 1) | 11.4 | 13.3 | 726 | 207 | 0 | 0 | 0 | no |
| 17 | ET | 52 | m | *JAK2*V617F | 7.31 | 13.5 | 456 | 140 | 0 | 1 | 0 | no |
| 18 | ET | 76 | m | n.a. | 9.5 | 14 | 1211 | 284 | 0 | 0 | 0 | no |
| 19 | ET | 36 | m | *JAK2*V617F | 15.05 | 15.4 | 691 | 208 | 0 | 1 | 0 | no |
| 20 | ET | 55 | f | *CALR* (Type 1) | 7.97 | 13 | 674 | 278 | 0 | 1 | 0 | no |
| 21 | ET | 36 | m | *JAK2*V617F | 18.35 | 17 | 1355 | 187 | 0 | 1 | 0 | no |
| 22 | ET | 52 | m | *JAK2*V617F | 10.63 | 17.4 | 591 | 183 | 0 | 5 | 0 | no |
|  |  |  |  |  |  |  |  |  |  |  |  |  |
|  |  |  |  |  |  |  |  |  |  |  |  |  |
|  |  |  |  |  |  |  |  |  |  |  |  |  |
| **#** | **Diagnosis** | **Age** | **Gender** | **Driver mutation**  **Mutated Gene** | **WBC (G/L)** | **Hb (g/dL)** | **PLT (G/L)** | **LDH (U/L)** | **PB blasts (%)** | **BM blasts (%)** | **Grade of fibrosis** | **Splenomegaly** |
| 23 | ET | 68 | m | *JAK2*V617F | 12.69 | 10.9 | 1407 | 241 | 0 | 3 | 0 | no |
| 24 | ET | 65 | f | *JAK2*V617F | 6.9 | 12.6 | 556 | 167 | 0 | 2 | 0 | no |
| 25 | ET | 59 | m | *CALR* (Type 1) | 12.82 | 14.9 | 1534 | 274 | 0 | 1 | 0 | yes |
| 26 | ET | 45 | m | *JAK2*V617F | 9.6 | 13.6 | 682 | 196 | 0 | 4.7 | 0 | no |
| 27 | ET | 42 | f | *JAK2*V617F | 12.2 | 13.4 | 1153 | 205 | 0 | n.a. | 1 | no |
| 28 | ET | 75 | f | *JAK2*V617F | 15 | 8.8 | 1243 | 295 | 0 | 0.5 | 0 | no |
| 29 | ET | 83 | m | *JAK2*V617F | 16.1 | 13.7 | 482 | n.a. | 0 | 1.5 | 0 | no |
| 30 | ET | 78 | m | *CALR* (Type 2) | 5.3 | 14 | 922 | 170 | 0 | n.a. | 1 | no |
| 31 | ET | 48 | m | *JAK2*V617F | 7.1 | 16.7 | 749 | 203 | 0 | n.a. | n.a. | no |
| 32 | ET | 45 | f | *JAK2*V617F | 10.2 | 13.7 | 721 | 184 | 0 | n.a. | n.a. | no |
| 33 | ET | 57 | m | *CALR* (Type 2) | 6.5 | 13.8 | 547 | 201 | 0 | n.a. | 1 | no |
| 34 | ET | 47 | m | *JAK2*V617F | 7.2 | 14.9 | 894 | 170 | 0 | n.a. | n.a. | no |
| 35 | ET | 61 | m | *JAK2*V617F | 10.6 | 16 | 825 | 210 | 0 | n.a. | 0 | no |
| 36 | ET | 68 | m | *CALR* (Type 1) | 7.73 | 14.7 | 555 | 138 | 0 | n.a. | n.a. | no |
| 37 | ET | 20 | f | *JAK2*V617F | 8.7 | 13.7 | 1269 | 155 | 0 | n.a. | n.a. | no |
| 38 | ET | 61 | f | *JAK2*V617F | 7.9 | 14.3 | 458 | 176 | 0 | n.a. | 0 | yes |
| 39 | PV | 73 | m | *JAK2*V617F | 20.52 | 12.5 | 893 | 224 | 0 | 1 | 1 | yes |
| 40 | PV | 73 | f | *JAK2*V617F | 22.96 | 11.8 | 186 | 257 | 1 | 2 | 1 | n.a. |
| 41 | PV | 67 | m | *JAK2*V617F | 26.52 | 12.9 | 577 | 225 | 0 | 1 | 0 | no |
| 42 | PV | 56 | f | *JAK2*V617F | 11.25 | 14.9 | 573 | 303 | 0 | 1 | 0 | no |
| 43 | PV | 75 | m | *JAK2*V617F | 21.91 | 14.3 | 440 | 169 | 0 | 1 | 0 | n.a. |
| 44 | PV | 96 | f | *JAK2*V617F | 74.32 | 11.3 | 437 | 361 | 1 | n.a. | n.a. | no |
| 45 | PV | 53 | f | *JAK2*V617F | 10.42 | 16.2 | 935 | 216 | 0 | 0.5 | 0 | no |
| 46 | PV | 43 | m | *JAK2* exon 12 | 12.62 | 15.8 | 516 | 170 | 0 | 2 | 0 | no |
| 47 | PV | 57 | f | *JAK2*V617F | 15.42 | 16.9 | 1269 | 246 | 0 | 0 | 0 | no |
| 48 | PV | 38 | m | *JAK2*V617F | 19.79 | 12.5 | 1467 | 257 | 0 | 1 | 0 | yes |
| **#** | **Diagnosis** | **Age** | **Gender** | **Driver mutation**  **Mutated Gene** | **WBC (G/L)** | **Hb (g/dL)** | **PLT (G/L)** | **LDH (U/L)** | **PB blasts (%)** | **BM blasts (%)** | **Grade of fibrosis** | **Splenomegaly** |
| 49 | PV | 63 | f | *JAK2*V617F | 27.4 | 15.2 | 198 | 302 | 0 | 0 | 0 | yes |
| 50 | PV | 27 | f | *JAK2*V617F | 8.06 | 15.9 | 647 | 408 | 0 | 2 | 0 | no |
| 51 | PV | 54 | m | *JAK2*V617F | 4.9 | 15.9 | 294 | 160 | 0 | 2 | 0 | no |
| 52 | PV | 69 | m | *JAK2*V617F | 12.37 | 16.4 | 764 | 235 | 0 | 1 | 0 | no |
| 53 | PV | 83 | f | *JAK2*V617F | 15.44 | 14.8 | 1171 | 286 | 0 | 1 | n.a. | no |
| 54 | PV | 46 | m | *JAK2*V617F | 13.73 | 17.5 | 404 | 273 | 0 | 1 | 0 | yes |
| 55 | PV | 44 | m | *JAK2*V617F | 10.38 | 16.6 | 706 | 263 | 0 | 1 | 0 | no |
| 56 | PV | 61 | f | *JAK2*V617F | 37.2 | 11 | 3176 | 457 | 0 | 1 | 1 | yes |
| 57 | PV | 47 | f | *JAK2*V617F | 10.2 | 14.1 | 483 | 211 | 0 | n.a. | n.a. | no |
| 58 | PV | 79 | f | *JAK2*V617F | 12.6 | 14 | 1136 | 218 | 0 | n.a. | n.a. | no |
| 59 | PV | 62 | f | *JAK2*V617F | 17.7 | 14.1 | 837 | 277 | 0 | 1 | 0 | no |
| 60 | PV | 75 | f | *JAK2*V617F | 15.5 | 14.4 | 482 | 228 | 0 | n.a. | 0 | no |
| 61 | PV | 67 | f | *JAK2*V617F | 10.4 | 15.2 | 352 | 171 | 0 | n.a. | n.a. | no |
| 62 | PV | 46 | m | *JAK2*V617F | 19.6 | 18.7 | 373 | 180 | 0 | n.a. | n.a. | no |
| 63 | post-ET MF | 70 | m | *CALR* (Type 2) | 3.68 | 9.7 | 500 | 493 | 0 | 1 | 1 | yes |
| 64 | post-PV MF | 74 | m | *JAK2*V617F | 13.26 | 14.6 | 511 | 268 | 0 | 10 | 1 | n.a. |
| 65 | post-PV MF | 57 | m | *JAK2*V617F | 32.86 | 12.8 | 321 | 671 | 1 | 1 | 2 | yes |
| 66 | post-ET MF | 60 | f | *JAK2*V617F | 23.8 | 10.8 | 507 | 366 | 0 | 1 | 1 | no |
| 67 | post-ET MF | 74 | f | *JAK2*V617F | 7.84 | 9.4 | 130 | 311 | 0 | 1 | 3 | yes |
| 68 | post-ET MF | 75 | m | *CALR* (Type 1) | 6.3 | 8.9 | 266 | 283 | 0 | 1 | 2 | n.a. |
| 69 | post-ET MF | 69 | f | *CALR* (Type 1) | 8.13 | 9.3 | 700 | 292 | 0 | 5 | 2 | n.a. |
| 70 | post-ET MF | 67 | m | *JAK2*V617F | 5.4 | 8.7 | 286 | 579 | 0 | 0 | 3 | yes |
| 71 | post ET MF | 58 | m | *CALR* (Type 2) | n.a. | n.a. | n.a. | 484 | 0 | 1 | 2 | yes |
| 72 | post-ET MF | 54 | m | *CALR* (Type 1) | 6.6 | 13.3 | 365 | 669 | 0 | n.a. | 2 | yes |
| 73 | post-PV MF | 77 | f | *JAK2*V617F | 15.3 | 12 | 212 | 268 | 7 | 8.5 | 3 | yes |
| 74 | post-ET MF | 65 | m | *CALR* (Type 1) | 6.7 | 10.9 | 217 | 704 | 2 | 1 | 3 | no |
| **#** | **Diagnosis** | **Age** | **Gender** | **Driver mutation**  **Mutated Gene** | **WBC (G/L)** | **Hb (g/dL)** | **PLT (G/L)** | **LDH (U/L)** | **PB blasts (%)** | **BM blasts (%)** | **Grade of fibrosis** | **Splenomegaly** |
| 75 | post-ET MF | 57 | f | *JAK2*V617F | 9.4 | 9.7 | 302 | 786 | 0 | 1 | 2 | yes |
| 76 | post-ET MF | 60 | f | *JAK2*V617F | 7.9 | 10.9 | 340 | 407 | 0 | 1 | 2 | yes |
| 77 | post-ET MF | 67 | f | *JAK2*V617F | 42.28 | 11.5 | 302 | 557 | 0 | 1 | 1 | no |
| 78 | post-PV MF | 79 | f | *JAK2*V617F | 41.8 | 9.9 | 8 | 402 | 0 | 1.5 | 1 | yes |
| 79 | post-ET MF | 88 | m | *JAK2*V617F | 26.1 | 9.5 | 187 | 746 | 0 | 1.5 | 1 | yes |
| 80 | post-PV MF | 61 | f | *JAK2*V617F | 29.7 | 14.4 | 653 | 696 | 0 | n.a. | 1 | yes |
| 81 | post ET MF | 79 | f | *CALR* (Type 1) | 5.3 | 9.5 | 336 | 227 | 0 | n.a. | 2 | no |
| 82 | post PV MF | 56 | f | *JAK2*V617F | 12.3 | 15.9 | 100 | 283 | 0 | 0.5 | 1 | n.a. |
| 83 | post PV MF | 72 | m | *JAK2*V617F | 15.3 | 9.9 | 36 | 680 | 0 | 10 | 2 | yes |
| 84 | post ET MF | 55 | f | *JAK2*V617F | 8.2 | 14.2 | 1169 | 297 | 0 | n.a. | n.a. | n.a. |
| 85 | PMF | 71 | m | *JAK2*V617F | 3.92 | 7.6 | 98 | 222 | 0 | 2 | 3 | yes |
| 86 | PMF | 77 | f | *JAK2*V617F | 9.93 | 9.4 | 367 | 827 | 1 | 1 | 2 | yes |
| 87 | PMF | 68 | f | *JAK2*V617F | 21.77 | 9.2 | 176 | 642 | 1 | 1 | n.a. | n.a. |
| 88 | PMF | 75 | f | *JAK2*V617F | 31.62 | 12.9 | 441 | 412 | 0 | 1 | 2 | yes |
| 89 | PMF | 58 | f | *JAK2*V617F | 2.98 | 10.9 | 88 | 1259 | 1 | 1 | 3 | yes |
| 90 | PMF | 47 | m | *CALR* (Type 1) | 5.6 | 12.2 | 165 | 952 | 0 | 3 | 3 | yes |
| 91 | PMF | 60 | m | *CALR* (Type 1) | 7.42 | 12.3 | 175 | 537 | 2 | 1 | 2 | yes |
| 92 | PMF | 56 | m | *CALR* (Type 1) | 8.58 | 13.9 | 650 | 395 | 0 | 2 | 0 | no |
| 93 | PMF | 45 | m | *CALR* (Type 1) | 6.16 | 14.4 | 372 | 415 | 1 | 2 | 1-2 | yes |
| 94 | PMF | 28 | f | *CALR* (Type 1) | 8 | 10.9 | 189 | 810 | 0 | n.a | 3 | yes |
| 95 | PMF | 65 | m | *JAK2*V617F | 2.8 | 8.5 | 58 | 563 | 1 | n.a | 3 | no |
| 96 | PMF | 71 | m | *JAK2*V617F | 9.1 | 11 | 161 | 367 | 0 | 1 | 2 | yes |
| 97 | PMF | 62 | f | *JAK2*V617F | 22.6 | 15.5 | 520 | 438 | 0 | 0 | 1 | yes |
| 98 | PMF | 71 | f | *CALR* (Type 2) | 38.79 | 9.8 | 153 | 873 | 11 | 10 | 2 | yes |
| 99 | PMF | 80 | m | *CALR* (Type 1) | 20.08 | 8.5 | 654 | 694 | 0 | 2 | 3 | no |
| 100 | PMF | 45 | m | *JAK2*V617F | 11.93 | 17 | 396 | 303 | 0 | 1 | 0 | yes |
| **#** | **Diagnosis** | **Age** | **Gender** | **Driver mutation**  **Mutated Gene** | **WBC (G/L)** | **Hb (g/dL)** | **PLT (G/L)** | **LDH (U/L)** | **PB blasts (%)** | **BM blasts (%)** | **Grade of fibrosis** | **Splenomegaly** |
| 101 | PMF | 67 | f | *JAK2*V617F | 7.46 | 14.9 | 415 | 304 | 0 | 1 | 0 | no |
| 102 | PMF | 48 | f | *CALR* (Type 1) | 8.27 | 10.9 | 487 | 350 | 0 | 0.5 | 2 | n.a. |
| 103 | PMF | 79 | m | *CALR* (Type 2) | 24.1 | 9.2 | 1759 | 959 | 11 | 4.5 | 3 | yes |
| 104 | PMF | 64 | m | *JAK2*V617F | 60 | 13.2 | 168 | 884 | 2 | 2 | 2 | yes |
| 105 | PMF | 80 | m | *CALR* (Type 1) | 17.1 | 9.8 | 138 | 656 | 2 | 3 | 3 | yes |
| 106 | PMF | 76 | m | *JAK2*V617F | 1.7 | 6.9 | 91 | 679 | 0 | n.a. | 1 | yes |
| 107 | PMF | 57 | m | *JAK2*V617F | 19.1 | 11.7 | 145 | 543 | 2 | 3 | 2-3 | yes |
| 108 | PMF | 80 | m | *CALR* (Type 2) | 3.46 | 10.1 | 345 | 311 | 0 | 5 | 2 | yes |
| 109 | PMF | 78 | m | *JAK2*V617F | 36.6 | 8.6 | 92 | 1310 | 2 | 3 | 1 | yes |
| 110 | PMF | 29 | m | *CALR* (Type 2) | 7.7 | 13.4 | 1611 | 270 | 0 | n.a. | 1 | yes |
| 111 | PMF | 89 | m | *JAK2*V617F | 7.3 | 9.5 | 414 | 830 | 0 | n.a. | n.a. | yes |
| 112 | post-MPN sAML | 55 | f | n.a. | 24.8 | 14.1 | 87 | 351 | 7 | 30 | 0 | no |
| 113 | post-PV-MF sAML | 77 | m | *JAK2*V617F | 4.7 | 10 | 68 | 975 | 20 | 10 | 2 | no |
| 114 | post-PV sAML | 80 | m | *JAK2*V617F | 4.22 | 9.8 | 223 | 483 | 31 | 26 | 1 | yes |
| 115 | post-PV-MF sAML | 67 | m | *JAK2*V617F | 3.59 | 9.1 | 52 | 865 | 25 | 7 | 2 | yes |
| 116 | post-PV-MF sAML | 88 | f | *CALR* (Type 1) | 26 | 8.4 | 72 | 2098 | 21 | 26 | 3 | yes |
| 117 | post-PMF sAML | 76 | m | *JAK2*V617F | 5.07 | 8.1 | 9 | 371 | 24 | 40 | 2 | yes |
| 118 | post-PV sAML | 78 | m | *JAK2*V617F | 4.6 | 8.5 | 24 | 683 | 45 | 62 | 3 | yes |
| 119 | post-PV-MF sAML* | 55 | f | *JAK2*V617F | 3.29 | 9 | 18 | 692 | 2 | 6 | 3 | yes |
| 120 | post-PMF sAML | 61 | f | *JAK2*V617F | 2.21 | 5.2 | 17 | 427 | 24 | 50 | 3 | yes |
| 121 | post-ET sAML | 82 | f | *JAK2*V617F | 2.5 | 8.3 | 90 | 281 | n.a. | 30 | 1 | yes |
| 122 | post-PMF sAML* | 70 | f | *MPL* | 21.3 | 9.7 | 291 | 961 | 7 | 7 | 2-3 | yes |
| 123 | post-PV-sAML | 74 | f | *JAK2*V617F | 16.1 | 661 | 281 | 352 | 8.7 | 60 | 1 | no |

Abbreviations: BM blasts, percentage of identified CD34+ blasts in Giemsa-stained bone marrow smears or bone marrow histology sections; ET, essential thrombocythemia; F, female; Hb, hemoglobin concentration; LDH, lactate dehydrogenase; M, male; MF, myelofibrosis; n.a., data not available (analysis not performed or inconclusive); PB blasts, percentage of identified CD34+ blasts in the peripheral blood; PLT, platelet count; PMF, primary myelofibrosis; PV, polycythemia vera; sAML, secondary acute myeloid leukemia following MPN; WBC, white blood count. *, patients with incipient sAML (confirmed during follow up).

**Table S3**

**Summary of engraftment results in NSGS mice**

| **Donor** | **Diagnosis** | **Injected fraction** | **Injected cell number per mouse** | | **Engraftment (% human CD45^+^ cells) in mouse BM** |
| --- | --- | --- | --- | --- | --- |
| **Bulk MNC vs CD34**^−^ **MNC** | | | | | |
| #29 | ET | Bulk MNC (n=5) | 0.5 x 10^6^ | | 6.6 ± 6.7 % |
|  |  | CD34^−^ MNC (n=5) | 0.4 x 10^6^ | | 0.5 ± 0.4 % |
| #43 | PV | Bulk MNC (n=5) | 0.6 x 10^6^ | | 14.8 ± 4 % |
|  |  | CD34^−^ MNC (n=5) | 0.3 x 10^6^ | | 0.1 ± 0.1 % |
| #44 | PV | Bulk MNC (n=5) | 0.5 x 10^6^ | | 17.2 ± 16.6 % |
|  |  | CD34^−^ MNC (n=5) | 0.4 x 10^6^ | | 0.1 ± 0.03 % |
| **Bulk MNC vs CD38^+^ MNC vs CD38**^−^ **MNC** | | | | | |
| #43 | PV | Bulk MNC (n=4) | 0.6 x 10^6^ | | 7.7 ± 4.8 % |
|  |  | CD38^+^ MNC (n=4) | 0.2 x 10^6^ | | 0.3 ± 0.2 % |
|  |  | CD38^−^ MNC (n=4) | 0.2 x 10^6^ | | 13.8 ± 11.2 % |
| #88 | PMF | Bulk MNC (n=5) | 0.5 x 10^6^ | | 8.9 ± 9.9 % |
|  |  | CD38^+^ MNC (n=5) | 0.2 x 10^6^ | | 1.2 ± 2.6 % |
|  |  | CD38^−^ MNC (n=5) | 0.2 x 10^6^ | | 1.7 ± 2.8 % |
| #104 | PMF | Bulk MNC (n=5) | 0.2 x 10^6^ | | 0.9 ± 0.8 % |
|  |  | CD38^+^ MNC (n=5) | 0.2 x 10^6^ | | 0.05 ± 0.03 % |
|  |  | CD38^−^ MNC (n=5) | 0.2 x 10^6^ | | 0.7 ± 1.3 % |
| **Bulk MNC vs CD34**^+^/**CD38^+^ MNC vs CD34**^+^/**CD38**^−^ **MNC** | | | | | |
| #104 | PMF | Bulk MNC (n=4) | | 0.5 x 10^6^ | 7.8 ± 4.4 % |
|  |  | CD34^+^/CD38^+^ MNC (n=5) | | 0.4 x 10^6^ | 0.06 ± 0.03 % |
|  |  | CD34^+^/CD38^−^ MNC (n=4) | | 0.3 x 10^6^ | 3.1 ± 2 % |
| #114 | post-PV sAML | Bulk MNC (n=5) | | 0.5 x 10^6^ | 88.4 ± 6.8 % |
|  |  | CD34^+^/CD38^+^ MNC (n=5) | | 0.5 x 10^6^ | 81.4 ± 7.9 % |
|  |  | CD34^+^/CD38^−^ MNC (n=5) | | 0.5 x 10^6^ | 52.3 ± 31.2 % |

| **Donor** | **Diagnosis** | **Injected cell number per mouse** | **Engraftment (% human CD45^+^ cells) in mouse BM** |
| --- | --- | --- | --- |
| **Limiting Dilution Experiments (CD34^+^ MNC)** | | | |
| #43 | PV | 5000 CD34^+^ cells (n=5) | 9.6 ± 6.1 % |
|  |  | 500 CD34^+^ cells (n=5) | 2.1 ± 1.3 % |
|  |  | 50 CD34^+^ cells (n=5) | 0.1 ± 0.1 % |
| #88 | PMF | 5000 CD34^+^ cells (n=4) | 4 ± 2.2 % |
|  |  | 500 CD34^+^ cells (n=4) | 0.4 ± 0.2 % |
|  |  | 50 CD34^+^ cells (n=4) | 0.4 ± 0.7 % |

Mononuclear cells obtained from the bone marrow or peripheral blood of patients with MPN (ET, n=1; PV, n=2; PMF, n=2) or post-MPN sAML (n=1) were T cell depleted and/or sorted to purify stem and progenitor cell subsets as described in the text. Patient numbers (#) refer to cases shown in Table S2. Experiments were prepared to compare engraftment levels in i) NSGS mice in T cell-depleted (CD3-negative) bulk MNC versus (vs) purified CD34^–^ (stem/progenitor-depleted) MNC, ii) bulk MNC vs CD38^+^ (progenitor-containing) MNC vs CD38^−^ (progenitor-depleted) MNC, iii) bulk MNC vs CD34^+^/CD38^+^ (progenitor-purified) MNC vs CD34^+^/CD38^−^ (stem cell-enriched) MNC, or iv) various numbers of CD34^+^ MPN cells (dilution experiments). MPN cell fractions were injected intravenously into sublethally irradiated NSGS mice. After 8-30 weeks, mice were sacrificed and the levels of engraftment of human cells in NSGS mouse bone marrow samples was analyzed by multicolor flow cytometry (Figure S2). The table shows a summary of these experiments including number of mice injected, number of cells injected and engraftment levels (expressed as mean±SD of human CD45^+^ cells in mouse bone marrow). Abbreviations: BM, bone marrow; ET, essential thrombocythemia; MNC, mononuclear cells; PMF, primary myelofibrosis; PV, polycythemia vera; sAML, secondary acute myeloid leukemia following MPN; SD, standard deviation.

**Table S4**

**Calculated frequencies of NSGS-engrafting MPN NSC among CD34^+^ cells**

|  | | **Detected engraftment**  **in NSGS mice injected with** | | | **Calculated NSC frequency among all CD34^+^ cells** |
| --- | --- | --- | --- | --- | --- |
| **Donor** | **Diagnosis** | **5000**  **CD34^+^ cells** | **500**  **CD34^+^ cells** | **50**  **CD34^+^ cells** | **% (95% confidence interval)** |
| #43 | PV | 5/5 | 4/5 | 2/5 | 0.4% (0.2% - 1.2%) |
| #88 | PMF | 4/4 | 4/4 | 2/4 | 1.4% (0.4% - 5.4%) |
| Both patients | | 9/9 | 8/9 | 4/9 | 0.6% (0.3% - 1.3%) |

Various numbers (5000, 500, or 50) of purified (sorted) CD34^+^ bone marrow (BM) derived mononuclear cells obtained from patients with MPN were injected intravenously into sublethally irradiated NSGS mice. For patients´ characteristics (#43 and #88) see Table S2. After 28-30 weeks, mice were sacrificed and engraftment of human cells in NSGS BM was evaluated by multicolor flow cytometry (Figure S2). The table shows the rate of engraftment (n/n mice) produced by CD34^+^ cells in each donor and the calculated frequencies of NSC (with the 95% confidence interval) within all CD34^+^ cells in each experiment. Calculations were performed using extreme limiting dilution analysis.^8^ Abbreviations: MPN, myeloproliferative neoplasm; NSC, neoplastic stem cells; PMF, primary myelofibrosis; PV, polycythemia vera.

**Table S5**

**Phenotype of CD34^+^/CD38^−^ stem cells in MPN and comparison to stem cells in secondary acute myeloid leukemia (sAML) and in the normal bone marrow (nBM)**

| Antigen | CD | Expression of cell surface antigens on  CD34^+^/CD38^−^ stem cells | | | | |
| --- | --- | --- | --- | --- | --- | --- |
|  |  | nBM | ET | PV | MF | sAML |
|  | | | | | | |
| IL-2RA | CD25 | − | − | − | + | + |
| DPPIV | CD26 | − | − | − | +/− | − |
| T44 | CD28 | − | − | − | − | − |
| Siglec-3 | CD33 | ++ | + | + | + | ++ |
| Hermes | CD44 | +++ | ++ | ++ | ++ | + |
| IAP | CD47 | ++ | ++ | ++ | ++ | ++ |
| Campath-1 | CD52 | + | + | +/− | + | +/− |
| B7-1 | CD80 | − | − | − | − | − |
| HB15 | CD83 | + | +/− | − | − | − |
| B7-2 | CD86 | − | − | − | − | − |
| Thy-1 | CD90 | +/− | − | +/− | +/− | − |
| C1qR1 | CD93 | +/− | +/− | +/− | + | +/− |
| Tactile | CD96 | − | − | − | +/− | +/− |
| ADGRE5 | CD97 | ++ | ++ | ++ | ++ | ++ |
| MIC2 | CD99 | ++ | +++ | +++ | +++ | +++ |
| Endoglin | CD105 | + | + | + | + | + |
| TPOR | CD110 | +/− | +/− | +/− | +/− | +/− |
| G-CSFR | CD114 | − | − | − | − | − |
| M-CSFR | CD115 | − | − | − | − | − |
| KIT | CD117 | ++ | ++ | ++ | ++ | ++ |
| IL-3RA | CD123 | + | + | + | + | + |
| CSF2RB | CD131 | − | − | − | − | − |
| Prominin-1 | CD133 | ++ | + | + | ++ | ++ |
| FLT3 | CD135 | +/− | +/− | +/− | − | +/− |
| CXCR4 | CD184 | − | +/− | +/− | + | +/− |
| MRC1 | CD206 | − | +/− | +/− | +/− | +/− |
| IGF-1R | CD221 | +/− | +/− | +/− | +/− | +/− |
| MDR-1 | CD243 | + | + | + | + | ++ |
| NGF-R | CD271 | − | − | − | − | − |
| PD-L2 | CD273 | − | − | − | − | − |
| PD-L1 | CD274 | + | + | + | + | + |
| PD-1 | CD279 | − | − | − | − | − |
| VEGFR-2 | CD309 | − | − | − | − | − |
| TIM-3 | CD366 | − | − | − | − | − |
| CLL-1 | CD371 | − | − | − | − | +/− |
| IL-1RAP | n.c. | − | − | − | +/− | − |
| EPOR | n.c. | +/− | − | − | − | − |
| MET | n.c. | − | − | − | − | − |
| OSMRb | n.c. | − | − | − | − | − |

The table shows a summary of staining results obtained in all patients with MPN, sAML and nBM samples tested. Expression of surface antigens on CD34^+^/CD38^−^ stem cells was examined by multicolor flow cytometry as described in the text and in the supplement. Expression levels were calculated as staining index (SI, median fluorescence intensity of the indicated marker divided by the median fluorescence intensity of the isotype control). A minimum of three samples from each donor-group were tested. Results were calculated as mean SI from all patients in each cohort and graded using the following score: −, SI < 1.5; +/−, SI = 1.5-3; +, SI = 3.1-10; ++, SI = 10.1-100; +++, SI > 100.

Abbreviations: C1qR1, complement C1q receptor; CD, cluster of differentiation; CLL-1, C-type lectin-like molecule-1; CSF2RB, colony-stimulating factor 2 receptor-beta; CXCR4, chemokine C-X-C motif receptor 4; DPPIV, dipeptidyl peptidase IV; EPOR, erythropoietin receptor; ET, essential thrombocythemia; FLT3, FMS-like tyrosine kinase 3; G-CSFR, granulocyte colony-stimulating factor receptor; IAP, integrin associated protein; IGF-1R, insulin-like growth factor 1 receptor; IL-1RAP, interleukin-1 receptor accessory protein; IL-2RA, interleukin-2 receptor alpha chain; IL-3RA, interleukin-3 receptor alpha chain; M-CSFR, macrophage colony-stimulating factor receptor; MDR-1, multidrug resistance protein 1; MF, myelofibrosis; MPN, myeloproliferative neoplasm; MRC1, mannose receptor C-type 1; nBM, normal bone marrow; NGF-R, nerve growth factor receptor; n.c., not (yet) clustered; OSMRb, oncostatin M receptor beta; PD-1, programmed death protein 1; PD-L1, programmed death ligand 1; PD-L2, programmed death ligand 2; PV, polycythemia vera; sAML, secondary acute myeloid leukemia following MPN; TIM-3, T cell immunoglobulin and mucin domain-containing protein 3; TPOR, thrombopoietin receptor; VEGFR-2, vascular endothelial growth factor receptor 2.

**Table S6**

**Phenotype of CD34^+^/CD38^−^ MPN NSC and comparison between patients suffering from post-ET/PV MF and PMF**

| Antigen | CD | Expression of cell surface antigens on  CD34^+^/CD38^−^ stem cells | | | |
| --- | --- | --- | --- | --- | --- |
|  |  | post-ET/PV MF | | PMF | |
|  |  | |  | |  |
| IL-2RA | CD25 | | + | | +/− |
| DPPIV | CD26 | | +/− | | +/− |
| T44 | CD28 | | − | | − |
| Siglec-3 | CD33 | | + | | ++ |
| Hermes | CD44 | | ++ | | ++ |
| IAP | CD47 | | ++ | | ++ |
| Campath-1 | CD52 | | + | | +/− |
| B7-1 | CD80 | | − | | − |
| HB15 | CD83 | | − | | +/− |
| B7-2 | CD86 | | − | | − |
| Thy-1 | CD90 | | +/− | | − |
| C1qR1 | CD93 | | + | | + |
| Tactile | CD96 | | +/− | | +/− |
| ADGRE5 | CD97 | | ++ | | ++ |
| MIC2 | CD99 | | +++ | | +++ |
| Endoglin | CD105 | | + | | + |
| TPOR | CD110 | | +/− | | +/− |
| G-CSFR | CD114 | | − | | − |
| M-CSFR | CD115 | | − | | − |
| KIT | CD117 | | ++ | | ++ |
| IL-3RA | CD123 | | + | | + |
| CSF2RB | CD131 | | − | | − |
| Prominin-1 | CD133 | | ++ | | ++ |
| FLT3 | CD135 | | − | | +/− |
| CXCR4 | CD184 | | + | | + |
| MRC1 | CD206 | | +/− | | +/− |
| IGF-1R | CD221 | | + | | +/− |
| MDR-1 | CD243 | | + | | + |
| NGF-R | CD271 | | − | | − |
| PD-L2 | CD273 | | − | | − |
| PD-L1 | CD274 | | + | | + |
| PD-1 | CD279 | | − | | − |
| VEGFR-2 | CD309 | | − | | − |
| TIM-3 | CD366 | | − | | − |
| CLL-1 | CD371 | | − | | − |
| IL-1RAP | n.c. | | − | | +/− |
| EPOR | n.c. | | − | | − |
| MET | n.c. | | − | | − |
| OSMRb | n.c. | | − | | − |

The table shows a summary of staining results obtained in all patients with post-ET/PV MF and PMF samples tested. Expression of surface antigens on CD34^+^/CD38^−^ stem cells was examined by multicolor flow cytometry as described in the text and in the supplement. Expression levels were calculated as staining index (SI, median fluorescence intensity of the indicated marker divided by the median fluorescence intensity of the isotype control). A minimum of three samples from each donor-group were tested. Results were calculated as mean SI from all patients in each cohort and graded using the following score: −, SI < 1.5; +/−, SI = 1.5-3; +, SI = 3.1-10; ++, SI = 10.1-100; +++, SI > 100.

Abbreviations: C1qR1, complement C1q receptor; CD, cluster of differentiation; CLL-1, C-type lectin-like molecule-1; CSF2RB, colony-stimulating factor 2 receptor-beta; CXCR4, chemokine C-X-C motif receptor 4; DPPIV, dipeptidyl peptidase IV; EPOR, erythropoietin receptor; ET, essential thrombocythemia; FLT3, FMS-like tyrosine kinase 3; G-CSFR, granulocyte colony-stimulating factor receptor; IAP, integrin associated protein; IGF-1R, insulin-like growth factor 1 receptor; IL-1RAP, interleukin-1 receptor accessory protein; IL-2RA, interleukin-2 receptor alpha chain; IL-3RA, interleukin-3 receptor alpha chain; M-CSFR, macrophage colony-stimulating factor receptor; MDR-1, multidrug resistance protein 1; MF, myelofibrosis; MPN, myeloproliferative neoplasm; MRC1, mannose receptor C-type 1; n.c., not (yet) clustered; NGF-R, nerve growth factor receptor; OSMRb, oncostatin M receptor beta; PD-1, programmed death protein 1; PD-L1, programmed death ligand 1; PD-L2, programmed death ligand 2; PMF, primary myelofibrosis; PV, polycythemia vera; TIM-3, T cell immunoglobulin and mucin domain-containing protein 3; TPOR, thrombopoietin receptor; VEGFR-2, vascular endothelial growth factor receptor 2.

## Table S7

## Phenotype of CD34^+^/CD38^+^ MPN progenitor cells and comparison to secondary acute myeloid leukemia (sAML) and normal bone marrow (nBM) progenitor cells

| Antigen | CD | Expression of cell surface antigens on  CD34^+^/CD38^+^ progenitor cells | | | | |
| --- | --- | --- | --- | --- | --- | --- |
|  |  | nBM | ET | PV | MF | sAML |
|  | | | | | | |
| IL-2RA | CD25 | − | − | − | − | +/− |
| DPPIV | CD26 | − | − | − | − | − |
| T44 | CD28 | − | − | − | − | − |
| Siglec-3 | CD33 | ++ | ++ | ++ | ++ | ++ |
| Hermes | CD44 | +++ | ++ | ++ | ++ | ++ |
| IAP | CD47 | ++ | ++ | ++ | ++ | ++ |
| Campath-1 | CD52 | + | +/− | +/− | +/− | +/− |
| B7-1 | CD80 | − | − | − | − | − |
| HB15 | CD83 | + | +/− | − | +/− | − |
| B7-2 | CD86 | − | − | − | +/− | − |
| Thy-1 | CD90 | +/− | − | − | − | − |
| C1qR1 | CD93 | +/− | +/− | +/− | +/− | +/− |
| Tactile | CD96 | − | − | − | − | +/− |
| ADGRE5 | CD97 | ++ | ++ | ++ | ++ | ++ |
| MIC2 | CD99 | ++ | +++ | +++ | +++ | +++ |
| Endoglin | CD105 | + | + | + | + | + |
| TPOR | CD110 | +/− | +/− | +/− | + | +/− |
| G-CSFR | CD114 | − | − | − | − | − |
| M-CSFR | CD115 | − | − | − | − | − |
| KIT | CD117 | ++ | ++ | ++ | ++ | ++ |
| IL-3RA | CD123 | + | + | + | + | ++ |
| CSF2RB | CD131 | − | − | − | − | − |
| Prominin-1 | CD133 | ++ | +/− | + | + | + |
| FLT3 | CD135 | +/− | +/− | +/− | − | +/− |
| CXCR4 | CD184 | − | + | + | + | +/− |
| MRC1 | CD206 | − | +/− | +/− | +/− | +/− |
| IGF-1R | CD221 | +/− | +/− | +/− | +/− | +/− |
| MDR-1 | CD243 | + | + | + | + | ++ |
| NGF-R | CD271 | − | − | − | − | − |
| PD-L2 | CD273 | − | − | − | − | − |
| PD-L1 | CD274 | + | + | + | + | + |
| PD-1 | CD279 | − | − | − | − | − |
| VEGFR-2 | CD309 | − | − | − | − | − |
| TIM-3 | CD366 | − | +/− | +/− | − | − |
| CLL-1 | CD371 | − | ++ | ++ | + | + |
| IL-1RAP | n.c. | − | +/− | +/− | +/− | +/− |
| EPOR | n.c. | +/− | − | − | − | − |
| MET | n.c. | − | − | − | − | − |
| OSMRb | n.c. | − | − | − | − | − |

The table shows a summary of staining results obtained in all patients with MPN, sAML and nBM samples tested. Expression of surface antigens on CD34^+^/CD38^+^ progenitor cells was examined by multicolor flow cytometry as described in the text and in the supplement. Expression levels were calculated as staining index (SI, median fluorescence intensity of the indicated marker divided by the median fluorescence intensity of the isotype control). A minimum of three samples from each donor-group were tested. Results were calculated as mean SI from all patients in each cohort and graded using the following score: −, SI < 1.5; +/−, SI = 1.5-3; +, SI = 3.1-10; ++, SI = 10.1-100; +++, SI > 100.

Abbreviations: C1qR1, complement C1q receptor; CD, cluster of differentiation; CLL-1, C-type lectin-like molecule-1; CSF2RB, colony-stimulating factor 2 receptor-beta; CXCR4, chemokine C-X-C motif receptor 4; DPPIV, dipeptidyl peptidase IV; EPOR, erythropoietin receptor; ET, essential thrombocythemia; FLT3, FMS-like tyrosine kinase 3; G-CSFR, granulocyte colony-stimulating factor receptor; IAP, integrin associated protein; IGF-1R, insulin-like growth factor 1 receptor; IL-1RAP, interleukin-1 receptor accessory protein; IL-2RA, interleukin-2 receptor alpha chain; IL-3RA, interleukin-3 receptor alpha chain; M-CSFR, macrophage colony-stimulating factor receptor; MDR-1, multidrug resistance protein 1; MF, myelofibrosis; MPN, myeloproliferative neoplasm; MRC1, mannose receptor C-type 1; nBM, normal bone marrow; n.c., not (yet) clustered; NGF-R, nerve growth factor receptor; OSMRb, oncostatin M receptor beta; PD-1, programmed death protein 1; PD-L1, programmed death ligand 1; PD-L2, programmed death ligand 2; PV, polycythemia vera; sAML, secondary acute myeloid leukemia following MPN; TIM-3, T cell immunoglobulin and mucin domain-containing protein 3; TPOR, thrombopoietin receptor; VEGFR-2, vascular endothelial growth factor receptor 2.

## Table S8

## Expression of markers and targets on MPN-related cell lines

| Antigen | CD | Expression of cell surface antigens on | | | | | | |
| --- | --- | --- | --- | --- | --- | --- | --- | --- |
|  |  | HEL | SET-2 | UT-7 WT | | UT-7 B5 | UT-7 C4 | UT-7 E2 |
| IL-2RA | CD25 | − | − | − | − | | − | − |
| DPPIV | CD26 | − | − | − | − | | − | − |
| T44 | CD28 | − | − | − | − | | − | − |
| Siglec-3 | CD33 | ++ | ++ | ++ | ++ | | ++ | ++ |
| Hermes | CD44 | ++ | ++ | ++ | +++ | | ++ | ++ |
| IAP | CD47 | ++ | ++ | ++ | +++ | | ++ | +++ |
| Campath-1 | CD52 | + | +/− | ++ | + | | + | ++ |
| B7-1 | CD80 | +/− | +/− | − | − | | − | +/− |
| HB15 | CD83 | +/− | +/− | − | − | | +/− | − |
| B7-2 | CD86 | +/− | − | − | − | | − | − |
| Thy-1 | CD90 | + | − | ++ | +/− | | ++ | +/− |
| C1qR1 | CD93 | +/− | + | +/− | +/− | | + | +/− |
| Tactile | CD96 | +/− | + | + | + | | + | + |
| ADGRE5 | CD97 | + | + | ++ | ++ | | ++ | ++ |
| MIC2 | CD99 | ++ | ++ | ++ | ++ | | ++ | ++ |
| Endoglin | CD105 | + | ++ | ++ | ++ | | ++ | ++ |
| TPOR | CD110 | + | + | ++ | + | | + | + |
| G-CSFR | CD114 | +/− | +/− | − | − | | − | − |
| M-CSFR | CD115 | +/− | − | + | + | | + | + |
| KIT | CD117 | + | + | ++ | + | | + | + |
| IL-3RA | CD123 | +/− | + | + | + | | + | + |
| CSF2RB | CD131 | − | + | − | − | | − | − |
| Prominin-1 | CD133 | − | − | +/− | − | | +/− | − |
| FLT3 | CD135 | +/− | − | − | +/− | | − | − |
| CXCR4 | CD184 | + | + | +/− | +/− | | +/− | +/− |
| MRC1 | CD206 | + | + | + | +/− | | + | +/− |
| IGF-1R | CD221 | ++ | +/− | ++ | + | | + | + |
| MDR-1 | CD243 | ++ | ++ | ++ | ++ | | ++ | ++ |
| NGF-R | CD271 | − | − | − | − | | − | − |
| PD-L2 | CD273 | − | − | − | − | | − | − |
| PD-L1 | CD274 | ++ | + | + | + | | + | + |
| PD-1 | CD279 | +/− | − | − | − | | − | − |
| VEGFR-2 | CD309 | +/− | +/− | +/− | +/− | | +/− | +/− |
| TIM-3 | CD366 | +/− | +/− | − | +/− | | +/− | − |
| CLL-1 | CD371 | − | +/− | +/− | +/− | | − | +/− |
| IL-1RAP | n.c. | +/− | − | + | + | | + | + |
| EPOR | n.c. | − | − | +/− | − | | − | − |
| MET | n.c. | − | − | +/− | +/− | | +/− | +/− |
| OSMRb | n.c. | − | +/− | − | − | | − | − |

The table shows a summary of all multicolor flow cytometry staining results obtained in all MPN-related cell lines. Results are expressed as staining index (SI, median fluorescence intensity of the indicated marker divided by the median fluorescence intensity of the isotype control) and indicate the mean SI of at least three independent experiments. SI values were graded using the following scoring system: −, SI < 1.5; +/−, SI = 1.5-3; +, SI = 3.1-10; ++, SI = 10.1-100; +++, SI > 100.

Abbreviations: B5, UT-7 cells expressing del61/del25 *CALR* mutation; C1qR1, complement C1q receptor; C4, UT-7 cells expressing del61/WT *CALR* mutation; CD, cluster of differentiation; CLL-1, C-type lectin-like molecule-1; CSF2RB, colony-stimulating factor 2 receptor-beta; CXCR4, chemokine C-X-C motif receptor 4; DPPIV, dipeptidyl peptidase IV; E2, UT-7 cells expressing del58/WT *CALR* mutation; EPOR, erythropoietin receptor; FLT3, FMS-like tyrosine kinase 3; G-CSFR, granulocyte colony-stimulating factor receptor; IAP, integrin associated protein; IGF-1R, insulin-like growth factor 1 receptor; IL-1RAP, interleukin-1 receptor accessory protein; IL-2RA, interleukin-2 receptor alpha chain; IL-3RA, interleukin-3 receptor alpha chain; M-CSFR, macrophage colony-stimulating factor receptor; MDR-1, multidrug resistance protein 1; MPN, myeloproliferative neoplasm; MRC1, mannose receptor C-type 1; n.c., not (yet) clustered; NGF-R, nerve growth factor receptor; OSMRb, oncostatin M receptor beta; PD-1, programmed death protein 1; PD-L1, programmed death ligand 1; PD-L2, programmed death ligand 2; TIM-3, T cell immunoglobulin and mucin domain-containing protein 3; TPOR, thrombopoietin receptor; VEGFR-2, vascular endothelial growth factor receptor 2; WT, UT-7 cells expressing *CALR* wild type.

## Table S9

## Effects of anti-neoplastic drugs on proliferation of MPN cells

| Cells | ^3^H-thymidine uptake - IC_50_ values | | | | | | | |
| --- | --- | --- | --- | --- | --- | --- | --- | --- |
|  | Ruxolitinib  [µM] | Fedratinib  [µM] | Avapritinib  [µM] | Midostaurin  [µM] | Pelabresib  [µM] | JQ1  [µM] | dBET6  [µM] | GO  [µg/ml] |
| Primary MNC | 10 | 0.2 | 4 | 0.26 | 0.9 | 0.2 | 0.01 | 0.3 |
| HEL | 10 | 1.5 | 3.7 | 0.8 | 1.5 | 0.2 | 0.1 | 37.4 |
| SET-2 | 0.1 | 0.3 | 8.6 | 0.4 | 1.9 | 0.2 | 0.01 | 63.2 |
| UT-7 WT | 0.6 | 3.6 | 4.7 | 0.7 | 1.5 | 0.4 | 0.5 | n.t. |
| UT-7 B5 | 0.3 | 4.7 | 4.7 | 0.5 | 3 | 0.7 | 0.4 | n.t. |

MPN-related cell lines (HEL, SET-2, UT-7 cells expressing wild type *CALR* (WT) or *CALR* del61/del25 mutation (B5)) or MNC isolated from the bone marrow or peripheral blood of patients with MPN (ET, n=5; PV, n=4; MF, n=4) were incubated with increasing concentrations of targeted drugs (as indicated) at 37°C for 48 hours. Thereafter, proliferation was measured by quantifying ^3^H-thymidine uptake. Technical details are described in the text in this supplement. The table shows the IC_50_ values obtained for each compound in the cell lines or in the primary MNC samples. Abbreviations: ET, essential thrombocythemia; GO, gemtuzumab-ozogamicin; IC_50_, half maximal inhibitory concentration; MF, myelofibrosis; MNC, mononuclear cells; MPN, myeloproliferative neoplasms; n.t., not tested; PV, polycythemia vera.

## Table S10

## Effects of anti-neoplastic drugs on survival of MPN cells

| Cells | Apoptosis-inducing efficacy - ED_50_ values | | | | | | | |
| --- | --- | --- | --- | --- | --- | --- | --- | --- |
|  | Ruxolitinib  [µM] | Fedratinib  [µM] | Avapritinib  [µM] | Midostaurin  [µM] | Pelabresib  [µM] | JQ1  [µM] | dBET6  [µM] | GO  [µg/ml] |
| CD34^+^/CD38^−^ stem cells | >10 | 5-10 | >10 | >10 | >10 | >10 | 0.5 | >25 |
| HEL | >25 | 1-2.5 | >25 | >25 | >10 | >10 | >10 | >25 |
| SET-2 | >25 | 1-2.5 | >25 | >25 | >10 | 5-10 | >10 | >25 |
| UT-7 WT | 10-25 | 1-2.5 | >25 | >25 | >10 | >10 | >10 | n.t. |
| UT-7 B5 | >25 | 2.5-5 | >25 | >25 | >10 | >10 | 0.5-1 | n.t. |

MPN-related cell lines (HEL, SET-2, UT-7 cells expressing wild type *CALR* (WT) or *CALR* del61/del25 mutation (B5)) or MNC isolated from the bone marrow or peripheral blood of patients with MPN (ET, n=4; PV, n=1; MF, n=4) were incubated with increasing concentrations of targeted drugs as indicated. The percentage of apoptotic cells was measured after 24 hours by AnnexinV/DAPI staining and flow cytometry. The table shows ED_50_ values obtained for each compound in the cell lines or in primary MNC samples. Abbreviations: ED_50_, half maximal effective dose; ET, essential thrombocythemia; GO, gemtuzumab-ozogamicin; MF, myelofibrosis; MNC, mononuclear cells; MPN, myeloproliferative neoplasms; n.t., not tested; PV, polycythemia vera.

**References**

1 Barbui T, Thiele J, Gisslinger H et al. The 2016 WHO classification and diagnostic criteria for myeloproliferative neoplasms: document summary and in-depth discussion. *Blood Cancer J.* 2018;8(2):15.

2 Khoury JD, Solary E, Abla O, et al. The 5th edition of the world health organization classification of haematolymphoid tumours: myeloid and histiocytic/dendritic neoplasms. *Leukemia*. 2022;36(7):1703-1719.

3 Jia R, Balligand T, Atamanyuk V, et al. Hematoxylin binds to mutant calreticulin and disrupts its abnormal interaction with thrombopoietin receptor. *Blood*. 2021;137(14):1920-1931.

4 Herrmann H, Sadovnik I, Cerny-Reiterer S, et al. Dipeptidylpeptidase IV (CD26) defines leukemic stem cells (LSC) in chronic myeloid leukemia. *Blood*. 2014;123(25):3951-3962.

5 Sadovnik I, Herrmann H, Eisenwort G, et al. Expression of CD25 on leukemic stem cells in BCR-ABL1+ CML: Potential diagnostic value and functional implications. *Exp Hematol*. 2017;51:17-24.

6 Herrmann H, Sadovnik I, Eisenwort G, et al. Delineation of target expression profiles in CD34+/CD38− and CD34+/CD38+ stem and progenitor cells in AML and CML. *Blood Adv*. 2020;4(20):5118-5132.

7 Hadzijusufovic E, Keller A, Berger D, et al. STAT5 is expressed in CD34+/CD38− stem cells and serves as a potential molecular target in Ph-negative myeloproliferative neoplasms. *Cancers (Basel)*. 2020;12(4):1021.

8 Hu Y, Smyth GK. ELDA: extreme limiting dilution analysis for comparing depleted and enriched populations in stem cell and other assays. *J Immunol Methods* 2009;347(1-2):70–78.
